# ASYNAPSIS3 has diverse dosage-dependent effects on meiotic crossover formation in *Brassica napus*

**DOI:** 10.1093/plcell/koae207

**Published:** 2024-07-24

**Authors:** Lei Chu, Jixin Zhuang, Miaowei Geng, Yashi Zhang, Jing Zhu, Chunyu Zhang, Arp Schnittger, Bin Yi, Chao Yang

**Affiliations:** National Key Laboratory of Crop Genetic Improvement, Hubei Hongshan Laboratory, Huazhong Agricultural University, Wuhan 430070, China; National Key Laboratory of Crop Genetic Improvement, Hubei Hongshan Laboratory, Huazhong Agricultural University, Wuhan 430070, China; National Key Laboratory of Crop Genetic Improvement, Hubei Hongshan Laboratory, Huazhong Agricultural University, Wuhan 430070, China; National Key Laboratory of Crop Genetic Improvement, Hubei Hongshan Laboratory, Huazhong Agricultural University, Wuhan 430070, China; National Key Laboratory of Crop Genetic Improvement, Hubei Hongshan Laboratory, Huazhong Agricultural University, Wuhan 430070, China; National Key Laboratory of Crop Genetic Improvement, Hubei Hongshan Laboratory, Huazhong Agricultural University, Wuhan 430070, China; Department of Developmental Biology, Institute of Plant Science and Microbiology, University of Hamburg, Hamburg 22609, Germany; National Key Laboratory of Crop Genetic Improvement, Hubei Hongshan Laboratory, Huazhong Agricultural University, Wuhan 430070, China; National Key Laboratory of Crop Genetic Improvement, Hubei Hongshan Laboratory, Huazhong Agricultural University, Wuhan 430070, China

## Abstract

Crossovers create genetic diversity and are required for equal chromosome segregation during meiosis. Crossover number and distribution are highly regulated by different mechanisms that are not yet fully understood, including crossover interference. The chromosome axis is crucial for crossover formation. Here, we explore the function of the axis protein ASYNAPSIS3. To this end, we use the allotetraploid species *Brassica napus*; due to its polyploid nature, this system allows a fine-grained dissection of the dosage of meiotic regulators. The simultaneous mutation of all 4 *ASY3* alleles results in defective synapsis and drastic reduction of crossovers, which is largely rescued by the presence of only one functional *ASY3* allele. Crucially, while the number of class I crossovers in mutants with 2 functional *ASY3* alleles is comparable to that in wild type, this number is significantly increased in mutants with only one functional *ASY3* allele, indicating that reducing ASY3 dosage increases crossover formation. Moreover, the class I crossovers on each bivalent in mutants with 1 functional *ASY3* allele follow a random distribution, indicating compromised crossover interference. These results reveal the distinct dosage-dependent effects of ASY3 on crossover formation and provide insights into the role of the chromosome axis in patterning recombination.

## Teaser

A key component of the meiotic chromosome axis modulates the synapsis and strength of crossover interference, thereby exerting distinct dosage-dependent effects on chromosome recombination.

## Introduction

Crop breeding relies on selecting elite varieties that harbor desirable combinations of genetic alleles. The combination of these alleles is accomplished in meiosis through a new assortment of maternal and paternal chromosomes as well as an exchange of segments of the parental chromosomes through crossovers (COs; Mercier et al. 2015). However, COs are limited and, especially in crops, not equally distributed, leading to linkage drag that limits the success of breeding schemes (Mieulet et al. 2018; Blary and Jenczewski 2019). Thus, understanding CO patterning mechanisms is a long-standing goal in plant biotechnology.

COs are also crucial for accurate chromosome segregation during meiosis I. Each pair of homologous chromosomes (homologs) needs to form at least 1 crossover, the so-called obligate CO, controlled by a mechanism known as CO assurance. In addition, class I COs are subject to tight regulation that prevents COs from occurring close to each other, a phenomenon called CO interference, which contributes to linkage drag (Lambing et al. 2017; Wang et al. 2021). Limiting breeding efforts, the underlying molecular mechanisms leading to CO assurance and interference are not fully understood.

COs are generated through the repair of programmed DNA double-strand breaks (DSBs) via 2 recombination pathways (Wang and Copenhaver 2018). One pathway depends on the group of meiosis-specific ZMM proteins (Zipper 1-4 (Zip1-4), MutS homolog 4-5 (MSH4-5), and Mer3 in yeast (*Saccharomyces cerevisiae*); SHORTAGE IN CHIASMA 1 (SHOC1/Zip2), ENHANCER OF CELL INVASION N°10 (HEI10/Zip3), HEI10 INTERACTION PROTEIN 1 (HEIP1), PARTING DANCERS (PTD/Spo16), ZIP4, MSH4-5, and MER3 in Arabidopsis (*Arabidopsis thaliana*)) that catalyze class I COs, representing the major class of COs (85% to 90% in Arabidopsis, 70% to 90% in rice (*Oryza sativa*)) (Mercier et al. 2005; Chelysheva et al. 2007, 2012; Higgins et al. 2008; Macaisne et al. 2008; Wang et al. 2012; Zhang et al. 2014a; Mercier et al. 2015; Li et al. 2018; Ren et al. 2019; Singh et al. 2023; Thangavel et al. 2023) . The other pathway relies on structure-specific nucleases, e.g. MMS AND UV SENSITIVE 81 (MUS81) and a homolog of Fanconi Anemia Complementation Group D2 (FANCD2) in Arabidopsis, and promotes the remaining, so-called class II COs, which are not limited by interference (Berchowitz et al. 2007; Kurzbauer et al. 2017).

CO formation depends highly on the chromosome axis, a proteinaceous structure assembled along the entire length of each pair of sister chromatids at early meiosis that later transforms into the lateral element of the synaptonemal complex (SC) (Zickler and Kleckner 1999, 2015; Blat et al. 2002; Panizza et al. 2011; Hunter 2015; Wang and Copenhaver 2018; Ito and Shinohara 2023). The cohesin complexes, encompassing the duplicated sister chromatids, are thought to build the basis of the axis in many species including plants (Bhatt et al. 1999; Golubovskaya et al. 2006; Shao et al. 2011). On top of cohesin complexes, at least 3 additional proteins are loaded, including the HORMA domain-containing protein (HORMAD) ASYNAPSIS1 (ASY1 [homolog of Hop1 in yeast, HORMAD1/2 in mammals]), and 2 coiled-coil proteins known as the “axis core” that affect ASY1 localization, i.e. the linker protein ASY3 (homolog of Red1 in yeast, SYCP2 in mammals) and ASYNAPSIS4 (ASY4 [homolog of SYCP3 in mammals]; Hollingsworth and Johnson 1993; Lammers et al. 1994; Ross et al. 1997; Armstrong et al. 2002; Wojtasz et al. 2009; Fukuda et al. 2010; Ferdous et al. 2012; Chambon et al. 2018).

The chromosome axis tethers and organizes the sister chromatids to form a higher-order structure of linear DNA loop-arrays and promotes efficient DSB formation, interhomolog-biased repair, and synaptonemal complex installation (Hollingsworth and Ponte 1997; Niu et al. 2005; Carballo et al. 2008; Goodyer et al. 2008; Hunter 2015; Xue et al. 2019; Lambing et al. 2020a,b). Plants lacking the axis components, e.g. ASY1, ASY3, ASY4, or the α-kleisin subunit of cohesin REC8, show univalents in metaphase I meiocytes and/or altered CO distribution, highlighting the importance of chromosome axis for CO patterning (Ross et al. 1997; Sanchez-Moran et al. 2007; Boden et al. 2009; Daniel et al. 2011; Ferdous et al. 2012; Chambon et al. 2018; Lambing et al. 2020b; Cuacos et al. 2021; Pochon et al. 2022; Dio et al. 2023).

A crucial question concerning CO regulation is how CO assurance and interference are balanced. Recent studies show that loss of the transverse filament protein of the SC, ZYP1 in Arabidopsis, compromises CO assurance and abolishes interference, resulting in an ∼50% increase in class I COs, accompanied by ∼10% to 20% of metaphase I cells containing 1 pair of univalents (Capilla-Pérez et al. 2021; France et al. 2021; Yang et al. 2022). Nonetheless, the question of to which extent this increase of class I COs is attributed to the loss of ZYP1 per se or to a defective remodeling of the chromosome axis in general remains obscure (Yang et al. 2022).

Besides ZYP1, previous studies also provide insight into the role of ASY1 in CO assurance and interference (Lambing et al. 2020a; Pochon et al. 2022). In Arabidopsis *asy1* mutants which have a severely defective synapsis, CO interference is reported to be undetectable, and COs mainly locate and cluster at telomeric regions in contrast to more widely spaced COs in the wild type (Lambing et al. 2020a; Pochon et al. 2022). A recent study investigated the function of ASY1 in tetraploid wheat and found that the chiasma number shows a gradual decrease as the reduction of *ASY1* alleles, substantiating the importance of ASY1 in the global CO formation (see more in discussion; Dio et al. 2023). Nevertheless, we have less knowledge about the function of the “axis core” protein ASY3 in patterning recombination.

Here, we carry out a fine-grained dissection for the function of chromosome axis protein ASY3 in CO formation by making use of the tetraploid nature of *Brassica napus* which harbors 2 copies of *ASY3* and thus 4 alleles offering the possibility to address the dosage dependency of ASY3 and other regulators. Cytological analyses in a series of *asy3* mutants where different numbers of *ASY3* alleles are mutated show that the simultaneous mutation of all 4 *ASY3* alleles results in defective HEI10 loading and synapsis, and drastic reduction of chiasmata, which is largely rescued by the presence of only one functional *ASY3* allele, supporting the key role of ASY3 in implementing COs (Ferdous et al. 2012).

Strikingly, while the number of class I COs in mutants with 2 functional *ASY3* alleles is comparable to that in the wild type, this number is significantly increased in mutants with 1 functional *ASY3* allele, indicating that reducing ASY3 dosage increases CO formation. Moreover, the class I COs on each bivalent in mutants with 1 functional *ASY3* allele follow a Poisson-type distribution, indicating a strong attenuation of CO interference. Our results demonstrate the dosage-dependent distinct effects of ASY3 on CO formation and provide insights into the function and mechanism of chromosome axis/SC in CO patterning.

## Results

### Generation of *asy3* mutants in *Brassica napus*

By performing BLAST analysis using the *Brassica napus* multi-omics information resource database (https://yanglab.hzau.edu.cn/BnIR [Song et al. 2020; Yang et al. 2023]), 2 homologs (termed BnaASY3) of the Arabidopsis ASY3 protein were identified in *Brassica napus*. Each A and C sub-genomes contains 1 homolog: referred to as *BnaA05.ASY3* (*BnaA05g00870D*) located at chromosome A05 and *BnaC04.ASY3* (*BnaC04g00500D*) at chromosome C04. *BnaA05.ASY3* and *BnaC04.ASY3* exhibit a protein identity of 93.74% between each other, which share 75.13% and 75.16% protein identity with AtASY3, respectively (Supplementary Fig. S1A). Based on the transcriptomic data from the BnIR database (Liu et al. 2021), the transcription level of *BnaC04.ASY3* is higher than that of *BnaA05.ASY3* in most tissues including flower buds at different developmental stages (Supplementary Fig. S1A), which is confirmed by the RT-qPCR in anthers at the meiotic stage (Supplementary Fig. S1B).

**Figure 1.**
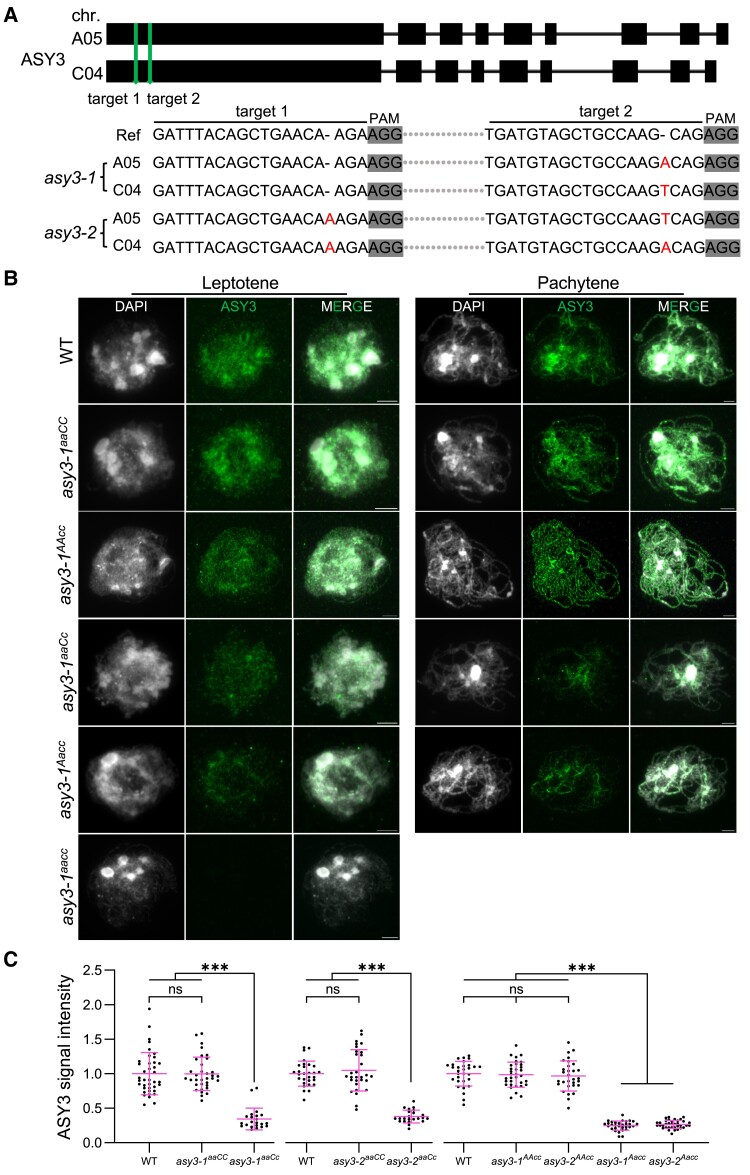
Generation of *asy3* null and dosage-reduced mutants in *Brassica napus*. **A)** Generation and identification of 2 *asy3* mutant lines by CRISPR-Cas9. Red letters indicate the insertion mutations for BnaA05.ASY3 and BnaC04.ASY3 in *asy3-1* and *asy3-2* mutants. **B)** Immunostaining of ASY3 in male meiocytes of WT, *asy3-1^aaCC^*, *asy3-1^AAcc^*, *asy3-1^aaCc^*, *asy3-1^Aacc^*, and *asy3-1^aacc^* mutant plants at leptotene and pachytene (or -like). Bars: 5 *µ*m. **C)** Relative ASY3 signal intensity in WT, *asy3-1^aaCC^*, *asy3-1^aaCc^*, *asy3-2^aaCC^*, *asy3-2^aaCc^*, *asy3-1^AAcc^*, *asy3-2^AAcc^*, *asy3-1^Aacc^*, and *asy3-2^Aacc^* mutant plants at leptotene. The comparisons of signal intensity of WT with *asy3-1^aaCC^* and *asy3-1^aaCc^*, WT with *asy3-2^aaCC^* and *asy3-2^aaCc^*, WT with *asy3-1^AAcc^*, *asy3-2^AAcc^*, *asy3-1^Aacc^*, and *asy3-2^Aacc^* were plotted independently. Error bars indicate mean ± SD. Asterisks indicate significant differences (Game–Howell's multiple comparisons test, *P* < 0.001).

To explore the function of ASY3, we applied the CRISPR/Cas9-based gene editing approach in the spring-type cultivar *Westar* and identified from the 30 T0 generation of transformants 2 independent insertion mutant lines where monoallelic mutations are identified in both *BnaA05.ASY3* and *BnaC04.ASY3* genes (called *Bnaasy3-1* and *Bnaasy3-2*; Fig. 1A). The insertions in both *Bnaasy3-1* and *Bnaasy3-2* mutation lines result in premature translational termination of 2 *ASY3* copies, producing likely only truncated versions that contain only amino acids of the N-terminal regions (Supplementary Fig. S1C, red asterisks). For each line, we obtained different genotypes from a T1 segregating population, i.e. the mutants with 4 alleles of *ASY3* mutated (called *asy3-1^aacc^* and *asy3-2^aacc^*), mutants having only the 2 *ASY3* alleles of the A sub-genome mutated (called *asy3-1^aaCC^* and *asy3-2^aaCC^*), and mutants having the 2 *ASY3* alleles of the A sub-genome plus 1 allele of the C sub-genome mutated (named *asy3-1^aaCc^* and *asy3-2^aaCc^*). Notably, for all 30 T0 transformants, the 2 *ASY3* alleles of the A sub-genome harbor either no mutation or homozygous mutation (monoallelic or biallelic mutations). Therefore, to obtain such mutants of *asy3^AAcc^* and *asy3^Aacc^*, we crossed the *asy3-1^aaCc^* and *asy3-2^aaCc^* to the wild type and identified the relevant mutants from the F2 progeny (named *asy3-1^AAcc^, asy3-2^AAcc^, asy3-1^Aacc^,* and *asy3-2^Aacc^*).

To study the level of ASY3 accumulation in these different *asy3* mutants, we performed quantitative immunostaining analyses with an ASY3 antibody in male meiocytes (see Materials and Methods). The absence of the signal of ASY3 in *asy3-1^aacc^* and *asy3-2^aacc^* mutants validates the specificity of the antibody and confirms the complete loss of ASY3 function (Fig. 1B). In the wild type, ASY3 accumulates along the chromosome axis at leptotene and at pachytene, 2 ASY3-labeled axes of the homologous chromosomes co-align and synapse resulting in thicker threads clearly visible in the immunostainings (Fig. 1B). A similar ASY3 localization pattern was detected in *asy3^aaCC^* and *asy3^AAcc^* mutant alleles (Fig. 1B, Supplementary Fig. S2A). In *asy3^aaCc^* and *asy3^Aacc^* mutants, where only one *ASY3* allele is functional, the thread-like structures of ASY3 were still observed along chromosomes at leptotene. However, the signal intensity of ASY3 in *asy3^aaCc^* and *asy3^Aacc^* was clearly weakened compared to the wild type, *asy3^aaCC^*, and *asy3^AAcc^* (Fig. 1B, Supplementary Fig. S2A). This reduction of the signal intensity of ASY3 in *asy3^aaCc^* and *asy3^Aacc^* persists in pachytene (or -like) cells where the ASY3 signal shows a patchier pattern compared to the wild type, *asy3^aaCC^*, and *asy3^AAcc^* plants (Fig. 1B, Supplementary Fig. S2A).

We quantified the signal intensity of ASY3 during leptotene and pachytene (or -like) and observed a significant reduction of ASY3 dosage in *asy3^aaCc^* (∼ 65.77% decrease at leptotene, ∼ 61.71% at pachytene for *asy3-1^aaCc^*; ∼ 61.99% decrease at leptotene, ∼ 65.32% at pachytene for *asy3-2^aaCc^*) and *asy3^Aacc^* (∼ 75.29% decrease at leptotene, ∼ 69.48% at pachytene for *asy3-1^Aacc^*; ∼ 73.48% decrease at leptotene, ∼ 68.06% at pachytene for *asy3-2^Aacc^*) compared to the wild type (Game–Howell's multiple comparisons test, *P* < 0.001). Notably, the slightly stronger decrease of ASY3 dosage in *asy3^Aacc^* is consistent with the higher transcriptional level of *BnaC04.ASY3* than *BnaA05.ASY3* (Supplementary Fig. S1B and C). No significant difference was found between genotypes of *asy3^aaCC^*, *asy3^AAcc^*, and wild type at both leptotene and pachytene nuclei (Fig. 1C, Supplementary Fig. S2B).

### ASY3 dosage-dependent effects on the chromosome localization of ASY1

Arabidopsis ASY3 and its orthologs in other organisms, e.g. yeast, mouse, and rice, are required for proper chromosome recruitment and localization of the HORMAD protein ASY1 through physical interaction of the N-terminal domain of ASY3 (known as closure motif, indicated in Supplementary Fig. S1A) and the HORMA domain of ASY1 (Kolas et al. 2004; Wang et al. 2011; Rosenberg and Corbett 2015; West et al. 2018, 2019; Yang et al. 2020a,b). We confirmed that this interaction of the BnaASY3 closure motif (1-32 aa) to the BnaASY1 HORMA domain (1-300 aa) is conserved in *Brassica napus* by using the yeast 2-hybrid and split-luciferase complementation assays (Supplementary Fig. S3), supporting the potential role of ASY3 in ASY1 localization.

**Figure 2.**
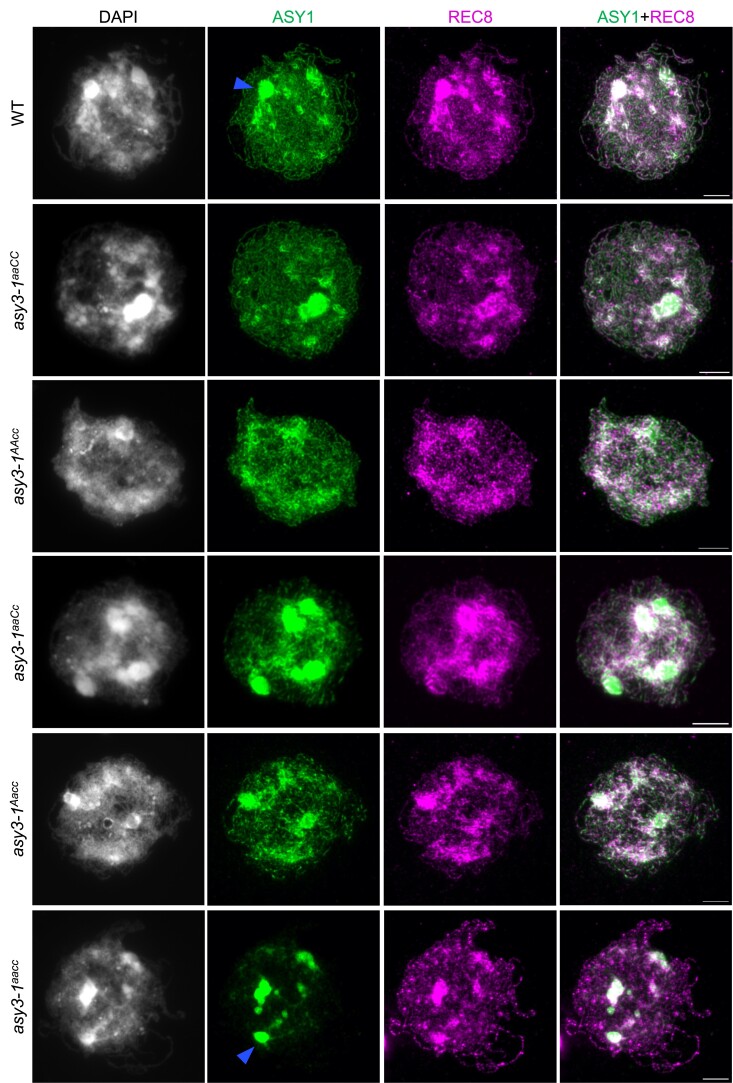
Immunolocalization of ASY1 and REC8 in male meiocytes of wild-type, *asy3-1^aaCC^*, *asy3-1^AAaa^*, *asy3-1^aaCc^*, *asy3-1^Aacc^*, and *asy3-1^aacc^* mutant plants at early prophase I (leptotene or leptotene-like). Blue arrow indicates the “blob”-like signal from densely DAPI-staining chromosome regions that were overexposed and thus removed from the quantification of signal intensity shown in SupplementaryFig. 6B. Bars: 5 *µ*m. The scale bar in the merge represents all images in the same row.

Next, to investigate the detailed effects of BnaASY3 on ASY1 localization, we performed an immunostaining analysis of male meiocytes using an antibody against ASY1. We verified the specificity of the ASY1 antibody using Arabidopsis *asy1-4* mutants (Supplementary Fig. S4A). In wild-type *Brassica napus*, ASY1 forms linear structures along the chromosomes at leptotene and follows largely the REC8-labelled axes (Fig. 2). In *asy3^aacc^*, REC8 localization is normal whereas ASY1 is strongly diminished on most of the chromosome regions, consistent with an important role of ASY3 for proper recruitment and/or extension of ASY1 on chromosomes. Notably, a weak signal of ASY1 was still detected on chromosome arms, showing a pattern of dotty signal or non-linear structures of short stretches. This observation is consistent with a previous report in Arabidopsis *asy3* mutant, indicating that ASY1 may be able to bind to DNA independently of ASY3, as its homolog HOP1 in yeast (Kironmai et al. 1998; Khan et al. 2013; Milano et al. 2024).

Notably, a strong accumulation of ASY1 and REC8 in the wild type was detected at regions that are more brightly stained with DAPI indicative of the chromocenters comprising of centromeric and pericentromeric DNA (Fig. 2), as confirmed by the co-immunostaining of ASY1 with CENH3 (a centromere specific histone protein) (Supplementary Fig. S5A). This is reminiscent of the previous studies showing that ASY1 and REC8 exhibit strong enrichments at the heterochromatin in Arabidopsis (Lambing et al. 2020a,b). Unexpectedly, the accumulation of ASY1 at the densely DAPI-stained heterochromatic regions was not visibly affected by the absence of ASY3 (Fig. 2, Supplementary Fig. S5A, blue arrowheads). We quantified the signal intensity of ASY1 from the densely DAPI-stained regions with non-overexposed images at leptotene (see Materials and Methods) and found no difference between the wild type and *asy3^aacc^* mutants, suggesting that ASY1 loading at the chromocenters is independent of ASY3 in *Brassica napus* (Supplementary Fig. S5B and C).

We further investigated the chromosome localization of ASY1 in genotypes of *asy3^aaCC^*, *asy3^AAcc^*, *asy3^aaCc^*, and *asy3^Aacc^* plants. We found that in *asy3^aaCC^* and *asy3^AAcc^*, ASY1 is indistinguishably loaded in comparison to the wild type (Fig. 2, Supplementary Fig. S6A). This result is consistent with the quantification of ASY3 dosage in *asy3^aaCC^* and *asy3^AAcc^* (Fig. 1C). However, in *asy3^aaCc^* and *asy3^Aacc^* mutants where ASY3 dosage is reduced to ∼30% to 40% of that in wild type, ASY1 shows a patchier and less continuous pattern with reduced signal intensity (Fig. 2, Supplementary Fig. S6A). The quantification shows that while the protein level of ASY1 on chromosomes is not altered in *asy3^aaCC^* and *asy3^AAcc^*, it is reduced to 58.92% and 54.58% of the wild-type level in *asy3-1^aaCc^* and *asy3-2^aaCc^*, respectively (Supplementary Fig. S6B). In *asy3^Aacc^* mutants where ASY3 dosage is even less, the chromosome-associated ASY1 is reduced to 32.75% and 34.24% of the wild-type level in *asy3-1^Aacc^* and *asy3-2^Aacc^*, respectively (Supplementary Fig. S6B).

### ASY3 exhibits a dosage-dependent impact on meiosis and plant fertility

**Figure 3.**
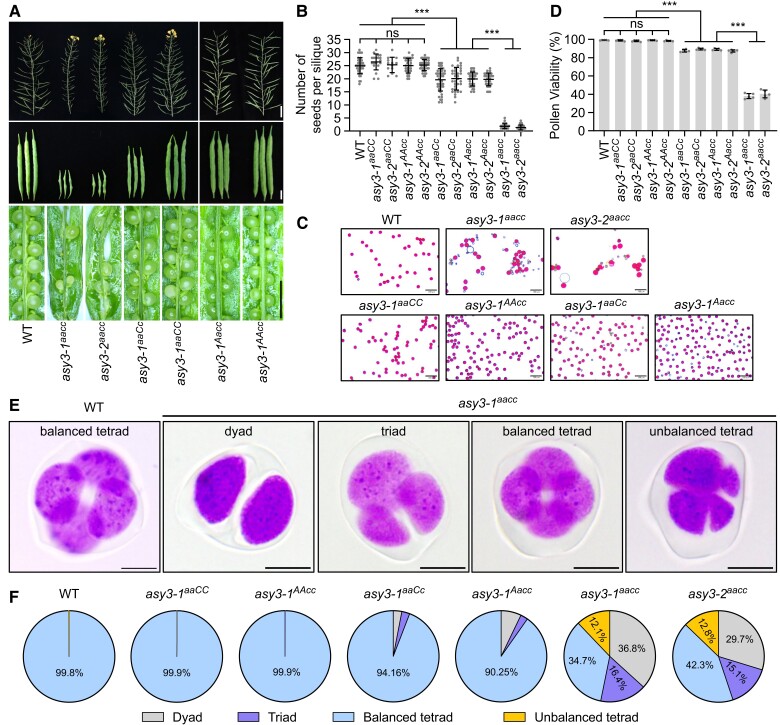
Phenotypic analysis of *asy3* mutants. **A)** Main branches, siliques, and seed-sets in WT, *asy3-1^aacc^*, *asy3-2^aacc^*, *asy3-1^aaCc^*, *asy3-1^aaCC^*, *asy3-1^Aacc^*, and *asy3-1^AAcc^* mutant plants. Bars from top to bottom panels are 5 cm, 1cm, and 5 mm, respectively. The scale bar represents all images in the same row. **B)** Statistical analysis for the number of seeds per silique in WT, *asy3-1^aaCC^*, *asy3-2^aaCC^*, *asy3-1^AAcc^*, *asy3-2^AAcc^*, *asy3-1^aaCc^*, *asy3-2^aaCc^*, *asy3-1^Aacc^*, *asy3-2^Aacc^*, *asy3-1^aacc^*, and *asy3-2^aacc^* mutant plants. At least 13 siliques from different plants were dissected and counted for each genotype. **C)** Pollen staining of WT, *asy3-1^aacc^*, *asy3-2^aacc^*, *asy3-1^aaCC^*, *asy3-1^AAcc^*, *asy3-1^aaCc^*, and *asy3-1^Aacc^* mutant plants. Red staining indicates viable pollen and blue indicates dead pollen. Five plants were examined for each genotype. Bars: 100 *µ*m. **D)** Pollen viability of WT, *asy3-1^aaCC^*, *asy3-2^aaCC^*, *asy3-1^AAcc^*, *asy3-2^AAcc^*, *asy3-1^aaCc^*, *asy3-2^aaCc^*, *asy3-1^Aacc^*, *asy3-2^Aacc^*, *asy3-1^aacc^*, and *asy3-2^aacc^* mutant plants. At least 4000 pollen grains were counted from different flowers for each genotype. **E)** Examples of the staining of male meiotic products in WT and *asy3-1^aacc^* mutant plants. Bars: 5 *µ*m. **F)** Pie charts showing the proportion of balanced tetrad, unbalanced tetrad, triad, and dyad in WT, *asy3-1^aaCC^*, *asy3-1^AAcc^*, *asy3-1^aaCc^*, *asy3-1^Aacc^*, *asy3-1^aacc^*, and *asy3-2^aacc^*, mutant plants. Error bars in **B** and **D**) indicate mean ± SD and asterisks indicate significant difference (Game–Howell's multiple comparisons test, *P* < 0.001).

We next analyzed the dosage-dependent effect of ASY3 on plant fertility. As expected, the absence of ASY3 does not affect plant growth and development until flowering, consistent with a meiosis-specific role of ASY3 (Supplementary Fig. S7A [Yuan et al. 2009; Wang et al. 2011; Ferdous et al. 2012]). At the reproductive stage, the silique length becomes very short in *asy3^aacc^* mutants and only very few viable seeds per silique are produced in comparison to the wild type (average of 1.83 in *asy3-1^aacc^* and 1.37 in *asy3-2^aacc^* vs 24.98 in wild type [Fig. 3, A and B]). This reduced silique length is partially rescued by the presence of 1 functional *ASY3* allele in both *asy3^aaCc^* and *asy3^Aacc^* genotypes. Accordingly, the seed-set is also significantly increased in *asy3^aaCc^* and *asy3^Aacc^* compared to *asy3^aacc^* mutants, but remaining lower than that in wild type (average of 19.60 in *asy3-1^aaCc^*, 20.03 in *asy3-2^aaCc^*, 19.91 in *asy3-1^Aacc^* and 19.76 in *asy3-2^Aacc^* vs 24.98 in wild type, *P* < 0.001, Game–Howell's multiple comparisons test [Fig. 3, A and B]). In *asy3^aaCC^* and *asy3^AAcc^* mutants, the silique length and seed number per silique are comparable to that in the wild type, suggesting that BnaA05.ASY3 and BnaC04.ASY3 are equally functional (Fig. 3, A and B).

Staining pollen with Peterson solution revealed a similar ASY3 dosage-dependent impact on pollen viability (average 99.15% in WT vs 98.85% in *asy3-1^aaCC^*, 98.25% in *asy3-2^aaCC^*, 99.01% in *asy3-1^AAcc^*, 98.40% in *asy3-2^AAcc^*, 87.63% in *asy3-1^aaCc^*, 89.36% in *asy3-2^aaCc^*, 89.15% in *asy3-1^Aacc^*, 87.40% in *asy3-2^Aacc^*, 37.98% in *asy3-1^aacc^* and 40.40% in *asy3-2^aacc^* [Fig. 3, C and D, Supplementary Fig. S7B]). Subsequent tetrad analysis in male meiosis showed a severe disruption of meiotic products in *asy3^aacc^* mutants (65.27% abnormal tetrad in *asy3-1^aacc^*, and 57.66% in *asy3-2^aacc^* vs 0.19% in wild type [Fig. 3, E and F, Supplementary Fig. S7C and D]). This defect in tetrad formation was largely reversed by the presence of 1 functional *ASY3* allele in both *asy3^aaCc^* and *asy3^Aacc^*, producing only a small portion of defective tetrads compared to wild type (5.84% in *asy3-1^aaCc^*, 9.52% in *asy3-2^aaCc^*, 9.75% in *asy3-1^Aacc^*, and 6.70% in *asy3-2^Aacc^* vs 0.19% in wild type). In *asy3^aaCC^* and *asy3^AAcc^* mutants, the tetrad formation is indistinguishable from that in the wild type (Fig. 3, E and F, Supplementary Fig. S7C and D).

### Chromosome synapsis is sensitive to ASY3 dosage

**Figure 4.**
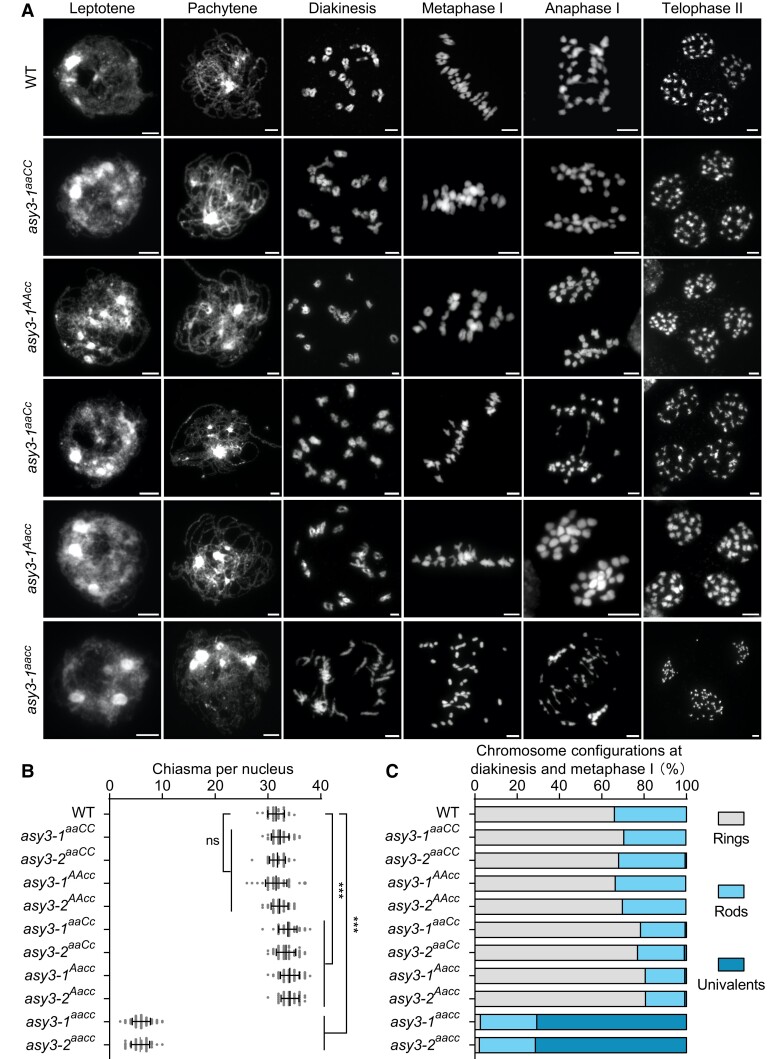
Analysis of meiotic chromosome behavior in wild-type and *asy3* mutants. **A)** Chromosome spread analysis of male meiosis in WT, *asy3-1^aaCC^*, *asy3-1^AAcc^*, *asy3-1^aaCc^*, *asy3-1^Aacc^*, and *asy3-1^aacc^* mutant plants throughout meiosis. Flower buds from at least 10 plants were collected and analyzed for each genotype. Bars: 5 *µ*m. **B)** Scatter dot plot of the estimation of minimum chiasma number in WT (*n* = 32 cells), *asy3-1^aaCC^* (*n* = 40 cells), *asy3-2^aaCC^* (*n* = 44 cells), *asy3-1^AAcc^* (*n* = 54 cells), *asy3-2^AAcc^*(*n* = 43 cells), *asy3-1^aaCc^* (*n* = 51 cells), *asy3-2^aaCc^* (*n* = 44 cells), *asy3-1^Aacc^* (*n* = 53 cells), *asy3-2^Aacc^* (*n* = 40 cells), *asy3-1^aacc^* (*n* = 100 cells), and *asy3-2^aacc^* (*n* = 42 cells) mutant plants. The statistical analysis was performed by 1-way ANOVA with Games–Howell's multiple comparisons test. Error bars indicate mean ± SD. ***, *P* < 0.001. **C)** Distribution of chromosome configurations (ring, rod, or univalent) at diakinesis/metaphase I in WT (*n* = 32 cells), *asy3-1^aaCC^* (*n* = 40 cells), *asy3-2^aaCC^* (*n* = 44 cells), *asy3-1^AAcc^* (*n* = 54 cells), *asy3-2^AAcc^*(*n* = 43 cells), *asy3-1^aaCc^* (*n* = 51 cells), *asy3-2^aaCc^* (*n* = 44 cells), *asy3-1^Aacc^* (*n* = 53 cells), *asy3-2^Aacc^* (*n* = 40 cells), *asy3-1^aacc^* (*n* = 100 cells), and *asy3-2^aacc^* (*n* = 42 cells) mutant plants. The exact numbers of cell counted in **A–D**) were shown in the relevant texts.

For a detailed analysis of meiosis, we spread the chromosomes of male meiocytes. In the wild type, the DAPI-stained chromatin exhibits a thin filament-like structure at leptotene and starts to pair with their homologs during zygotene (Fig. 4A). As cells progress to pachytene, homologous chromosomes are coaligned and synapsed (*n* = 36 cells). In *asy3^aacc^* mutants, the chromosomes also show a thread-like structure at leptotene (Fig. 4A, Supplementary Fig. S8A). However, similar to Arabidopsis and rice *asy3/pair3* mutants (Yuan et al. 2009; Wang et al. 2011; Ferdous et al. 2012), normal pachytene nuclei were never observed, and homologous chromosomes remain largely not coaligned (*n* = 50 cells in *asy3-1^aacc^* and *n* = 31 in *asy3-2^aacc^* [Fig. 4A, Supplementary Fig. S8A]).

In genotypes of *asy3^aaCC^* and *asy3^AAcc^*, chromosome juxtaposition at pachytene seems to be properly achieved in a large proportion of cells (79.31%, *n* = 58 cells in *asy3-1^aaCC^*, and 80.70%, *n* = 57 in *asy3-2^aaCC^*; 77.94%, *n* = 68 in *asy3-1^AAcc^*, and 80.77%, *n* = 52 in *asy3-2^AAcc^* [Fig. 4A, Supplementary Fig. S8A]), with the remaining cells showing only small regions of unpaired stretches (red arrows in Supplementary Fig. S8B). In *asy3^aaCc^* and *asy3^Aacc^* mutants, defects in chromosome coalignment and synapsis were more frequently observed and all pachytene cells showed some unpaired single chromosome threads while most of the chromosome regions were able to synapse (*n* = 48 cells in *asy3-1^aaCc^* and *n* = 51 in *asy3-2^aaCc^*; *n* = 53 in *asy3-1^Aacc^* and *n* = 55 in *asy3-2^Aacc^* [Fig. 4A, Supplementary Fig. S8A]). Noting that the ratio of cells with properly/fully coaligned chromosomes could be overestimated due to the low resolution of the DAPI-stained chromosomes.

**Figure 5.**
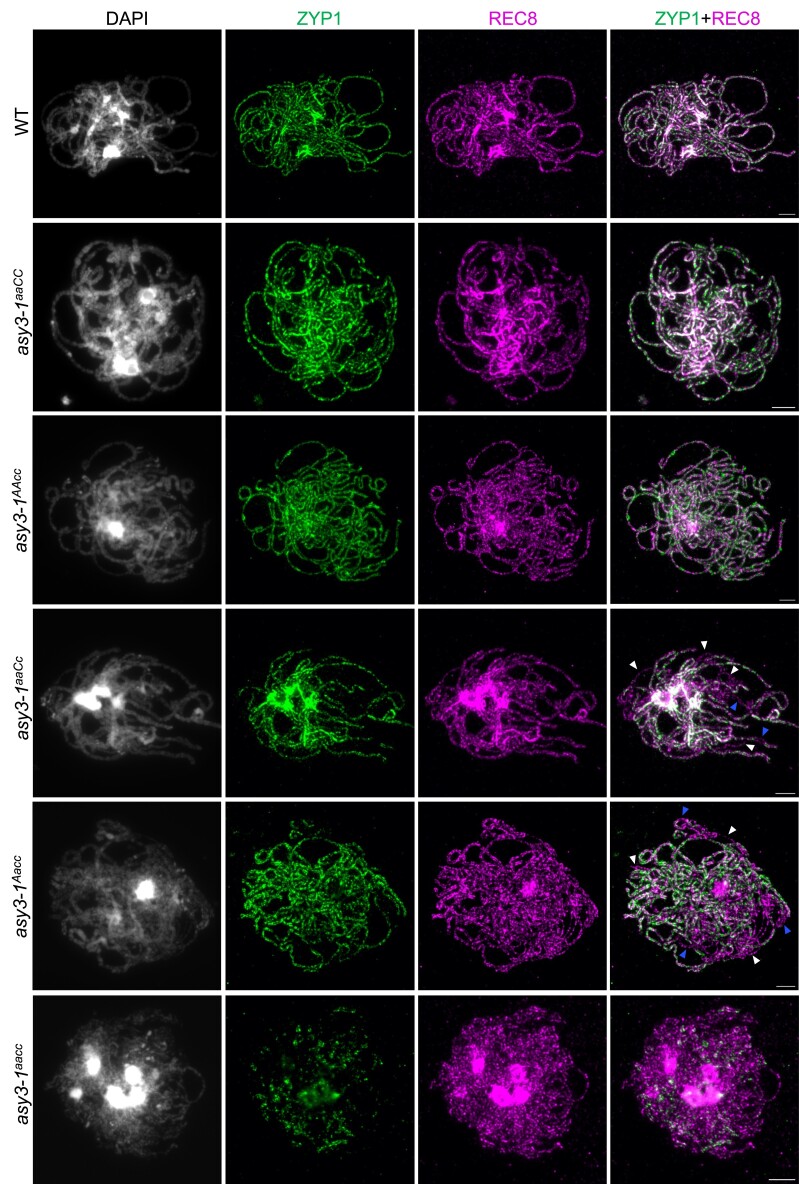
ASY3 dosage-dependent effects on chromosome synapsis. Co-immunolocalization of ZYP1 and REC8 at pachytene in male meiocytes of WT, *asy3-1^aaCC^*, *asy3-1^AAcc^*, *asy3-1^aaCc^*, *asy3-1^Aacc^*, and *asy3-1^aacc^* mutant plants. White and Blue arrowheads indicate the single threads or coaligned regions that both have no ZYP1 signal, respectively. Bars: 5 *µ*m.

To complement the above analysis, we performed co-immunolocalization of ZYP1 and REC8/ASY3 in male meiosis at pachytene. In the wild type, ZYP1 is assembled along the entire chromosome length (*n* = 14 cells [Fig. 5]), which is located between 2 coaligned REC8/ASY3 axes, as shown by the super-resolution images acquired by STED (Supplementary Fig. S9). Corresponding to what we observed in chromosome spreads stained with DAPI, while a wild-type-like linear REC8 signal was formed, ZYP1 assembly was severely compromised in all cells of *asy3^aacc^* (*n* = 47 cells in *asy3-1^aacc^* and *n* = 9 in *asy3-2^aacc^*) and some short patches of ZYP1 were detected (Fig. 5, Supplementary Fig. S10), indicating a large failure of synapsis. In contrast, a large portion of cells of the *asy3^aaCC^* and *asy3^AAcc^* showed complete ZYP1 assembly (60.61% in *asy3-1^aaCC^*, *n* = 32 cells and 61.90% in *asy3-2^aaCC^*, *n* = 21; 62.86% in *asy3-1^AAcc^*, *n* = 35% and 65.38% in *asy3-2^AAcc^*, *n* = 26 [Fig. 5, Supplementary Fig. S10]), while the rest of the nuclei harbor only short non-ZYP1 stained chromosome regions (arrowheads in Supplementary Fig. S11). In *asy3^aaCc^* and *asy3^Aacc^* mutants, compared to the REC8-labeled axes, ZYP1 could be installed onto most regions of the chromosomes, although a complete SC assembly was never observed (*n* = 20 cells in *asy3-1^aaCc^* and *n* = 21 in *asy3-2^aaCc^*; *n* = 31 in *asy3-1^Aacc^* and *n* = 26 in *asy3-2^Aacc^*) [arrowheads in Fig. 5, Supplementary Fig. S10]). These results indicate that the presence of only one functional allele of *ASY3* from either A or C sub-genome in *Brassica napus* largely recovers the pairing and synapsis defects induced by the absence of ASY3.

### ASY3 shows dosage-dependent different effects on chiasma formation

To ensure balanced chromosome segregation, each pair of homologs needs to have at least 1 CO, visible as a chiasma in chromosome spreads. In the wild type, in total 19 condensed bivalents were counted in all observed meiocytes of *Brassica napus* at diakinesis (*n* = 48 cells), which were aligned at the equatorial plate at metaphase I to achieve balanced segregation (*n* = 10 cells; Fig. 4A). However, in *asy3^aacc^* null mutants, a large amount of univalents were visible in all nuclei at diakinesis (average 26.90 ± 2.78 univalents, *n* = 100 cells in *asy3-1^aacc^* and 27.14 ± 3.24 univalents, *n* = 42 cells in *asy3-2^aacc^*) and could not move properly to the metaphase I plate in all observed cells (*n* = 24 cells in *asy3-1^aacc^*, and *n* = 20 cells in *asy3-2^aacc^* [Fig. 4A, Supplementary Fig. S8A]). Consequently, chromosome segregation is unbalanced at anaphase I, accompanied by the appearance of chromosome bridges, presumably reflecting the premature separation of sister chromatids (Fig. 4A, Supplementary Fig. S8A). These results support the conclusion that ASY3 is needed for CO formation (Yuan et al. 2009; Wang et al. 2011; Ferdous et al. 2012).

Compared to *asy3^aacc^* mutants, the bivalent formation is largely achieved in the genotypes of *asy3^aaCC^*, *asy3^AAcc^*, *asy3^aaCc^*, and *asy3^Aacc^* plants (Fig. 4A, Supplementary Fig. S8A, Supplementary Table S1). Occasionally, 2 univalents were observed, i.e. 1 pair of chromosomes failed to form a CO (7.5% in *asy3-1^aaCC^*, *n* = 40 cells and 6.67% in *asy3-2^aaCC^*, *n* = 15; 7.41% in *asy3-1^AAcc^*, *n* = 27% and 4.76% in *asy3-2^AAcc^*, *n* = 21; 15.63% in *asy3-1^aaCc^*, *n* = 64% and 14.29% in *asy3-2^aaCc^*, *n* = 56; 13.64% in *asy3-1^Aacc^*, *n* = 66% and 17.46% in *asy3-2^Aacc^*, *n* = 63) (arrowheads in Supplementary Fig. S8C). However, more than 2 univalents were never detected in *asy3^aaCC^*, *asy3^AAcc^*, *asy3^aaCc^*, and *asy3^Aacc^* plants.

Next, we quantified the chiasma number based on the chromosome configurations at diakinesis/metaphase I: ring-type bivalent has at least 2 chiasmata while rod-type bivalent has 1 chiasma (Fig. 4B, Supplementary Table S1). Notably, this estimated number of chiasmata is possibly lower than the actual number of COs, especially in meiotic mutants. In the wild type, on average 31.53 ± 1.56 chiasmata (*n* = 32 cells) are formed, which contains 65.95% ring-type and 34.05% rod-type bivalents (Fig. 4, B and C; *n* = 32 cells). In *asy3^aacc^* mutants, 70.79% (*n* = 100 cells) and 71.43% (*n* = 42 cells) of chromosome pairs occurred as univalents in *asy3-1^aacc^* (6.04 ± 1.71 chiasmata, *n* = 100 cells) and *asy3-2^aacc^* (5.83 ± 1.73 chiasmata, *n* = 42 cells) mutants, respectively, with the remaining bivalents present mainly as the rod type (Fig. 4, Supplementary Fig. S8A). This defect of chiasma formation was almost fully recovered in *asy3^aaCC^* and *asy3^AAcc^* mutants in which the number of chiasmata reached wild-type level (32.33 ± 1.66 chiasmata in *asy3-1^aaCC^*, *n* = 40 cells and 31.80 ± 1.49 in *asy3-2^aaCC^*, *n* = 44; 31.56 ± 2.01 in *asy3-1^AAcc^*, *n* = 54 and 32.21 ± 1.58 in *asy3-2^AAcc^*, *n* = 43 vs 31.53 ± 1.56 in wild type, *n* = 32 [Fig. 4, A and C, Supplementary Figure S8A]), despite a fraction of cells harbors 1 pair of univalents (7.5% in *asy3-1^aaCC^*, *n* = 40 cells and 6.67% in *asy3-2^aaCC^*, *n* = 15; 7.41% in *asy3-1^AAcc^*, *n* = 27% and 4.76% in *asy3-2^AAcc^*, *n* = 21 [see above]).

Unexpectedly, we found that the ratio of ring-type bivalents in both *asy3^aaCc^* and *asy3^Aacc^* mutants is dramatically increased compared to wild type (78.33% in *asy3-1^aaCc^*, *n* = 51% and 76.91% in *asy3-2^aaCc^*, *n* = 44; 80.54% in *asy3-1^Aacc^*, *n* = 53 cells and 80.66% in *asy3-2^Aacc^*, *n* = 40 vs 65.95% in wild type, *n* = 32), indicating a significant increase of the total number of chiasmata (33.76 ± 1.71 in *asy3-1^aaCc^*, *n* = 51 and 33.43 ± 1.81 in *asy3-2^aaCc^*, *n* = 44; 34.15 ± 1.77 in *asy3-1^Aacc^*, *n* = 53 cells and 34.18 ± 1.67 in *asy3-2^Aacc^*, *n* = 40 vs 31.53 ± 1.56 in wild type, *n* = 32, Game–Howell's multiple comparisons test, *P* < 0.001 [Fig. 4, B and C, Supplementary Table S1]).

### ASY3 dosage-dependent effects on DSB formation

Meiotic DSB formation is a prerequisite for chromosome synapsis and recombination. Previous studies show that DSB formation is reduced in *asy3* mutants of Arabidopsis (Ferdous et al. 2012). To study the effects of different ASY3 dosages on DSB formation in *Brassica napus*, we sought to examine the chromosomal loading of the meiosis-specific recombinase DMC1 that relies strictly on the level of DSBs and thus that has been used as an indirect way to infer the situation of DSB formation. To this end, we generated an antibody that specifically recognizes DMC1 (Supplementary Fig. S4B). The immunostaining shows that DMC1 forms extensive foci along chromosomes at early prophase I in the wild type of *Brassica napus*. However, while numerous DMC1 foci were also detected in *asy3^aacc^*, we noticed a clear decrease in their signal intensity (Supplementary Fig. 12A). Considering that it is not possible to count unambiguously the number of DMC1 foci in *Brassica napus*, we quantified their signal intensity for the entire nucleus. This quantification confirms that compared to the wild type (*n* = 19 cells), the DMC1 signal is moderately yet significantly reduced in *asy3^aacc^* mutants (∼ 23% decrease in *asy3-1^aacc^*, *n* = 26 cells) (Supplementary Fig. 12B).

In contrast, the levels of DMC1 signal in *asy3^aaCC^*, *asy3^AAcc^*, *asy3^aaCc^*, and *asy3^Aacc^* (*n* = 20, 42, 15, and 42 cells, respectively) are all similar to that in the wild type (Supplementary Fig. S12), suggesting that the chromosomal loading of DMC1 and DSB formation are not very sensitive to the dosage reduction of ASY3. These results indicate that the formation of DSBs is likely decreased in the absence of ASY3 in *Brassica napus*, which is consistent with previous reports in Arabidopsis *asy3* mutants (Ferdous et al. 2012). Nevertheless, we could not fully exclude that the localization defect of ASY1 in the absence of ASY3 might also contribute to the reduced level of DMC1 since ASY1 seems to be crucial for the recruitment/stabilization of DMC1 in Arabidopsis (Sanchez-Moran et al. 2007).

### ASY3 is required for the wild-type level of initial loading of HEI10

To further understand the molecular effect of a reduction of ASY3 dosage on meiotic recombination, we performed immunolocalization analysis for HEI10 at different stages of male meiosis (Supplementary Table S2). HEI10 (also known as ZHP-3/4 in *C. elegans*), a ring-type E3 ubiquitin ligase, belongs to the ZMM group of proteins. HEI10 displays a dynamic localization pattern during meiotic prophase I which seems conserved in many species including *Brassica napus*, i.e. it initially forms numerous small foci at zygotene and early pachytene that are progressively consolidated into large foci colocalizing with the class I CO sites at late pachytene, diplotene, and diakinesis (Chelysheva et al. 2012; Wang et al. 2012; Grandont et al. 2014; Mercier et al. 2015; Pinzón et al. 2021). The specificity of the HEI10 antibody was validated in the Arabidopsis *hei10-2* mutant (Supplementary Fig. S4C).

**Figure 6.**
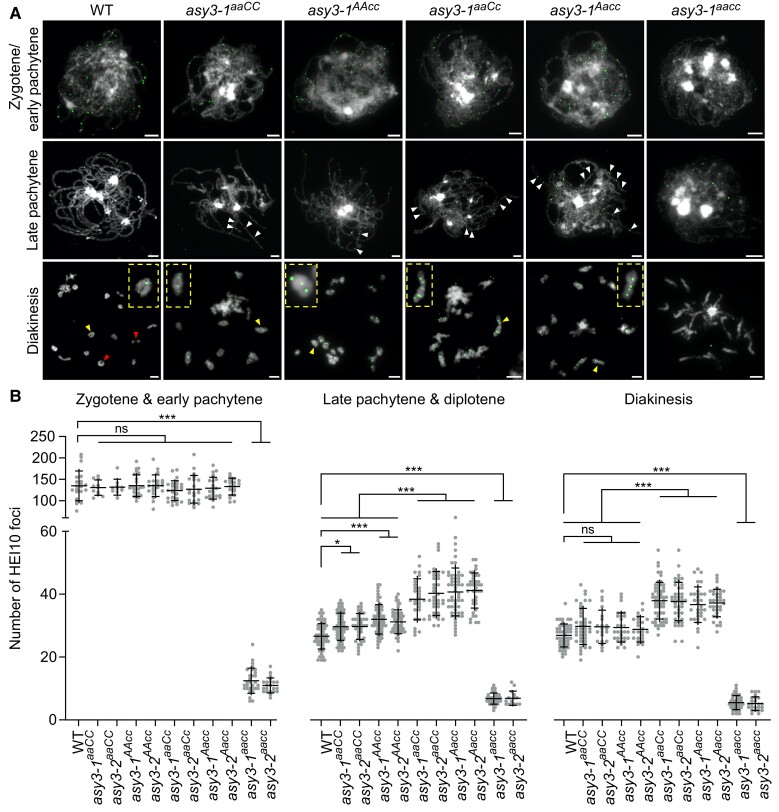
HEI10 localization in wild-type and *asy3* mutants. **A)** Immunostaining of HEI10 at early prophase I (zygotene/early pachytene), late pachytene, and diakinesis in male meiocytes of WT, *asy3-1^aaCC^*, *asy3-1^AAcc^*, *asy3-1^aaCc^*, *asy3-1^Aacc^*, and *asy3-1^aacc^* mutant plants. White arrowheads in the images of late pachytene indicate the closely localized HEI10 foci along 1 paired chromosome. Red arrowheads indicate the position of class II COs. Yellow arrowheads depict the magnified bivalents shown in the yellow rectangles. Bars: 5 *µ*m. **B)** Quantification of the number of HEI10 foci at zygotene/pachytene, late pachytene/diplotene, and diakinesis in male meiocytes of WT (*n* = 21, 61, and 48 cells, respectively), *asy3-1^aaCC^* (*n* = 11, 66, and 41 cells), *asy3-2^aaCC^* (*n* = 10, 39, and 21 cells), *asy3-1^AAcc^* (*n* = 27, 61, and 30 cells), *asy3-2^AAcc^* (*n* = 21, 52, and 24 cells), *asy3-1^aaCc^* (*n* = 26, 29, and 58 cells), *asy3-2^aaCc^* (*n* = 21, 37, and 44 cells), *asy3-1^Aacc^* (*n* = 24, 55, and 33 cells), *asy3-2^Aacc^*(*n* = 20, 39, and 34 cells), *asy3-1^aacc^* (*n* = 30, 41, and 44 cells), and *asy3-2^aacc^* (*n* = 22, 15, and 24 cells) mutant plants. The detailed numbers of cell counted for each stage in each genotype were shown in the relevant main texts. Error bars indicate mean ± SD. Games–Howell's multiple comparisons test, ** *P* < 0.01, and *** *P* < 0.001.

Counting the number of HEI10 foci at zygotene and early pachytene, we found that the numbers of HEI10 foci in asy3*-1^aaCC^* (130.73 ± 17.04, *n* = 11), *asy3-2^aaCC^* (131.60 ± 17.60, *n* = 10), *asy3-1^AAcc^* (135.30 ± 24.71, *n* = 27), *asy3-2^AAcc^* (135.24 ± 24.73, *n* = 21), *asy3-1^aaCc^* (123.77 ± 22.52, *n* = 26), and *asy3-2^aaCc^* (126.81 ± 31.38, *n* = 21), *asy3-1^Aacc^* (129.38 ± 24.76, *n* = 24), *asy3-2^Aacc^* (133.15 ± 19.18, *n* = 20) mutants are not significantly different from that in wild type (134.90 ± 33.88, *n* = 21 [Games–Howell's multiple comparisons test, *P* > 0.05]; Fig. 6, A and B, Supplementary Fig. 13A, Supplementary Table S2), suggesting that the initial HEI10 loading is maintained at least when ASY3 protein amount is reduced to ∼30% to 40% of wild type. However, this number is drastically reduced in *asy3-1^aacc^* (12.43 ± 3.92, *n* = 30) and *asy3-2^aacc^* (10.95 ± 2.33, *n* = 22) mutants (Fig. 6, A and B, Supplementary Fig. 13A). Accordingly, we saw that the initial HEI10 loading is also largely compromised in Arabidopsis *asy3-1* mutants (11.14 ± 4.00 in *asy3-1*, *n* = 21 vs 37.38 ± 8.35 in wild type, *n* = 24 [student's t-test, *P* < 0.001; Supplementary Fig. 13, B and C]). These results suggest that ASY3 is important for the initial recruitment of HEI10 onto chromosomes at early prophase I in both *Brassica napus* and Arabidopsis.

### ASY3 dosage modulates the formation of class i crossovers

Similar to Arabidopsis, at late pachytene and diplotene, the number of HEI10 foci in *asy3-1^aacc^* (6.76 ± 1.75, *n* = 41 cells) and *asy3-2^aacc^* (6.87 ± 2.19, *n* = 15) is strongly reduced compared to that of wild type (26.59 ± 3.99, *n* = 61); at diakinesis, the number of HEI10 foci is decreased from 26.88 ± 3.62 (*n* = 48) in the wild type to 5.48 ± 2.20 (*n* = 44) in *asy3-1^aacc^* and 5.17 ± 2.13 (*n* = 24) in *asy3-2^aacc^* (Games–Howell's multiple comparisons test, *P* < 0.001 [Fig. 6, A and B, Supplementary Fig. 13A, Supplementary Table S2]). This suggests that ASY3 is required for the formation of interference-sensitive COs, consolidating its role in promoting synapsis and chiasma formation.

Unexpectedly, in both *asy3^aaCC^* and *asy3^AAcc^* mutant alleles, the number of HEI10 foci is slightly, yet significantly increased at late pachytene and diplotene (29.65 ± 4.30 in *asy3-1^aaCC^*, *n* = 66 and 29.72 ± 4.06 in *asy3-2^aaCC^*, *n* = 39; 32.02 ± 4.64 in *asy3-1^AAcc^*, *n* = 61 and 31.21 ± 3.75 in *asy3-2^AAcc^*, *n* = 52 vs 26.59 ± 3.99 in wild type, *n* = 61, Games–Howell's multiple comparison tests, *P* < 0.05); at diakinesis, HEI10 foci show a wild-type-like level in both *asy3^aaCC^* (29.76 ± 5.69 in asy3*-1^aaCC^*, *n* = 41 and 29.62 ± 5.16 in *asy3-2^aaCC^*, *n* = 21 vs 26.88 ± 3.62 in wild type, *n* = 48, *P* = 0.20 and 0.55, respectively) and *asy3^AAcc^* (29.40 ± 4.59 in *asy3-1^AAcc^*, *n* = 30 and 28.79 ± 3.95 in *asy3-2^AAcc^*, *n* = 24 vs 26.88 ± 3.62 in wild type, *n* = 48, *P* = 0.32 and 0.68, respectively [Games–Howell's multiple comparisons test; Fig. 6, A and B, Supplementary Fig. 13A]).

More strikingly, we found that the number of HEI10 foci in the mutants having 1 functional *ASY3* allele is even more dramatically elevated at late pachytene/diplotene, reaching 38.38 ± 6.40 in *asy3-1^aaCc^* (*n* = 29), 40.24 ± 6.91 in *asy3-2^aaCc^* (*n* = 37), 40.71 ± 7.54 in *asy3-1^Aacc^* (*n* = 55), and 41.18 ± 5.51 in *asy3-2^Aacc^* (*n* = 39), i.e. 44%, 51%, 53%, and 55% increase in comparison to wild type, respectively (Fig. 6, A and B, Supplementary Fig. 13A). This significant elevation of HEI10 foci in *asy3^Aacc^* and *asy3^aaCc^* is further confirmed in meiocytes at diakinesis (37.91 ± 5.78 in *asy3-1^aaCc^*, *n* = 58 and 37.66 ± 6.03 in *asy3-2^aaCc^*, *n* = 44; 36.67 ± 5.58 in *asy3-1^Aacc^*, *n* = 33 and 37.21 ± 4.30 in *asy3-2^Aacc^*, *n* = 34 vs 26.88 ± 3.62 in wild type, *n* = 48, 41%, 40%, 36%, and 38% increase in comparison to wild type, respectively [Games–Howell's multiple comparison tests, *P* < 0.001; Fig. 6, A and B, Supplementary Fig. 13A]). This result is consistent with the increase of chiasmata in *asy3^aaCc^* and *asy3^Aacc^* mutants (Fig. 4C). Altogether, these results show that ASY3 modulates the formation of class I COs in a dosage-dependent manner.

### Interference-insensitive COs are reduced in *asy3^aacc^*, but not in *asy3^aaCC^*, *asy3^AAcc^*, *asy3^aaCc^*, and *asy3^Aacc^*

To understand whether ASY3 also plays a role in the formation of class II (interference-insensitive) COs, we estimated the amount of class II COs by analyzing the configurations of bivalents labeled by HEI10 at diakinesis; i.e. non-HEI10 labeled chiasmata were treated as class II COs (e.g. red arrowheads in Fig. 6A), since no cytological marker for class II COs is reported so far. Noting that the number of class II COs could be potentially underestimated due to the incapacity to recognize the closely localized chiasmata. In this way, the estimated total amount of COs equals the HEI10 foci on bivalents of diakinesis plus the amount of class II COs (i.e. non-HEI10 labeled chiasmata).

**Figure 7.**
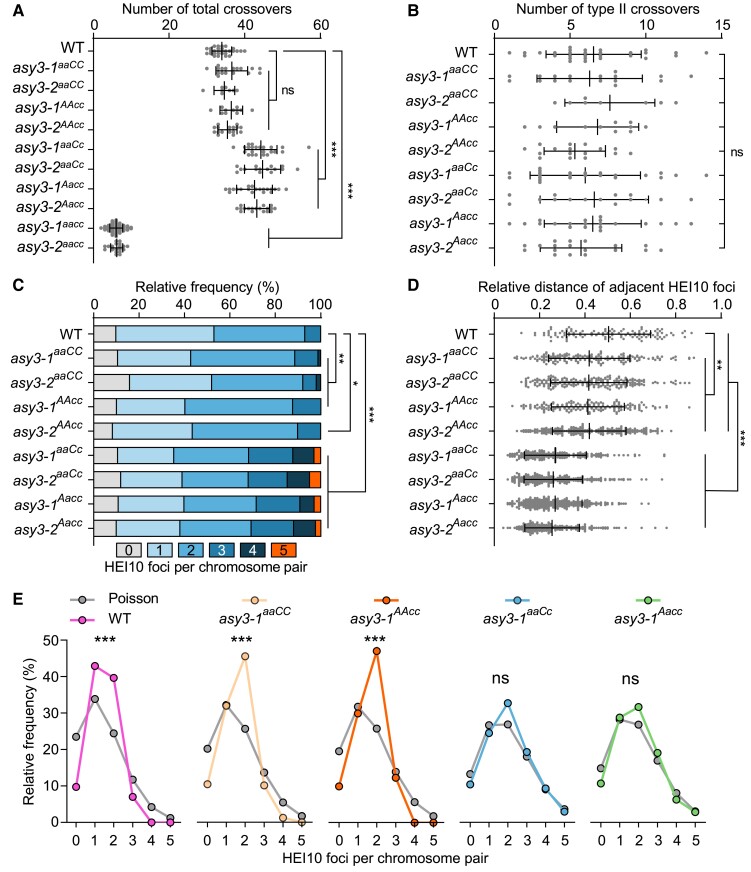
ASY3 dosage modulates crossover number and distribution. **A)** Quantification of the estimated number of total COs in male meiocytes of WT (*n* = 29 cells), *asy3-1^aaCC^* (*n* = 17 cells), *asy3-2^aaCC^* (*n* = 8 cells), *asy3-1^AAcc^* (*n* = 11 cells), *asy3-2^AAcc^* (*n* = 16 cells), *asy3-1^aaCc^* (*n* = 23 cells), *asy3-2^aaCc^* (*n* = 15 cells), *asy3-1^Aacc^* (*n* = 20 cells), and *asy3-2^Aacc^* (*n* = 18 cells) mutant plants. Games–Howell's multiple comparisons test. **B)** Quantification of the estimated amount of Class II COs in male meiocytes of WT (*n* = 29 cells), *asy3-1^aaCC^* (*n* = 17 cells), *asy3-2^aaCC^* (*n* = 8 cells), *asy3-1^AAcc^* (*n* = 11 cells), *asy3-2^AAcc^* (*n* = 16 cells), *asy3-1^aaCc^* (*n* = 23 cells), *asy3-2^aaCc^* (*n* = 15 cells), *asy3-1^Aacc^* (*n* = 20 cells), and *asy3-2^Aacc^* (*n* = 18 cells) mutant plants. Tukey's multiple comparisons test. **C)** Distribution of the number of HEI10 foci on each bivalent at diakinesis in male meiocytes of WT (*n* = 551 bivalents), *asy3-1^aaCC^* (*n* = 323 bivalents), *asy3-2^aaCC^* (*n* = 152 bivalents), *asy3-1^AAcc^* (*n* = 209 bivalents), *asy3-2^AAcc^* (*n* = 304 bivalents), *asy3-1^aaCc^* (*n* = 437 bivalents), *asy3-2^aaCc^* (*n* = 285 bivalents), *asy3-1^Aacc^* (*n* = 380 bivalents), and *asy3-2^Aacc^* (*n* = 342 bivalents) mutant plants. Games-–Howell's multiple comparisons test. **D)** Relative distance of adjacent HEI10 foci on each bivalent at diakinesis in male meiocytes of WT (*n* = 106), *asy3-1^aaCC^* (*n* = 169), *asy3-2^aaCC^* (*n* = 217), *asy3-1^AAcc^* (*n* = 160), *asy3-2^AAcc^* (*n* = 201), *asy3-1^aaCc^* (*n* = 188), *asy3-2^aaCc^* (*n* = 251), *asy3-1^Aacc^* (*n* = 264), and *asy3-2^Aacc^* (*n* = 229) mutant plants. **E)** Comparison of the observed and Poisson-predicted distributions of the number of HEI10 foci per chromosome pair (bivalent) in male meiocytes of WT, *asy3-1^aaCC^*, *asy3-1^AAcc^*, *asy3-1^aaCc^*, and *asy3-1^Aacc^* mutant plants according to (C). Chi-square test. * *P* < 0.05, ** *P* < 0.01, and *** *P* < 0.001. Error bars in **A**, **B** and **D**) indicate mean ± SD.

Based on this estimation, the numbers of class II and total COs in wild type were 6.55 ± 3.09 (*n* = 29) and 33.97 ± 2.54 (*n* = 29), respectively (Fig. 7, A and B). We found that the numbers of all COs and class II COs were not significantly altered in *asy3^aaCC^* and *asy3^AAcc^* mutants (Fig. 7, A and B, Supplementary Table S2). However, besides the drastic decrease of total COs (Fig. 7A), the amount of class II COs was significantly reduced to ∼ 0.56 and ∼ 0.66 in *asy3-1^aacc^* and *asy3-2^aacc^* null mutants, respectively (Supplementary Table S2), suggesting that ASY3 in *Brassica napus* is likely required for the formation of both the interference-sensitive and -insensitive COs, as in Arabidopsis (Ferdous et al. 2012).

Notably, we observed that, despite the marked increase of the class I COs in *asy3^aaCc^* and *asy3^Aacc^* (Fig. 6B), the number of class II COs was not altered compared to wild type (6.00 ± 3.56 in *asy3-1^aaCc^*, *n* = 23 and 6.60 ± 3.46 in *asy3-2^aaCc^*, *n* = 15; 6.50 ± 3.12 in *asy3-1^Aacc^*, *n* = 20 and 5.72 ± 2.62 in *asy3-2^Aacc^*, *n* = 18, Tukey's multiple comparisons test, *P* < 0.05) (Fig. 7B), suggesting that the formation of class II COs is likely not sensitive to a moderate reduction of ASY3 dosage. Therefore, we propose that an appropriate modulation of ASY3 dosage could increase the formation of class I COs without affecting class II COs.

### ASY3 dosage modifies crossover interference

CO interference spaces adjacent COs and leads to the ubiquitous observation that each bivalent typically contains only 1–3 COs (Mercier et al. 2015; Zickler and Kleckner 2015). In wild-type male meiocytes of *Brassica napus*, we observed typically only 1 large HEI10 focal point consolidated along a long stretch of synapsed chromosomes at late pachytene (Fig. 6A), consistent with previous studies in rapeseed and other organisms (Chelysheva et al. 2012; Wang et al. 2012; Grandont et al. 2014; Gonzalo et al. 2019; Morgan et al. 2021; Desjardins et al. 2022). However, the phenomenon of 2 or even more HEI10 foci localized on a short interval of synapsed chromosomes at late pachytene was frequently observed in *asy3^aaCC^* and *asy3^AAcc^* (Fig. 6A). This phenomenon became more obvious in *asy3^aaCc^* and *asy3^Aacc^* alleles where all nuclei (*n* = 29 in *asy3-1^aaCc^* and *n* = 37 in *asy3-2^aaCc^*; *n* = 55 in *asy3-1^Aacc^* and *n* = 39 in *asy3-2^Aacc^*) showed more closely localized HEI10 foci along 1 synapsed/coaligned chromosome pair compared to that in wild type (white arrowheads in Fig. 6A, Supplementary Fig. 13A). These results suggest that CO interference is likely less effective as ASY3 dosage decreases.

Therefore, we analyzed and plotted the CO pattern by counting the number of HEI10 foci per bivalent at diakinesis (Figs 6A and 7C, Supplementary Fig. 13A). In wild type, the majority of bivalents contained 1 (43.19%) or 2 (39.93%) HEI10 foci (*n* = 551 bivalents); the remaining bivalents had either 3 (7.08%) or no HEI10 foci (9.80%; Fig. 7C). In *asy3^aaCC^*, a small proportion of bivalents had 4 HEI10 foci (1.24%, *n* = 323 bivalents in *asy3-1^aaCC^*, 1.97%, *n* = 152 bivalents in *asy3-2^aaCC^*), which was never observed in wild type (*n* = 551 bivalents), while no bivalent with 4 HEI10 foci was found in *asy3^AAcc^* (Fig. 7C). More strikingly, the ratio of bivalents with 3 or more COs (HEI10 foci) was dramatically increased in both *asy3^aaCc^* (31.81% in *asy3-1^aaCc^*, *n* = 437 bivalents and 31.93% in *asy3-2^aaCc^*, *n* = 285 bivalents) and *asy3^Aacc^* (28.42% in *asy3-1^Aacc^*, *n* = 380 bivalents and 30.70% in *asy3-2^Aacc^*, *n* = 342 bivalents) compared to wild type (7.08%; *n* = 551 bivalents), and a portion of bivalents even contained 5 HEI10 foci (2.97% in *asy3-1^aaCc^* and 4.91% in *asy3-2^aaCc^*; 2.89% in *asy3-1^Aacc^* and 2.34% in *asy3-2^Aacc^* [Figs 6A and 7C, Supplementary Fig. 13A]).

Statistically, the number of HEI10 foci per bivalent in *asy3^aaCC^* and *asy3^AAcc^* was significantly distinct from that in the wild type (Chi-square test, *P =* 0.0017 for *asy3-1^aaCC^* and 0.0026 for *asy3-2^aaCC^*; *P =* 0.035 for *asy3-1^AAcc^* and 0.0483 for *asy3-2^AAcc^* [Fig. 7C]). Notably, there was a strongly altered distribution of HEI10 focus number per chromosome pair in both *asy3^aaCc^* and *asy3^Aacc^* compared to the wild-type, *asy3^aaCC^*, and *asy3^AAcc^* plants (Chi-square test, *P <* 0.001 [Fig. 7C]). Moreover, we measured the relative distance of adjacent HEI10 foci at diakinesis (see Materials and Methods). We found that compared to the wild type (0.50 ± 0.18, *n* = 106), this distance became slightly yet significantly closer in *asy3^aaCC^* and *asy3^AAcc^* (0.42 ± 0.18 in *asy3-1^aaCC^*, *n* = 169 and 0.42 ± 0.17 in *asy3-2^aaCC^*, *n* = 217; 0.41 ± 0.16 in *asy3-1^AAcc^*, *n* = 160 and 0.42 ± 0.16 in *asy3-2^AAcc^*, *n* = 201, Games–Howell's multiple comparisons test, *P* < 0.01) and was drastically reduced in *asy3^aaCc^* (0.27 ± 0.14 in *asy3-1^aaCc^*, *n* = 188 and 0.26 ± 0.13 in *asy3-2^aaCc^*, *n* = 251) and *asy3^Aacc^* (0.27 ± 0.12 in *asy3-1^Aacc^*, *n* = 264 and 0.26 ± 0.12 in *asy3-2^Aacc^*, *n* = 229 [Games Game Howell's multiple comparisons test, *P* < 0.001; Fig. 7D), implying further the attenuation of CO interference.

Furthermore, we performed a distribution analysis for HEI10 focus number per bivalent at diakinesis. In the wild type, the distribution of HEI10 focus number per chromosome pair significantly deviated from an expected Poisson random distribution (Chi-square test, wild type vs Poisson, and *P* = 6.48E-31; Fig. 7E). In *asy3^aaCC^* and *asy3^AAcc^*, HEI10 distribution was also significantly distinct from the Poisson distribution (Chi-square test, *asy3-1^aaCC^* vs Poisson, and *P* = 9.95E-17; *asy3-2^aaCC^* vs Poisson, and *P* = 4.28E-5; *asy3-1^AAcc^* vs Poisson, *P* = 2.72E-12; *asy3-2^AAcc^* vs Poisson, and *P* = 1.81E-19 [Fig. 7E, Supplementary Fig. S14]). Considering the shorter distance of inter-HEI10 foci and modified HEI10 distribution on diakinesis bivalents compared to wild type (Fig. 7, C and D), we conclude that CO interference is mildly reduced in *asy3^aaCC^* and *asy3^AAcc^*. Strikingly, the observed HEI10 distribution per bivalent in *asy3^aaCc^* and *asy3^Aacc^* largely fits the expected values of Poisson distribution (Chi-square test, *asy3-1^aaCc^* vs Poisson, *P* = 0.08; *asy3-2^aaCc^* vs Poisson, *P* = 0.78; *asy3-1^Aacc^* vs Poisson, *P* = 0.07; *asy3-2^Aacc^* vs Poisson, *P* = 0.16 [Fig. 7E, Supplementary Fig. S14]), suggesting that CO interference is compromised when ASY3 dosage is reduced to less than half of the wild-type level.

## Discussion

The chromosome axis plays a crucial role in CO patterning by providing a platform for recruiting the recombination proteins and transmitting the force of CO interference (Sanchez-Moran et al. 2007; Zhang et al. 2014b; Zickler and Kleckner 2015; Lambing et al. 2020a,b). Previous work in Arabidopsis has shown that in both *asy3* and *asy1* mutants, CO formation is severely compromised, resulting in a large reduction in bivalent formation (Ross et al. 1997; Moran et al. 2001; Ferdous et al. 2012; Cuacos et al. 2021; Dio et al. 2023). The remaining COs in *asy1* mutants have been shown to be clustered in telomere-proximal regions and exhibit compromised interference, suggesting a role of ASY1 in antagonizing telomere-led recombination and promoting crossover formation in interstitial chromosome arms (Lambing et al. 2020a; Pochon et al. 2022). In Arabidopsis *asy1/+* (∼21% reduction in ASY1 loading) and *asy3/+* (∼25% reduction in ASY1 loading) heterozygous mutants, the CO landscape is also remodeled, with a shift toward the distal subtelomeres, at the expense of interstitial and pericentromeric regions (Lambing et al. 2020a). However, the global CO numbers and interference are still maintained in Arabidopsis *asy1/+* and *asy3/+* heterozygotes with full pairing and synapsis occurring (Lambing et al. 2020a).

### ASY3 has dosage-dependent diverse effects on recombination

**Figure 8.**
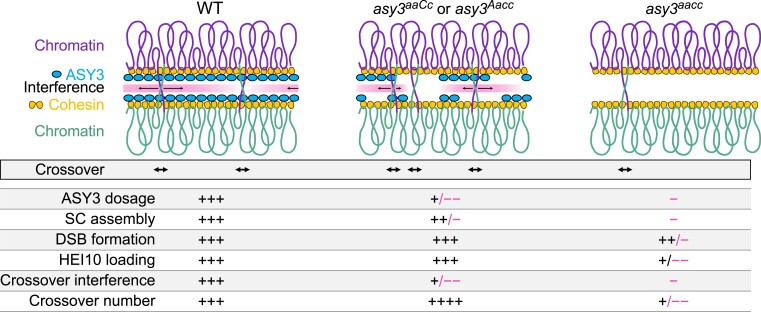
Model for the crossover formation with different ASY3 dosages. In wild type, a high level of ASY3 ensures the fidelity of DSB formation, HEI10 loading, and SC assembly that, on the one hand, promotes the interhomolog recombination and, on the other hand, provides the platform for HEI10 dynamic coarsening, thus implementing both the CO promotion and interference. In the absence of ASY3, SC assembly is disrupted, DSB formation is reduced, and initial HEI10 loading is interfered, which largely compromises both the CO promotion and interference, resulting in the drastic decrease of COs. In *asy3^aaCc^* or *asy3^Aacc^* where ASY3 dosage is reduced to ∼30% to 40% of the wild-type level, CO interference is compromised, whereas DSB formation, HEI10 loading, and SC assembly are still maintained to a large extent, resulting in the global increase of COs.

Here, we make use of the tetraploid nature of *Brassica napus* to generate an allelic series of *asy3* mutants allowing a fine-grained dissection of the dosage–function relationship and address how the axis protein ASY3 modulates CO frequency and distribution (Fig. 8). In the wild type, ASY3 promotes synapsis and ensures that each pair of chromosomes forms the obligatory CO. At the same time, a high/wild-type level of ASY3 facilitates CO interference. Consequently, each pair of chromosomes typically obtains only 1 to 3 COs (Hunter 2015; Mercier et al. 2015). In *asy3^aacc^* mutants where ASY3 is completely absent, CO formation is severely disrupted due to the loss of CO promotion mediated by ASY3, indicating that ASY3 is crucial for CO generation.

Interestingly, our data show that when ASY3 dosage is reduced to ∼30% to 40% of the wild type, as seen in the *asy3^aaCc^* and *asy3^Aacc^* plants, its function in promoting pairing and CO formation is largely maintained (Figs. 4 and 5), but the competence to orchestrate interference is almost abolished (see below), thus leading to an increase of class I COs while only a mild defect in CO assurance occurs (Fig. 8). This could indicate the existence of a threshold over which ASY3 imposes CO interference (directly or indirectly) and, in turn, constrains the formation of excess COs.

### Mechanisms of ASY3 for CO formation

The chromosome axis has been shown to be important for meiotic recombination in many species as diverse as budding yeast, mice, *Drosophila*, *C. elegans*, Arabidopsis, rice, and wheat (Hollingsworth et al. 1990; Hollingsworth and Johnson 1993; Couteau and Zetka 2005; Martinez-Perez and Villeneuve 2005; Goodyer et al. 2008; Sanchez-Moran et al. 2008; Boden et al. 2009; Wojtasz et al. 2009; Joyce and McKim 2010; Latypov et al. 2010; Wang et al. 2011; Dubois et al. 2019; Dio et al. 2023). Based on previous studies and our data, we reason that ASY3 might promote CO formation in at least 2 ways. First, as a conserved structural component of the axis and SC, ASY3 facilitates the interhomolog recombination by promoting pairing and synapsis, e.g. via ensuring the formation of the wild-type level of DSBs and establishing bridges between the recombinational nucleofilament and homolog to promote homology search and strand invasion (Ferdous et al. 2012; Dubois et al. 2019; Ito and Shinohara 2023). This is supported by our data showing that chromosome pairing and synapsis are severely disrupted in *asy3^aacc^* mutants where the axis is likely not formed properly, consistent with previous findings (Figs. 4A, 5 [Ferdous et al. 2012]). Second, we found that the initial loading of HEI10 on chromosomes is severely disrupted in the absence of ASY3 in both Arabidopsis and *Brassica napus* (Fig. 6, Supplementary Fig. 13, B and C). Thus, ASY3 supports the CO formation by ensuring the fidelity of HEI10 loading. Whether other pro-CO factors are also affected by ASY3 remains to be explored.

The question of to which extent the effect of ASY3 dosage reduction on recombination is attributed to its role in ASY1 recruitment remains unclear. Recently, the function of ASY1 was analyzed using a series of tetraploid wheat *asy1* mutants (Dio et al. 2023). In contrast to the series of *asy3* allelic mutants reported here, those *asy1* mutants in tetraploid wheat, display a linear reduction in chiasmata (COs) concomitantly with the decrease of *ASY1* gene dosage, resulting in the failure to maintain CO assurance (Dio et al. 2023). In those wheat mutants with only 1 functional allele of ASY1, distal COs prefer to be formed at the expense of proximal and interstitial COs, supporting the conclusion that ASY1 functions to promote CO formation away from the chromosome ends (Lambing et al. 2020a; Dio et al. 2023). Our data indicate that ASY3 may be an ideal target for manipulation to aim for the global increase of CO frequency in crop breeding without compromising the proximal and interstitial COs. Compared to the *asy1* mutants, one advantage of modulating ASY3 dosage might be that the loading of ASY1 on the centromeric and pericentromeric regions is not affected by the reduction/loss of ASY3, at least in *Brassica napus*, which is important for antagonizing the telomere-led recombination, thus distributing COs more evenly along the chromosomes (Lambing et al. 2020a).

### Role of ASY3 in HEI10 coarsening and CO interference

Recently, a mechanistic coarsening model that quantitatively explains the class I CO patterning was proposed (Morgan et al. 2021; Zhang et al. 2021; Durand et al. 2022; Fozard et al. 2023). According to this model, the SC plays a critical role in controlling and constraining the dynamic coarsening of HEI10 molecules, thus imposing CO interference. This idea is compatible with the finding that the number of HEI10 foci gets increased in the absence of the transverse filament protein of the SC, ZYP1, where interference is abolished, indicating a role of ZYP1/SC in HEI10 dynamics (Capilla-Pérez et al. 2021; France et al. 2021). When ASY3 is absent, the initial loading of HEI10 on chromosomes is compromised, resulting in the deficiency of HEI10 and thus reduced CO formation. Notably, HEI10 initial loading appears normal when ASY3 is reduced by ∼ 60% to 70% in *asy3^aaCc^* and *asy3^Aacc^*. However, this reduction of ASY3 dosage in *asy3^aaCc^* and *asy3^Aacc^* leads to a patchier and less continuous assembly of the SC, which likely causes the HEI10 coarsening to work locally but not along the entire chromosome length, thus compromising the CO interference and increasing CO formation (France et al. 2021). We hence propose that an intact and continuous tripartite structure of the SC is essential for proper HEI10 diffusion and CO interference. In this context, one interesting question is whether the CO formation could be further unleashed when combining the manipulation of ASY3/axis and ZYP1.

In conclusion, the results we present here unravel the dosage-dependent diverse effects of ASY3 on CO formation and provide insights into the role of chromosome axis/SC in CO patterning. Since ASY3 is widely present in a variety of crop species, this work provides a promising target—alone and in combination—for modifying the CO efficiency for crop breeding.

## Materials and methods

### Plant materials and growth conditions

The spring-type *Brassica napus* cultivar *Westar* was used as a wild-type reference throughout this research. Mutants of *Bnaasy3* were generated by the CRISPR/Cas9 gene editing technique in the background of *Brassica napus* cv. *Westar*. The Arabidopsis T-DNA insertion mutants *asy1-4* (SALK_046272), *dmc1-2* (SAIL_170_F08), and *hei10-2* (SALK_014624) were previously described (Crismani et al. 2013; Yang et al. 2020b) and used for validating the specificity of ASY1 and HEI10 antibodies used in this study. Arabidopsis plants were grown in growth chambers under a cycle of 16 h of light (150 *μ*mol photons m^−2^ s^−2^) at 21 °C and 8 h of dark at 18 °C. Rapeseed plants were planted in the experimental fields with normal growing conditions at Huazhong Agricultural University, Wuhan, China.

### Plasmid construction

To generate *Bnaasy3* mutants, 2 sgRNAs (Fig. 1A) were designed to target the first exon of all *ASY3* alleles by CRISPR-P2.0 (http://crispr.hzau.edu.cn/CRISPR2) and then inserted into the binary vector pKSE401 containing the guide RNA and Cas9 expression cassettes via golden gate assembly (Xing et al. 2014). For the yeast 2-hybrid assay of BnaASY1 with BnaASY3, BnaA07.ASY1^1–300aa^-BD, BnaA05.ASY3^FL^-AD, BnaA05.ASY3^1–32aa^-AD constructs were generated. The CDS sequences of *BnaA07.ASY1* (1-300 aa) and *BnaA05.ASY3* (full length and 1-32aa) were amplified by PCR with primers flanked by attB recombination sites (BnaASY1-A07-attB1F and BnaASY1-A07-300aa-attB2R, BnaASY3-A05-attB1F and BnaASY3-A05-attB2R, BnaASY3-A05-attB1F and BnaASY3-A05-32aa-attB2R) and subcloned into *pDONR223* vector by Gateway BP reactions. All these entry clones were subsequently integrated into the destination vectors *pGADT7-GW* or *pGBKT7-GW* vectors by Gateway LR reactions. For split-luciferase complementation assays, the CDS sequences of BnaA07.ASY1^1–300aa^, BnaA05.ASY3 (full length), BnaA05.ASY3 (full length), and cLUC-BnaA05.ASY3^1–32aa^ were amplified by PCR and inserted into the *pJW771*-nLUC or *pJW772-cLUC* vectors. Primers used are listed in Supplementary Table S3.

### Plant transformation and genotyping

The procedure of agrobacterium-mediated transformation of *Brassica napus* was carried out as previously described using hypocotyl explants (Dai et al. 2020). To genotype the CRISPR/Cas9-induced mutations of *ASY3* genes, DNA sequences covering the targeting regions were amplified by PCR using gene-specific primers for *BnaA05.ASY3* and *BnaC04.ASY3* alleles (BnaASY3-A05-F1 and BnaASY3-R1, BnaASY3-C04-F1 and BnaASY3-R1) and were subjected to sequencing. Primers used are listed in Supplementary Table S3.

### RNA extraction and RT-qPCR

Total RNA from the anthers at the meiotic stage was extracted using the RNA Trizol according to the manufacturer's instructions (Invitrogen); 1 *μ*g total RNA was used to synthesize the first-strand cDNA with the RevertAid RT kit (Thermofisher). The reverse transcription quantitative PCR (RT-qPCR) was performed with the real-time PCR system (BioRad 384 wells) using the SYBR Green Master Mix (ABclonal) according to the manufacturer's instructions. Relative expression levels of *BnaA05.ASY3* and *BnaC04.ASY3* were calculated according to the Ct values. The qPCR primers are listed in Supplementary Table S3.

### Yeast two-hybrid assay

For the yeast 2-hybrid assay, the relevant combinations of constructs were co-transformed into the auxotrophic yeast (*Saccharomyces cerevisiae*) strain AH109 using the polyethylene glycol/lithium acetate method according to the manufacturer's manual (Clontech). Yeast cells were dotted on the plates of double (–Leu–Trp) and quadruple (–Leu–Trp–His–Ade) synthetic dropout medium, and images were captured after 3 d of incubation at 28 °C.

### Split-luciferase complementation assay

For split-luciferase complementation assays, the constructs of nLUC-BnaA07.ASY1^1–300aa^, cLUC-BnaA05.ASY3 (full length), and cLUC-BnaA05.ASY3^1–32aa^ were transformed into the agrobacterium GV3101 stain. The agrobacteria were grown at 28 °C and harvested at OD600 ≈ 0.8. The pellets were resuspended in the infiltration buffer (10 mm MES, 10 mm MgCl_2_, and 150 mm acetosyringone). The relevant combinations of agrobacteria were injected into *Nicotiana benthamiana* leaves that were kept in the dark for 12 h and then put back to the normal growth condition for 36 h. To evaluate the interaction, all leaves were injected with 0.3 mg/mL D-luciferin, and then, the luciferase signals were captured using the NightSHADE L985.

### Antibody generation

The polyclonal antibodies against Brassica ASY1, ASY3, REC8, ZYP1, DMC1, and CENH3 were produced by DIA-AN, Wuhan, China (https://www.dia-an.com). Briefly, the coding regions of BnaC06.ASY1 (301-614 aa), BnaC04.ASY3 (481-696 aa), BnaA10.REC8 (377-701 aa), BnaA07.ZYP1A (1-349 aa), BnaC01.DMC1 (1-254 aa), and BnaA09.CENH3 (full length) were amplified and inserted into the pET-32a vector. The corresponding recombinant proteins were produced and purified from *Escherichia coli* bacteria, which were used as antigens to immunize rabbits or rats. After 3-time immunization, antibodies were purified from the antisera using antigen-based affinity purification. The polyclonal antibody against HEI10 was generated by GL Biochem, Shanghai, China (www.glschina.com) using the synthetic peptide (Cys-PANNFYPRHQEP) conjugated to the carrier protein KLH as the antigen.

### Cytological analysis

Pollen viability was performed by dipping the open flowers into the Peterson staining solution (Peterson et al. 2010). For tetrad analysis, flower buds at appropriate size were dissected, and the resulting anthers were treated in 1 mol/L HCl for 1 min at 60 °C. Subsequently, anthers were rinsed in distilled water and squashed in cabol fuchsin solution. Images were captured using a SOPTOP ex30 light microscope equipped with a color camera.

Meiotic chromosome spread analysis was performed as described previously with minor modifications (Chelysheva et al. 2010). Briefly, fresh flower buds were fixed in the ethanol: acetic acid (3:1) fixative for 24 to 48 h at 4 °C, then washed twice with the same fixative, and stored at −20℃. For chromosome spreading, flower buds at appropriate sizes were dissected. Next, anthers were digested in the digestion mix (3% (w/v) cellulase, 3% (w/v) macerozyme, and 5% (w/v) snailase in 50 mm citrate buffer, pH 4.5) for 50 min at 37℃. Then, a single digested anther was mashed into a fine suspension by a hook in 5 *μ*l water on the microscopy slide. Subsequently, 30 *μ*l of 60% (v/v) acetic acid was added to the slide, followed by a gentle stirring without toughing the slide surface using a straight needle for 2 min on a 45 °C hotplate. Finally, before the drop dried out, the cells were fixed on the slide by rinsing the slide with cold fixative (ethanol: acetic acid, 3:1) and air-drying the preparation. Chromosomes were stained with 4′,6-diamidino-2-phenylindole (DAPI) and observed under the fluorescent microscope equipped with a monochrome camera.

For immunostaining, following the preparation of chromosome spreads, the slides were put into a glass jar filled with 10 mm citrate buffer pH 6.0, microwaved until slight boiling, and then transferred immediately into 1 × phosphate-buffered saline with Triton X-100 detergent (PBST) solution (0.1% (v/v) Triton X-100). Next, the slides were first blocked with goat serum for 1–2 h at 28℃ and then incubated with relevant antibodies for 48 h at 4℃. After 3 times washing (5 mins each) in PBST, the slides were incubated with fluorescein-conjugated secondary antibodies (ThermoFisher) for 24 h at 4 °C. Finally, the slides were washed twice (5 mins each) with PBST and stained by DAPI. Images were captured using the SOPTOP RX50 fluorescent microscope (Sunny Optical Technology, China) equipped with a monochrome camera. For the super-resolution imaging of ZYP1 with REC8/ASY3 shown in Supplementary Fig. S9 using STED, Abberior STAR Red (goat anti-Rabbit) and STAR Orange (goat anti-Rat) were used as the secondary antibodies, and images were captured with an Olympus IX83 fluorescent microscope equipped with an Abberior instrument STEDYCON using the 561-nm (for STAR Orange) and 640-nm (for STAR Orange) excitation lasers and a 775-nm STED depletion laser.

### Quantification of the fluorescent intensity and relative distance of HEI10 foci

To quantify the signal intensity of ASY3 and ASY1, 3 to 5 slides were prepared in the same batch of experiment for each genotype using flower buds from different plants, which were then treated in parallel under the same conditions to minimize the slide-to-slide variation, e.g. the same amount and duration of antibody incubation followed by same strength of washing steps. All images were captured under the same exposure conditions; 10 to 15 cells were recorded from each slide. The signal intensity was measured by Fiji. The background signal was subtracted. All raw values were normalized through dividing by the mean signal intensity value of the wild type. Noting that for the quantification of ASY1 signal intensity, the “blob”-like overexposed regions (blue arrowheads in Fig. 2, Supplementary Fig. S6A) were cut out. For the quantification of the “blob”-like ASY1 signal at the brightly DAPI-staining regions, images were captured under short exposure time to make sure that the interested regions were not overexposed (Supplementary Fig. S5B).

For the quantification of the relative distance of adjacent HEI10 foci on each bivalent, the distance between 2 adjacent HEI10 foci on a single bivalent was measured using Fiji and then normalized by the total length of the bivalent.

## Statistical analysis

The 1-way ANOVA followed by Game–Howell's or Tukey's multiple comparisons test and Students' *t*-test were performed using GraphPad Prism 8.0.2 software. For multiple comparisons test, the Tukey's multiple comparison test is applied when the variances of different groups of data are equal (by F-test), and otherwise, the Game–Howell's multiple comparisons test is used. The calculation of the mean and standard deviation, the Poisson distribution comparison, and the Chi-square test were done using Microsoft Excel. All original data used for statistical analyses in this research are shown in Supplementary Data Set.

### Accession numbers

Sequence data from this article can be found in the *Brassica napus* multi-omics information resource database BnIR under the following accession numbers: *BnaA05.ASY3* (BnaA05g00870D), *BnaC04.ASY3* (BnaC04g00500D), *BnaC06.ASY1* (BnaC06g28450D), *BnaA10.REC8* (BnaA10g24970D), *BnaA07.ZYP1A* (BnaA07g20670D), *BnaC01.DMC1* (BnaC01G0367500ZS), and *BnaA09.CENH3* (BnaA09G0694800ZS).

## Supplementary Material

koae207_Supplementary_Data

## Supplementary data

The following materials are available in the online version of this article.

**Supplementary Figure S1.** Analysis of ASY3 in *Brassica napus* (supports Fig. 1).

**Supplementary Figure S2.** Analysis of ASY3 localization in *asy3^aaCC^*, *asy3^AAaa^*, *asy3^aaCc^*, *asy3^Aacc^*, and *asy3^aacc^* mutants (supports Fig. 1).

**Supplementary Figure S3.** Interaction between ASY1 and ASY3 in *Brassica napus* (supports Fig. 2).

**Supplementary Figure S4.** Specificity validation of ASY1, DMC1, and HEI10 antibodies used in this study (supports Figs. 2, 6 and Supplementary Fig. S12).

**Supplementary Figure S5.** Localization of ASY1 at the brightly DAPI-stained heterochromatic regions is independent of ASY3 (supports Fig. 2).

**Supplementary Figure S6.** Localization of ASY1 in *asy3* mutants (supports Fig. 2).

**Supplementary Figure S7.** Phenotypic analysis of *asy3* mutants (supports Fig. 3).

**Supplementary Figure S8.** Analysis of meiotic chromosome behaviors in *asy3* mutants (supports Fig. 4).

**Supplementary Figure S9.** Super-resolution imaging of chromosome synapsis in male meiocytes of wild type using STED (supports Fig. 5).

**Supplementary Figure S10.** ASY3 dosage-dependent effects on chromosome synapsis (supports Fig. 5).

**Supplementary Figure S11.** Representative images of co-immunostaining of ZYP1 and REC8 show cells harboring some non-ZYP1 labeled regions in male meiocytes of *asy3-1^aaCC^*, *asy3-2^aaCC^*, *asy3-1^AAcc^*, and *asy3-2^AAcc^* at pachytene (supports Fig. 5).

**Supplementary Figure S12.** Analysis of DMC1 localization in wild type and *asy3* mutants (supports Fig. 4).

**Supplementary Figure S13.** Analysis of HEI10 localization in *asy3* mutants of Arabidopsis and *Brassica napus* (supports Fig. 6).

**Supplementary Figure S14.** Comparison of the observed and Poisson-predicted distributions of the number of HEI10 foci per chromosome pair (bivalent) in male meiocytes of *asy3-2^aaCC^*, *asy3-2^AAcc^*, *asy3-2^aaCc^*, and *asy3-2^Aacc^* mutant plants (supports Fig. 7).

**Supplementary Table S1.** Summary of chromosome configurations at diakinesis/metaphase I in male meiocytes of wild-type and *asy3* mutants.

**Supplementary Table S2.** Summary of the number of HEI10 foci, total number of COs, and number of type II COs in male meiocytes of wild-type and *asy3* mutants.

**Supplementary Table S3.** Primers used in this research.
