# Supplementary material for: ASYNAPSIS3 has diverse dosage-dependent effects on meiotic crossover formation in Brassica napus
Source: Plant Cell. 2024 Jul 24;36(9):3838–56. doi: 10.1093/plcell/koae207 (PMC11371185; doi:10.1093/plcell/koae207)
Supplement: koae207_Supplementary_Data [file koae207_supplementary_data.zip › Chu et al_Supplementary figures and tables.pdf]

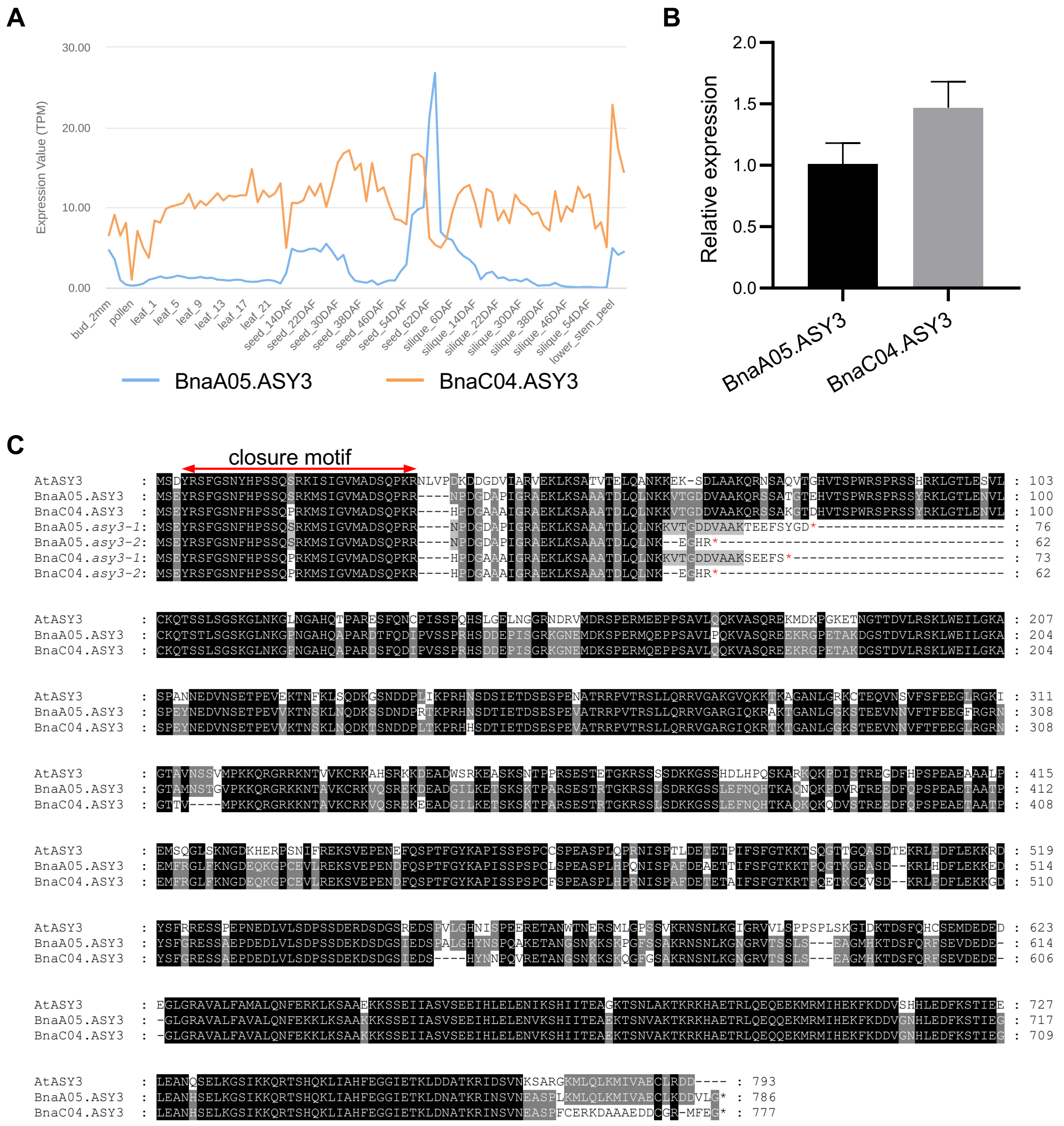

Supplementary Figure S1. Analysis of ASY3 in *Brassica napus* (Supports Figure 1). (A) The TPM (transcripts per million) expression values of *BnaA05.ASY3* and *BnaC04.ASY3* from the transcriptome analyses in different tissues including flower bud, leaf, seed, and silique. (B) Relative expression of *BnaA05.ASY3* and *BnaC04.ASY3* in anthers at meiotic stage using the RT-qPCR. Error bars indicate mean ± SD. (C) Alignment of ASY3 proteins from Arabidopsis and *Brassica napus*. Red asterisks indicate the putative translational stops of mutated ASY3 proteins. The region of the closure motif is highlighted by the red line with double arrowheads.

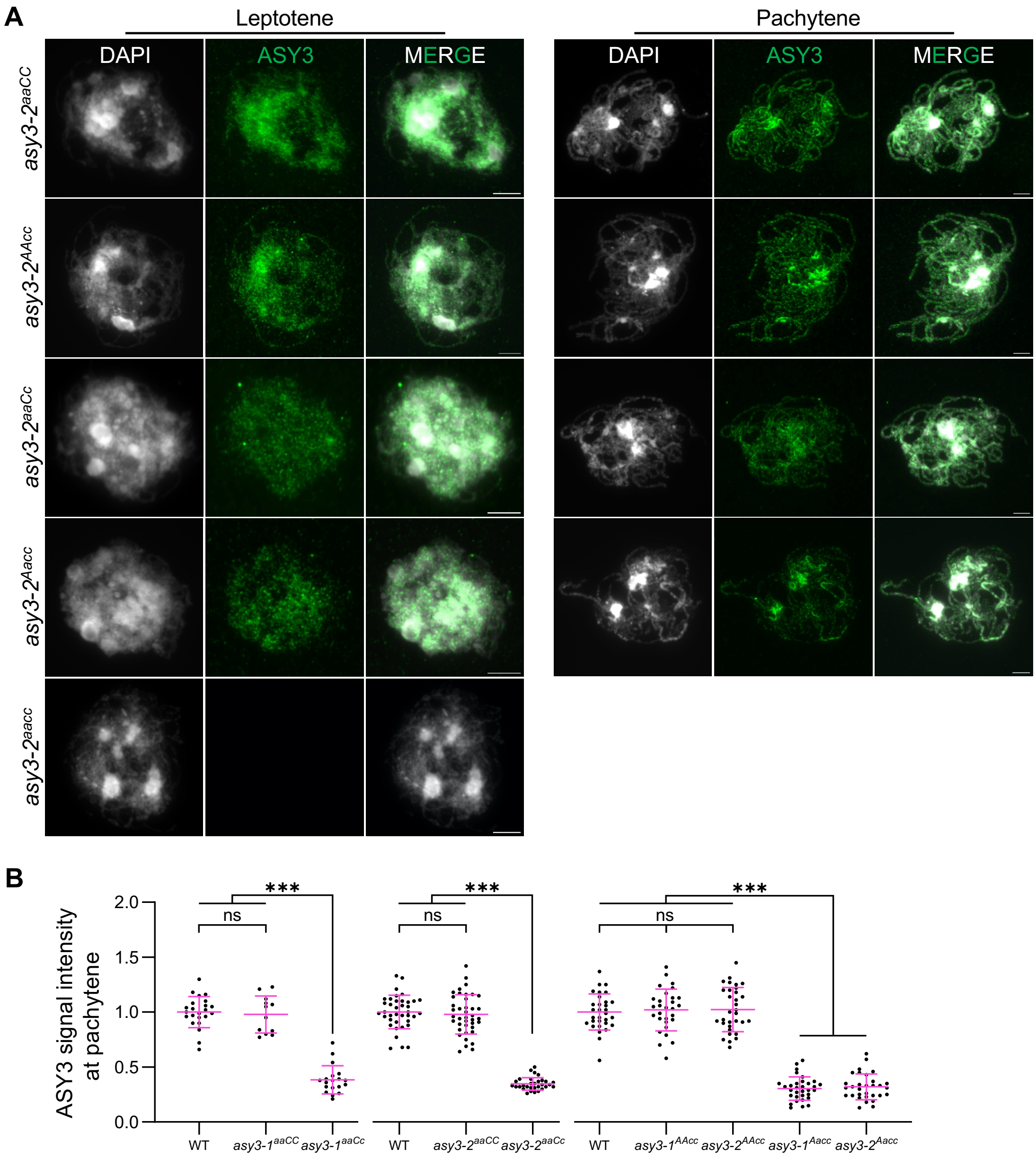

Supplementary Figure S2. Analysis of ASY3 localization in *asy3^{aaCC}*, *asy3^{AAaa}*, *asy3^{aaCc}*, *asy3^{Aacc}* and *asy3^{aacc}* mutants (Supports Figure 1). (A) Immunolocalization of ASY3 in male meiocytes of *asy3-2^{aaCC}*, *asy3-2^{AAaa}*, *asy3-2^{aaCc}*, *asy3-2^{Aacc}* and *asy3-2^{aacc}* mutants at leptotene and pachytene (or -like). Bars: 5µm. (B) Quantification of relative ASY3 signal intensity at pachytene in WT, *asy3-1^{aaCC}*, *asy3-2^{aaCC}*, *asy3-1^{AAcc}*, *asy3-2^{AAcc}*, *asy3-1^{aaCc}*, *asy3-2^{aaCc}*, *asy3-1^{Aacc}*, and *asy3-2^{Aacc}* mutants. Error bars indicate mean ± SD. Asterisks indicate significant difference (Game-Howell's multiple comparisons test, p<0.001).

A

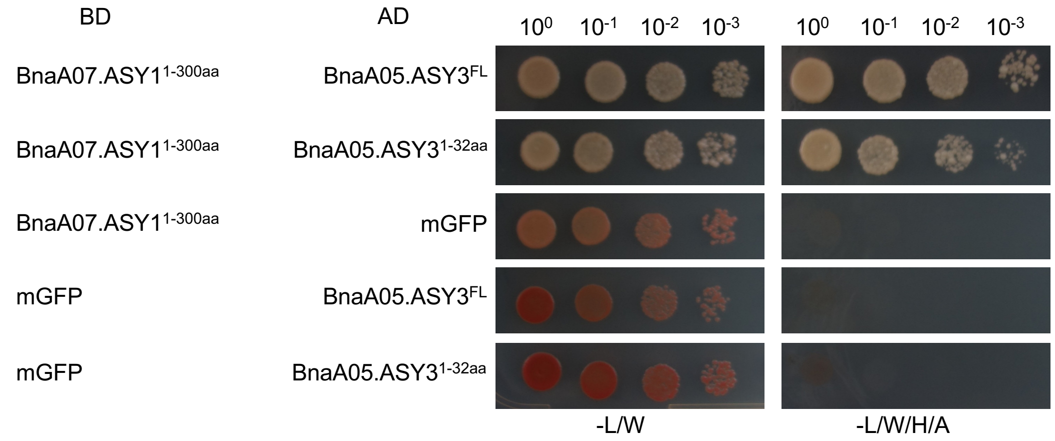

B

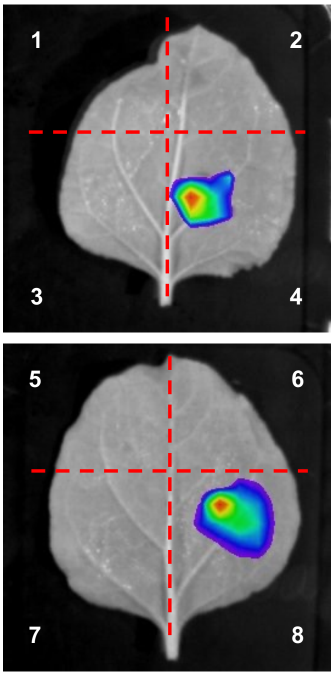

	n-luc	c-luc
1	Empty	Empty
2	BnaA07.ASY1$^{1\text{-}300aa}$	Empty
3	Empty	BnaA05.ASY3^{FL}
4	BnaA07.ASY1$^{1\text{-}300aa}$	BnaA05.ASY3^{FL}
5	Empty	Empty
6	BnaA07.ASY1$^{1\text{-}300aa}$	Empty
7	Empty	BnaA05.ASY3$^{1\text{-}32aa}$
8	BnaA07.ASY1$^{1\text{-}300aa}$	BnaA05.ASY3$^{1\text{-}32aa}$

Supplementary Figure 3. Interaction between ASY1 and ASY3 in *Brassica napus* (Supports Figure 2). (A) Yeast two-hybrid assay testing for interaction of BnaA07.ASY1 HORMA domain (1-300 aa) with BnaA05.ASY3 full length (FL) and the closure motif (1-32aa). Yeast cells harbouring both the AD and BD plasmids were grown on synthetic dropout (SD) medium in the absence of Leu and Trp (-L/W, left panel) and of Leu, Trp, His, and Ade (-L/W/H/A, right panel). Yeast cells were incubated until OD_{600}=1 and then diluted 10-, 100-, and 1,000-fold for the assay. (B) Split-luciferase complementation assay for the interaction of BnaA07.ASY1 HORMA domain (1-300 aa) with BnaA05.ASY3 full length (FL) and the closure motif (1-32aa).

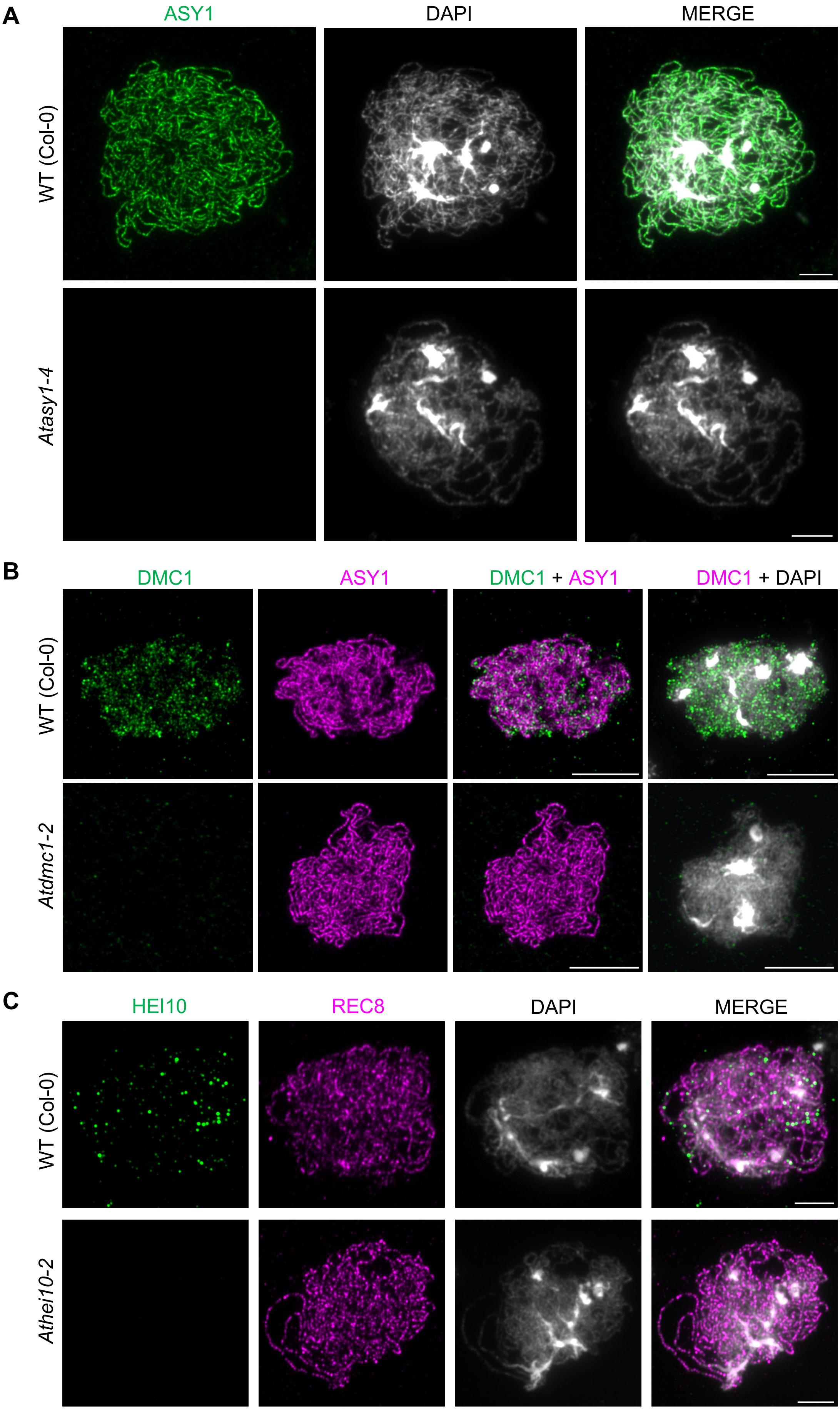
A
ASY1
DAPI
MERGE
WT (Col-0)
Atasy1-4
B
DMC1
ASY1
DMC1 + ASY1
DMC1 + DAPI
WT (Col-0)
Atdmc1-2
C
HEI10
REC8
DAPI
MERGE
WT (Col-0)
Athei10-2

Supplementary Figure S4. Specificity validation of ASY1, DMC1, and HEI10 antibodies used in this study (Supports Figure 2, 6, and S12). (A) Immunostaining of ASY1 in male meiocytes of Arabidopsis *asy1-4* mutant at early prophase I. (B) Co-immunostaining of DMC1 and ASY1 in male meiocytes of Arabidopsis *dmc1-2* mutant at early prophase I. (C) Co-immunostaining of HEI10 and REC8 in male meiocytes of Arabidopsis *hei10-2* mutant at early prophase I. Bars: 5 μm.

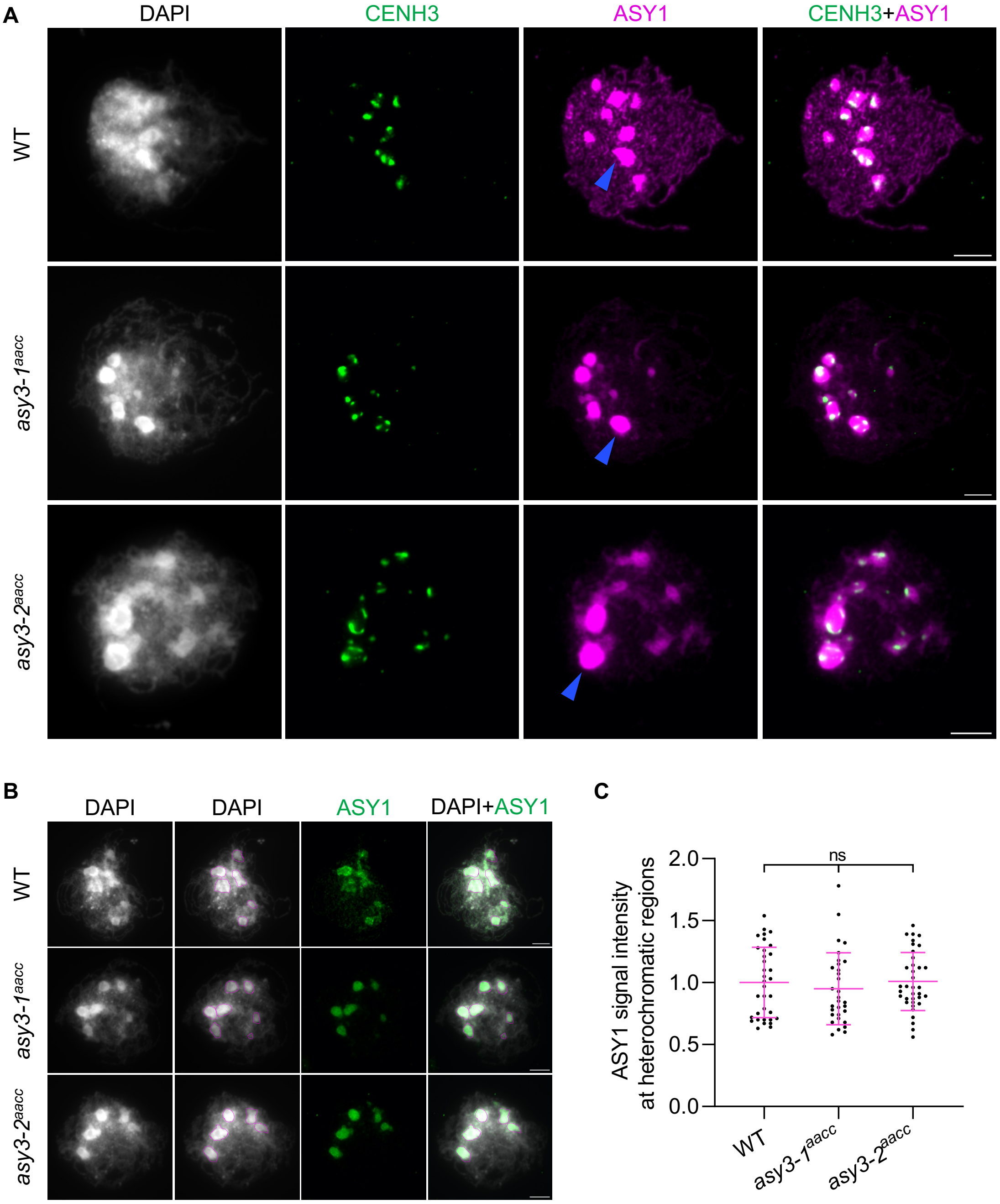

Supplementary Figure S5. Localization of ASY1 at the brightly DAPI-stained heterochromatic regions is independent of ASY3 (Supports Figure 2). (A) Co-immunolocalization of ASY1 and CENH3 in male meiocytes of WT, *asy3-1aacc*, and *asy3-2aacc* mutant plants at early prophase I. Blue arrowhead indicates one representative region of condensed heterochromatin. Bars: 5 µm. (B) Immunostaining of ASY1 in male meiocytes of WT, *asy3-1aacc*, and *asy3-2aacc* mutants at leptotene. Images were captured under a short exposure time to make sure that ASY1 signal at the brightly DAPI-stained heterochromatic

regions highlighted by circles in magenta were not overexposed. (C) Relative ASY1 signal intensity at the brightly DAPI-stained heterochromatic regions shown in (B) in WT, *asy3-1^{aacc}*, and *asy3-2^{aacc}* mutant plants at leptotene. Error bars indicate mean ± SD.

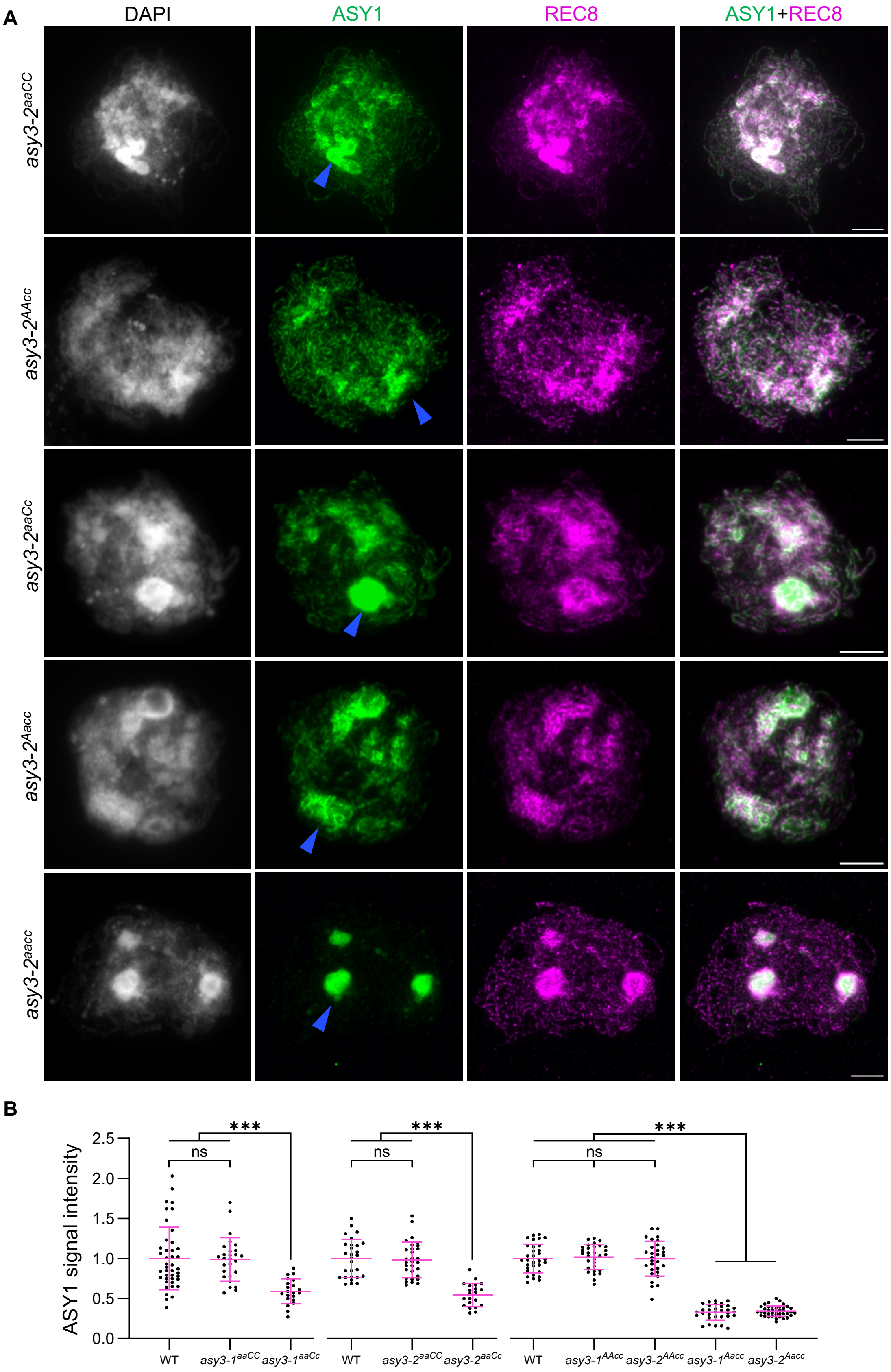

Supplementary Figure S6. Localization of ASY1 in *asy3* mutants (Supports Figure 2). (A) Co-immunostaining of ASY1 and REC8 in male meiocytes of

asy3-2^{aacc}, *asy3-2*^{aaCC}, *asy3-2*^{AAcc}, *asy3-2*^{aaCc}, and *asy3-2*^{Aacc} mutants at leptotene. Red arrowheads indicate the “blob”-like regions with overexposed signal. (B) Relative ASY1 signal intensity in WT, *asy3-1*^{aaCC}, *asy3-2*^{aaCC}, *asy3-1*^{aaCc}, *asy3-2*^{aaCc}, *asy3-1*^{AAcc}, *asy3-2*^{AAcc}, *asy3-1*^{Aacc}, and *asy3-2*^{Aacc} mutant plants at leptotene. The comparisons of signal intensity of WT with *asy3-1*^{aaCC} and *asy3-1*^{aaCc}, WT with *asy3-2*^{aaCC} and *asy3-2*^{aaCc}, and WT with *asy3-1*^{AAcc}, *asy3-2*^{AAcc}, *asy3-1*^{Aacc}, and *asy3-2*^{Aacc} mutant plants were plotted independently. Noting that the “blob”-like overexposed regions (red arrowheads) were removed from the quantification. Error bars indicate mean ± SD. Asterisks indicate significant difference (Game-Howell's multiple comparisons test, $p<0.001$).

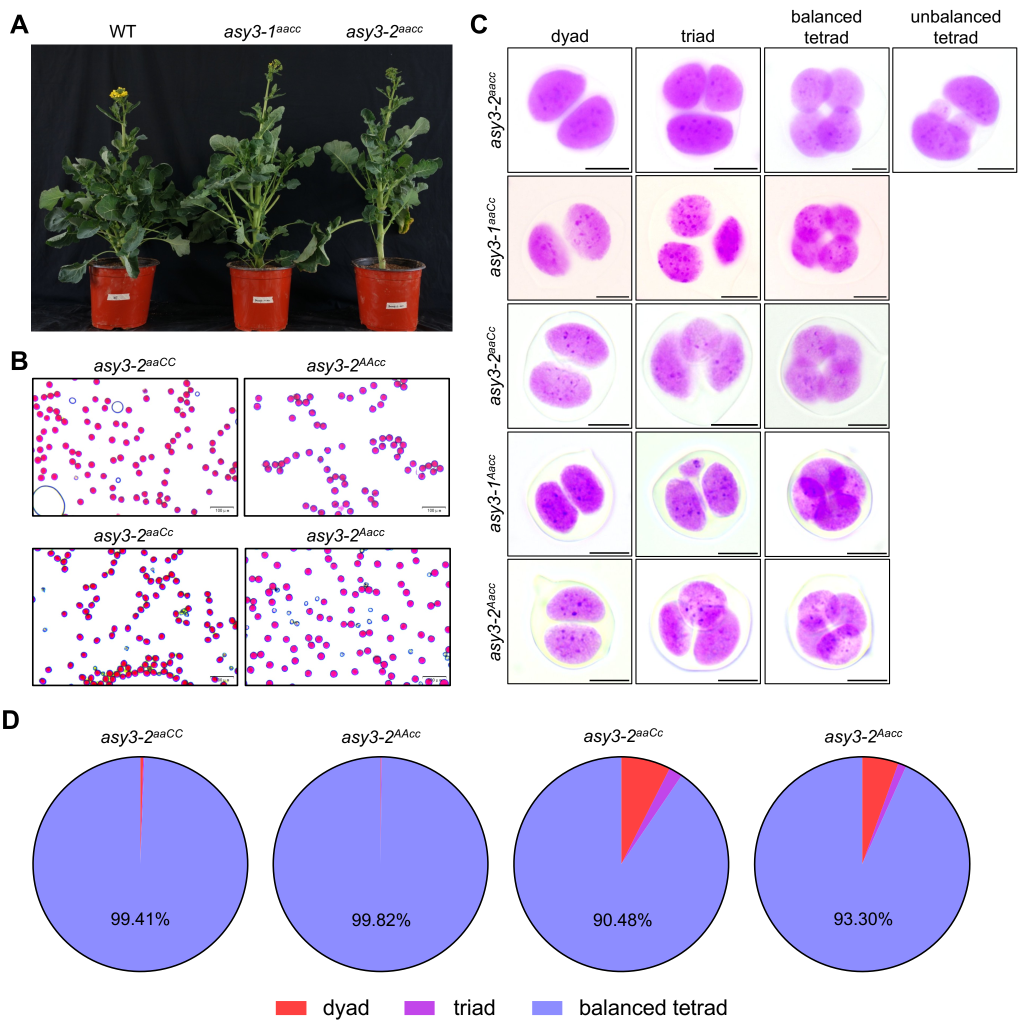

Supplementary Figure S7. Phenotypic analysis of *asy3* mutants (Supports Figure 3). (A) Plants of WT, *asy3-1^{aacc}*, and *asy3-2^{aacc}* mutants at early flowering stage. (B) Pollen staining of *asy3-2^{aaCC}*, *asy3-2^{AAcc}*, *asy3-2^{aaCc}*, and *asy3-2^{Aacc}* mutant plants. At least 4000 pollen grains were counted from different flowers for each genotype. Bars: 100μm. (C) Representative images of male meiotic products in *asy3-2^{aacc}*, *asy3-1^{aaCc}*, *asy3-2^{aaCc}*, *asy3-1^{Aacc}*, and *asy3-2^{Aacc}* mutant plants. Bars: 5μm. (D) Pie charts showing the proportion of balanced tetrad, unbalanced tetrad, triad, and dyad in WT, *asy3-2^{aaCC}*, *asy3-2^{AAcc}*, *asy3-2^{aaCc}*, and *asy3-2^{Aacc}* mutant plants.

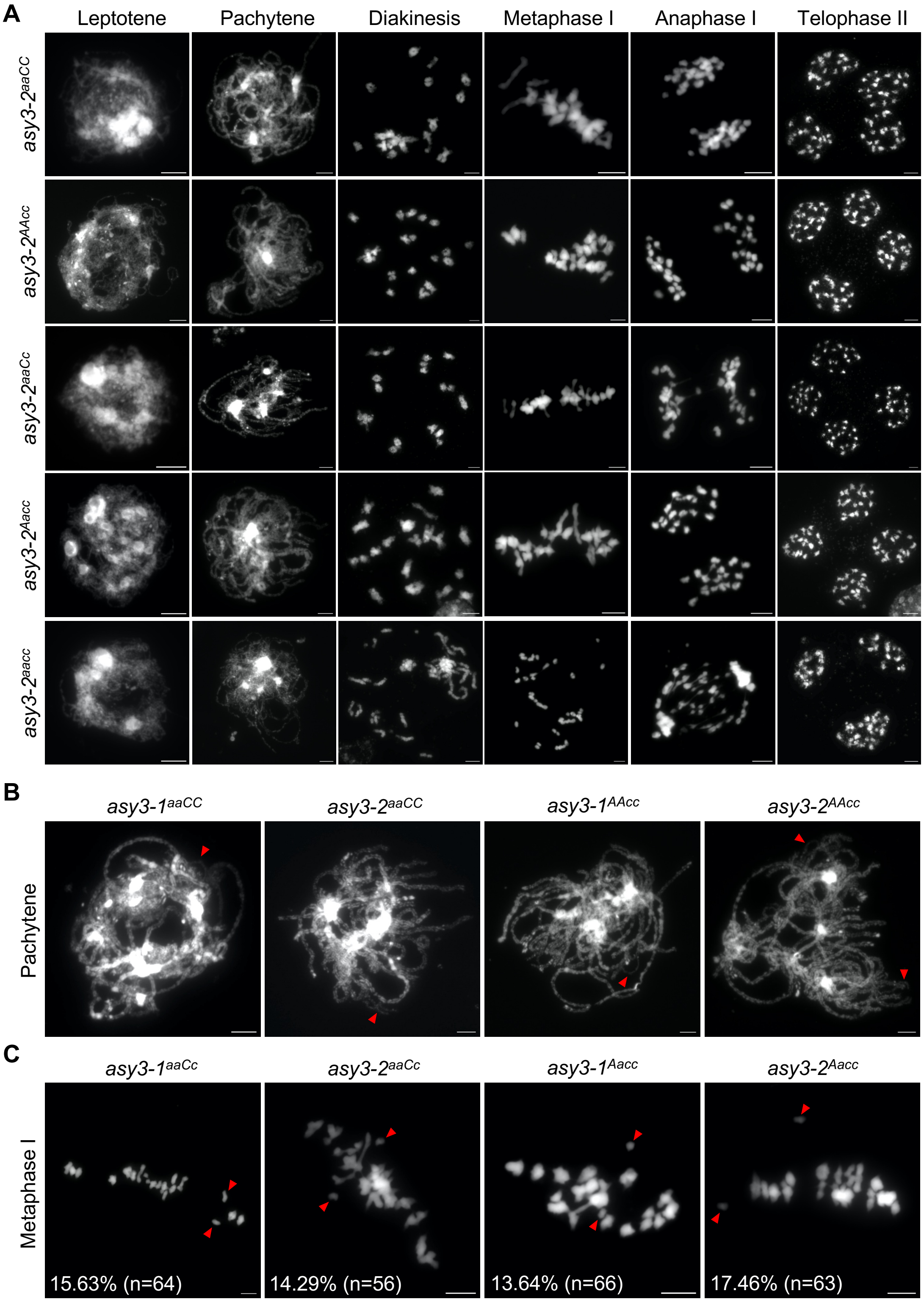

Supplementary Figure S8. Analysis of meiotic chromosome behaviors in *asy3* mutants (Supports Figure 4). (A) Chromosome spread analysis of male meiosis in *asy3-2*^{aaCC}, *asy3-2*^{AAcc}, *asy3-2*^{aaCc}, *asy3-2*^{Aacc}, and *asy3-2*^{aacc} mutants. (B) Representative images showing the unpaired stretches (red arrowheads) in

male meiocytes of *asy3-1*^{aaCC}, *asy3-2*^{aaCC}, *asy3-1*^{AAcc}, and *asy3-2*^{AAcc} at pachytene. (C) Representative cells having one pair of univalents (red arrowheads) in male meiocytes of *asy3-1*^{aaCc}, *asy3-2*^{aaCc}, *asy3-1*^{Aacc}, and *asy3-2*^{Aacc} at metaphase I. The percentages indicate the ratio of cells with one pair of univalents. Bars: 5μm.

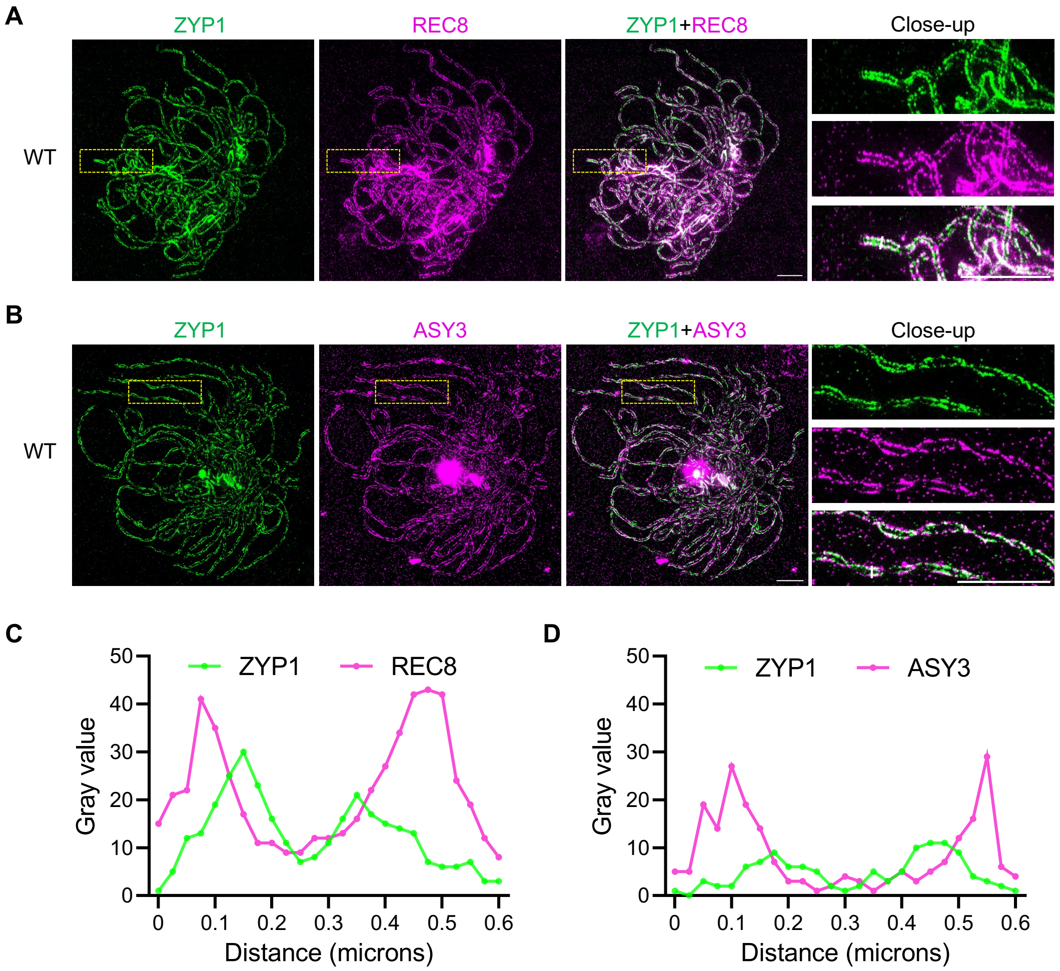

Supplementary Figure S9. Super resolution imaging of chromosome synapsis in male meiocytes of wildtype using STED (Supports Figure 5). (A) Co-immunostaining of ZYP1 and REC8 in male meiocytes of wildtype at pachytene. (B) Co-immunostaining of ZYP1 and ASY3 in male meiocytes of wildtype at pachytene. (C) Signal distribution profiles of ZYP1 and REC8 as shown in (A). The region used for analysis is indicated by the white line in the close-up panel of (A). (D) Signal distribution profiles of ZYP1 and ASY3 as shown in (B). The region used for analysis is indicated by the white line in the close-up panel of (B). Bars: 5 μm.

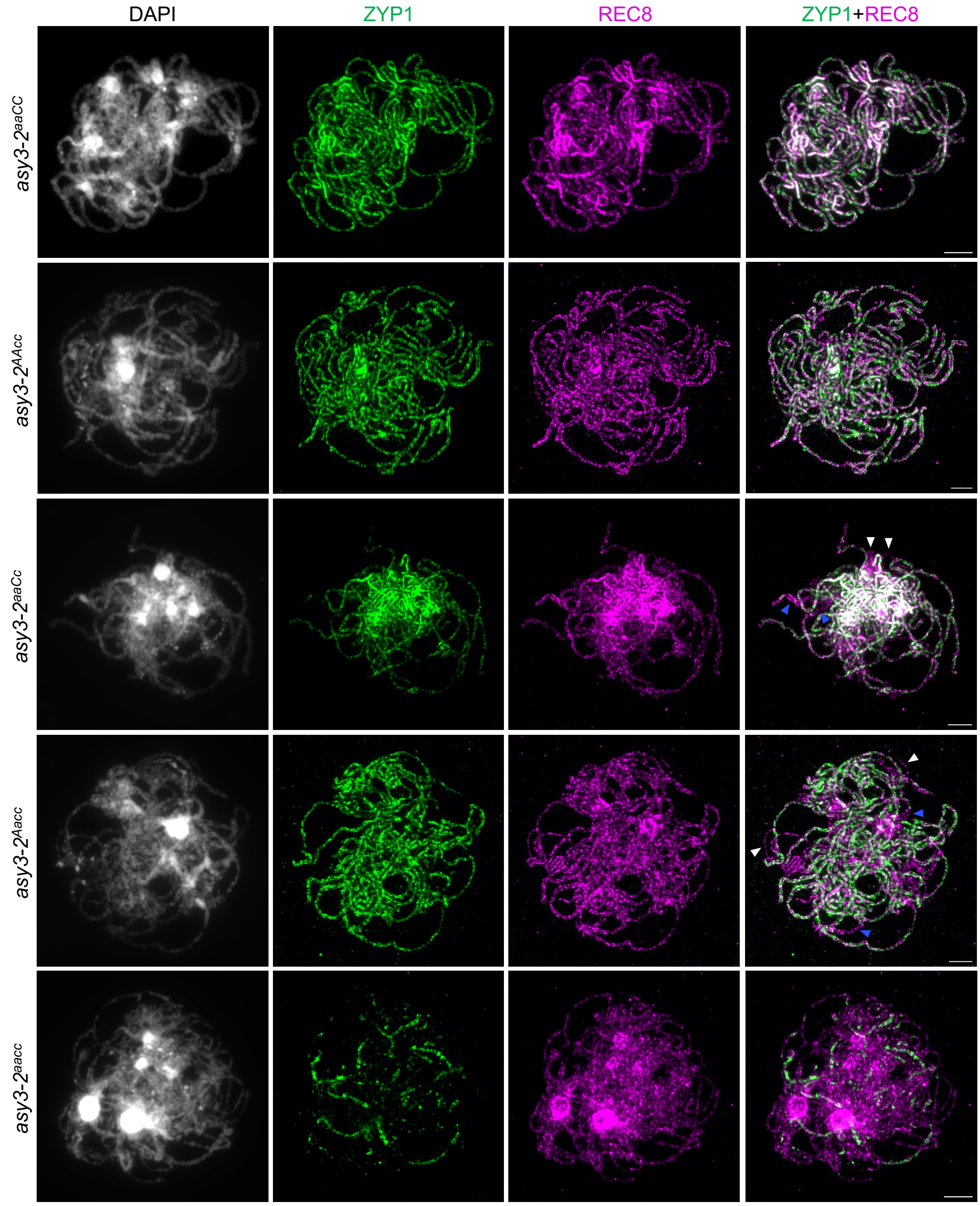

Supplementary Figure S10. ASY3 dosage-dependent effects on chromosome synapsis (Supports Figure 5). Co-immunostaining of ZYP1 and REC8 in male meiocytes of *asy3-2^{aaCC}*, *asy3-2^{AAcc}*, *asy3-2^{aaCc}*, *asy3-2^{Aacc}*, and *asy3-2^{aacc}* mutants at pachytene. White and blue arrowheads indicate the unpaired single threads or coaligned regions that both have no ZYP signal, respectively. Bars: 5 μm.

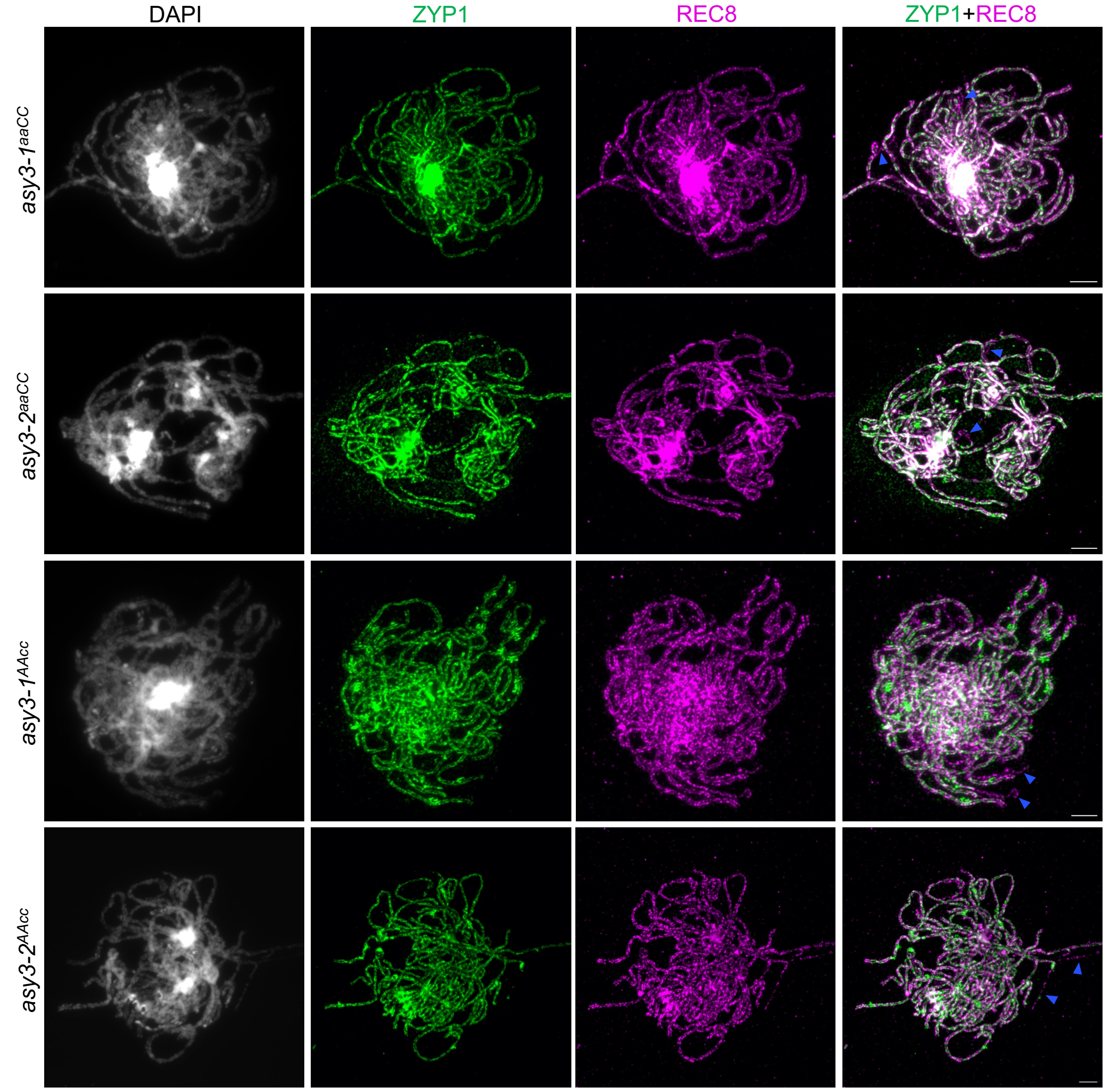

Supplementary Figure S11. Representative images of co-immunostaining of ZYP1 and REC8 show cells harboring some non-ZYP1 labeled regions in male meiocytes of *asy3-1*^{aaCC}, *asy3-2*^{aaCC}, *asy3-1*^{AAcc}, and *asy3-2*^{AAcc} at pachytene (Supports Figure 5). Blue arrowheads indicate non-ZYP1 labeled regions. Bars: 5 μm.

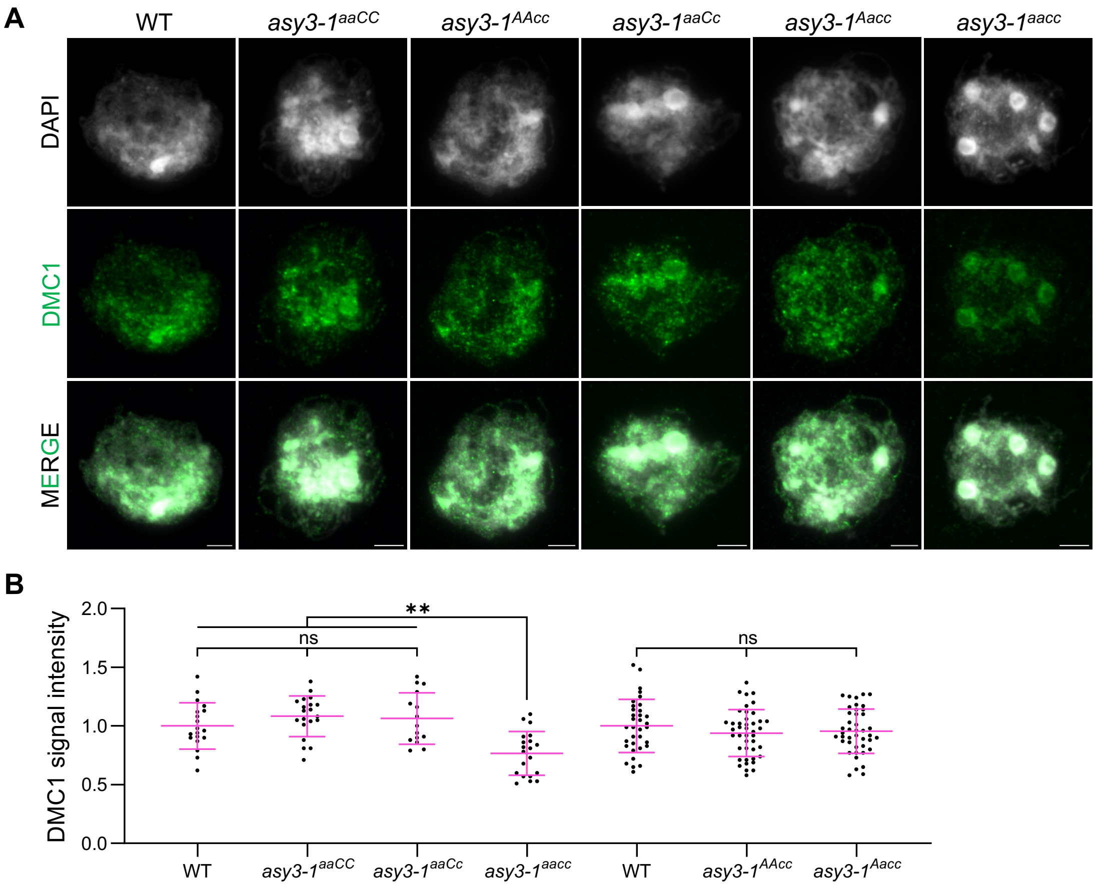

Supplementary Figure S12. Analysis of DMC1 localization in wildtype and *asy3* mutants (Supports Figure 4). (A) Immunolocalization of DMC1 in male meiocytes of WT, *asy3-1*^{aaCC}, *asy3-1*^{AAcc}, *asy3-1*^{aaCc}, *asy3-1*^{Aacc}, and *asy3-1*^{aacc} at early prophase I. Bars: 5 µm. (B) Quantification of relative DMC1 signal intensity shown in (A). Error bars indicate mean ± SD. **, $p < 0.01$. ns, no significance.

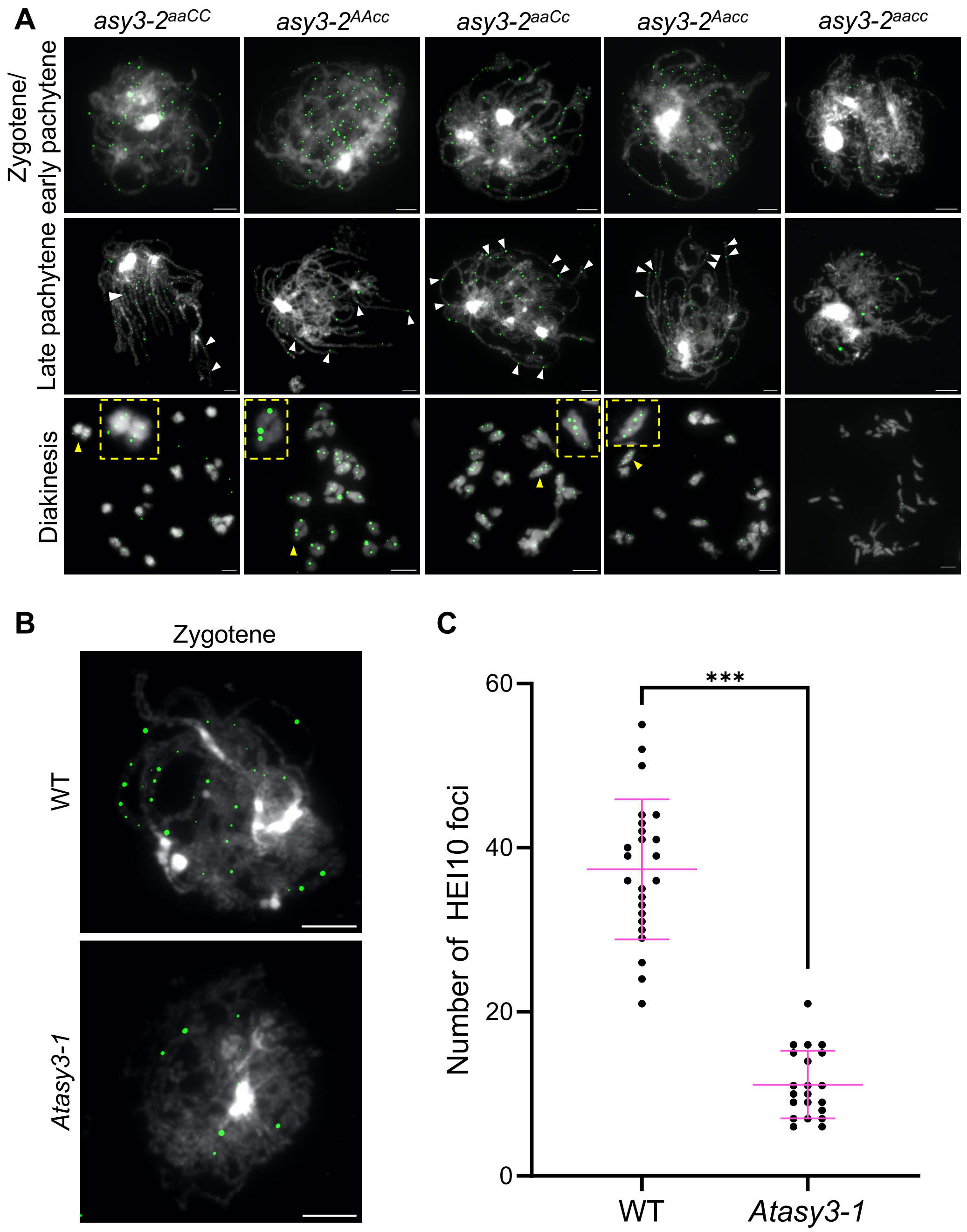

Supplementary Figure 13. Analysis of HEI10 localization in *asy3* mutants of *Arabidopsis* and *Brassica napus* (Supports Figure 6). (A) Immunolocalization of HEI10 in male meiocytes of *asy3-2*^{aaCC}, *asy3-2*^{AAcc}, *asy3-2*^{aaCc}, *asy3-2*^{Aacc}, and *asy3-2*^{aacc} mutants at zygotene/early pachytene, late pachytene, and diakinesis. White arrowheads indicate closely localized HEI10 foci alone one chromosome pair. Yellow arrowheads depict the magnified bivalents shown in the yellow rectangles. Bars: 5μm. (B) Immunolocalization of HEI10 at early prophase I in male meiocytes of *Arabidopsis* WT and *asy3-1* mutants. Bars:

5μm. (C) Quantification of the number of HEI10 foci in *Arabidopsis* WT and *asy3-1* mutants at early prophase I (zygotene/early pachytene). Error bars indicate mean ± SD. The statistical analysis was performed by student's t-test (*** p<0.001).

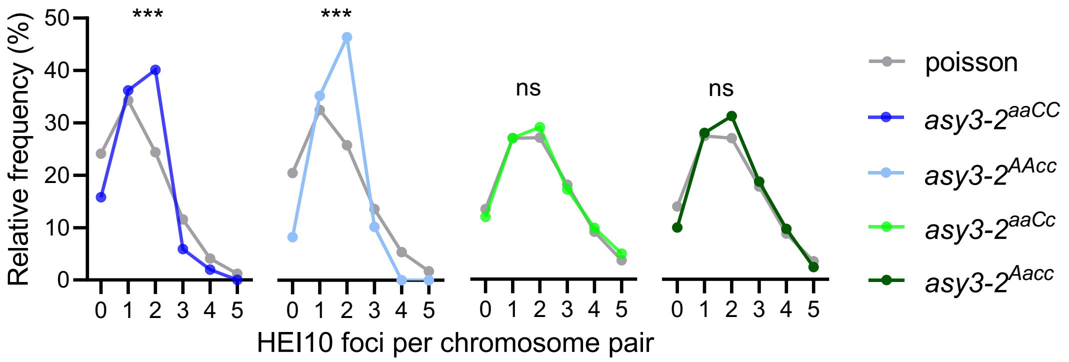

Supplemental Figure S14. Comparison of the observed and Poisson-predicted distributions of the number of HEI10 foci per chromosome pair (bivalent) in male meiocytes of *asy3-2*^{aaCC}, *asy3-2*^{AAcc}, *asy3-2*^{aaCc}, and *asy3-2*^{Aacc} mutant plants (Supports Figure 7). Chi-square test (*** p<0.001).

Supplementary Table S1. Summary of chromosome configurations at diakinesis/metaphase I in male meiocytes of wildtype and *asy3* mutants. Values represent the mean ± SD followed by the percentage.

Genotype	Ring bivalent	Rod bivalent	Univalent	Number of PMCs
Wild type	12.53±1.56 (65.95%) ^{b}	6.47±1.56 (34.05%) ^{a}	0.00±0.00 (0.00%)	32
asy3-1^{aaCC}	13.38±1.62 (70.39%) ^{b}	5.58±1.61 (29.34%) ^{ab}	0.10±0.44 (0.26%) ^{b}	40
asy3-2^{aaCC}	12.91±1.49 (67.94%) ^{b}	5.98±1.56 (31.46%) ^{ab}	0.23±0.63 (0.60%) ^{b}	44
asy3-1^{AAcc}	12.61±1.93 (66.37%) ^{b}	6.33±1.88 (33.33%) ^{a}	0.11±0.46 (0.29%) ^{b}	54
asy3-2^{AAcc}	13.26±1.59 (69.77%) ^{b}	5.70±1.62 (29.99%) ^{ab}	0.09±0.42 (0.24%) ^{b}	43
asy3-1^{aaCc}	14.88±1.64 (78.33%) ^{a}	4.00±1.63 (21.05%) ^{c}	0.24±0.64 (0.62%) ^{b}	51
asy3-2^{aaCc}	14.61±1.70 (76.91%) ^{a}	4.20±1.66 (22.13%) ^{bc}	0.36±0.77 (0.96%) ^{b}	44
asy3-1^{Aacc}	15.30±1.66 (80.54%) ^{a}	3.55±1.61 (18.67%) ^{c}	0.30±0.72 (0.79%) ^{b}	53
asy3-2^{Aacc}	15.33±1.56 (80.66%) ^{a}	3.53±1.52 (18.55%) ^{c}	0.30±0.71 (0.79%) ^{b}	40
asy3-1^{aacc}	0.49±0.73 (2.58%) ^{c}	5.06±1.41 (26.63%) ^{b}	26.90±2.78 (70.79%) ^{a}	100
asy3-2^{aacc}	0.40±0.58 (2.13%) ^{c}	5.02±1.71 (26.44%) ^{bc}	27.14±3.24 (71.43%) ^{a}	42

Supplementary Table S2. Summary of the number of HEI10 foci, total number of COs, and number of type II COs in male meiocytes of wildtype and *asy3* mutants. Values represent the mean ± SD followed by the number of observed nuclei.

Genotype	Total number of COs per PMC	Number of HEI10 foci per PMC			Number of type II COs per PMC
		zygotene & early pachytene	late pachytene & diplotene	diakinesis	
Wild type	33.97±2.54 (n=29) ^{b}	134.90±33.88 (n=21) ^{a}	26.59±3.99 (n=61) ^{c}	26.88±3.62 (n=48) ^{b}	6.55±3.09 (n=29) ^{a}
asy3-1^{aaCC}	36.59±4.06 (n=17) ^{b}	130.73±17.04 (n=11) ^{a}	29.65±4.30 (n=66) ^{b}	29.76±5.69 (n=41) ^{b}	6.29±3.37 (n=17) ^{a}
asy3-2^{aaCC}	34.63±2.55 (n=8) ^{b}	131.60±17.60 (n=10) ^{a}	29.72±4.06 (n=39) ^{b}	29.62±5.16 (n=21) ^{b}	7.63±2.78 (n=8) ^{a}
asy3-1^{AAcc}	36.45±2.87 (n=11) ^{b}	135.30±24.71 (n=27) ^{a}	32.02±4.64 (n=61) ^{b}	29.40±4.59 (n=30) ^{b}	6.82±2.59 (n=11) ^{a}
asy3-2^{AAcc}	35.44±2.45 (n=16) ^{b}	135.24±24.73 (n=21) ^{a}	31.21±3.75 (n=52) ^{b}	28.79±3.95 (n=24) ^{b}	5.31±1.96 (n=16) ^{a}
asy3-1^{aaCc}	44.26±4.20 (n=23) ^{a}	123.77±22.52 (n=26) ^{a}	38.38±6.40 (n=29) ^{a}	37.91±5.78 (n=58) ^{a}	6.00±3.56 (n=23) ^{a}
asy3-2^{aaCc}	44.73±4.60 (n=15) ^{a}	126.81±31.38 (n=21) ^{a}	40.24±6.91 (n=37) ^{a}	37.66±6.03 (n=44) ^{a}	6.60±3.46 (n=15) ^{a}
asy3-1^{Aacc}	42.60±4.59 (n=20) ^{a}	129.38±24.76 (n=24) ^{a}	40.71±7.54 (n=55) ^{a}	36.67±5.58 (n=33) ^{a}	6.50±3.12 (n=20) ^{a}
asy3-2^{Aacc}	43.22±3.22 (n=18) ^{a}	133.15±19.18 (n=20) ^{a}	41.18±5.51 (n=39) ^{a}	37.21±4.30 (n=34) ^{a}	5.72±2.62 (n=18) ^{a}
asy3-1^{aacc}	6.04±1.71 (n=100) ^{c}	12.43±3.92 (n=30) ^{b}	6.76±1.75 (n=41) ^{d}	5.48±2.20 (n=44) ^{c}	~ 0.56
asy3-2^{aacc}	5.83±1.73 (n=42) ^{c}	10.95±2.33 (n=22) ^{b}	6.87±2.19 (n=15) ^{d}	5.17±2.13 (n=24) ^{c}	~ 0.66

Supplementary Table S3. Primers used in this research.

Primer name	Sequence (5' to 3')
BnaASY3-DT1-BsF	ATATATGGTCTCGATTGGATTTACAGCTGAACAAGAGTT
BnaASY3-DT1-F0	TGGATTTACAGCTGAACAAGAGTTTTAGAGCTAGAAATAGC
BnaASY3-DT2-R0	AACCTGCTTGGCAGCTACATCACAATCTCTTAGTCGACTCTAC
BnaASY3-DT2-BsR	ATTATTGGTCTCGAAACCTGCTTGGCAGCTACATCACAA
qPCR-BnaA05.ASY3-F	GATAAGAGATTGCATGACTTCTTGG
qPCR-BnaA05.ASY3-R	GTTGTAATGGCCCAACGCGGGTGAG
qPCR-BnaC04.ASY3-F	GATAAGAGATTGCCTGACTTCTTGG
qPCR-BnaC04.ASY3-R	TACTTGGGGATTGTTGTAATGGGAG
BnaASY1-A07-attB1F	GGGGACAAGTTTGTACAAAAAAGCAGGCTTCATGGTAATGGCTCAGAAGCTCAAGG
BnaASY1-A07-300aa-attB2R	GGGGACCACTTTGTACAAGAAAGCTGGGTTTCATTTACTCAGTTTAGTGAAAAACTT
BnaASY3-A05-attB1F	GGGGACAAGTTTGTACAAAAAAGCAGGCTTCATGAGCGAATACAGGAGCTTCGGCAG
BnaASY3-A05-attB2R	GGGGACCACTTTGTACAAGAAAGCTGGGTTTCAACCTAACACATCATCCTTCAAAC
BnaASY3-A05-32aa-attB2R	GGGGACCACTTTGTACAAGAAAGCTGGGTTTCATCTCTTGGGCTGTGAATCAGCC
nLUC-BnaA07.ASY1-F	GGACGAGCTCGGTACCATGGTAATGGCTCAGAAGCTCAAG
nLUC-BnaA07.ASY1-1-300aa-R	GCGTACGAGATCTGGTCGACAGACTCAACCGGATCCTGTG
cLUC-BnaA05.ASY3-F	GTCCCGGGGCGGTACCATGAGCGAATACAGGAGCTTCG
cLUC-BnaA05.ASY3-FL-R	CGAAAGCTCTGCAGGTCGACTCAACCTAACACATCATCCTTCAAACATTCTG
cLUC-BnaA05.ASY3-1-32aa-R	CGAAAGCTCTGCAGGTCGACTCATCTCTTGGGCTGTGAATCAGCCATAACTCCAA
BnaASY3-A05-F1	CAGAAGAATGCTTAACCTTCTGTGGTGATC
BnaASY3-C04-F1	GAAGAATGCTTAACCTTCTGTGGTAATAAA
BnaASY3-R1	AGATGGAGGTTCTTGCATCCTCT
