# Supplementary material for: ASYNAPSIS3 has diverse dosage-dependent effects on meiotic crossover formation in Brassica napus
Source: Plant Cell. 2024 Jul 24;36(9):3838–56. doi: 10.1093/plcell/koae207 (PMC11371185; doi:10.1093/plcell/koae207)
Supplement: koae207_Supplementary_Data [file tpc.00170.2024-s07.pdf › tpc.00170.2024-s07.pdf]

THE PLANT CELL

ASY3 has dosage-dependent diverse effects on meiotic crossover formation

Lei Chu, Jixin Zhuang, Miaowei Geng, Yashi Zhang, Jing Zhu, Chunyu Zhang, Arp Schnittger, Bin Yi, and Chao Yang

Review Timeline:

Submission Date:	09-Jan-2024
Editorial Decision:	13-Feb-2024
Resubmission Received:	27-May-2024
Editorial Decision:	09-Jun-2024
Revision Received:	24-Jun-2024
Accepted:	24-Jun-2024

Dr. Chao Yang
Huazhong Agricultural University
Wuhan, Hubei 430070
China

Dear Dr. Yang:

Thank you for choosing to send your manuscript entitled "ASY3 has dosage-dependent diverse effects on meiotic crossover formation" for consideration at The Plant Cell. Your submission has been evaluated by members of the editorial board as well as expert reviewers in your field, and we regret to inform you that we are not able to recommend the publication of this manuscript in its current form. We have had input from multiple scientists and we have solicited post-review comments.

During the post-review consultation session, we nevertheless agreed that if you could address the major points raised by the reviewers by new experiments, we would welcome a resubmission. This will be treated as a new submission, but we would attempt to use at least some of the same reviewers. Nevertheless, reviewers will be asked to assess as a new manuscript (i.e. are the claims fully supported by the data and do the results presented move the field forward?), and not only whether previous reviewer comments have been addressed.

The referees have appreciated the quality of the work and of the data presented. They also agree that controlling the dosage of ASY3 is a potentially interesting way to control crossovers in polyploid Brassica breeding. However, the referees (in particular # 2 and 3) had concerns expressed in their review and in a subsequent consultation. As a study whose novelty is in studying dosage response in a polyploid, they thought that it was a weakness not to have included asy3AAcc and asy3Aacc mutants (and possibly other dosage combinations). It is not to be taken for granted that the ASY3 orthologs act in a similar manner. In addition, the reviewers felt that measuring the effect of ASY3 on crossover rate and interference was done at a relatively low resolution and that a more direct mapping of crossovers would make the work stronger. Additional comments can be seen in the reviews. Considering the extensive time needed to address the two main concerns mentioned above (dosage series and higher crossover resolution), and considering the reviewer's appreciation of the quality of the work, we encourage you to resubmit a revised version.

In addition, we find that the writing suffers from some problems with grammar and awkward phrasing in particular in the introduction (See Reviewer 1 comments). We strongly encourage the use of a professional editing service to ensure that the work is presented in the best manner and reaches the broadest possible audience. Several editing services have agreed to extend a discount to authors coming to their website via this ASPB page https://aspb.org/aspb-journal-submission-editing-services/.

The Plant Cell now requires authors to complete and submit an author revisions checklist upon submission of a revised manuscript. The aim of the checklist is to aid authors in preparing a high-quality manuscript, facilitate the review and assessment of revised manuscripts, and help to ensure that journal standards are maintained across the board. If your manuscript is accepted, the completed checklist will be published as supplemental material attached to the article online. Please download a copy of the checklist (a fillable PDF form) at this link, for submission with your revised manuscript: https://tpc.msubmit.net/html/Author_Revisions_Checklist.pdf.

Note also that supplemental materials should be restricted to large datasets and tables, presentation of replicates, and validation of reagents, methods, or genotypes. Any data that are used to support the main claims must be in the main manuscript. Supplemental figure legends must indicate what figure in the main manuscript is supported by the supplemental data presented.

We thank you for your interest in and support of The Plant Cell. We hope we will be able to render a more positive decision on future work.

On behalf of the editorial board,

Reviewing Editor, Board of Reviewing Editors
James Birchler, Senior Editor
Blake Meyers, Editor-in-Chief

The Plant Cell

--- Reviewer comments:

Reviewer #1 (Comments for the Author):

Chu et al. have utilised hypomorphic asy3 mutants in Brassica napus to investigate the role of the meiotic chromosome axis on synaptonemal complex formation and recombination. An increase in the number of HEI10 foci that mark class I crossovers is observed in asy3-1aaCa and asy3-2aacc mutants which is rather striking and may provide a useful tool for plant breeders. The phenotype is consistent with a loss/attenuation of crossover interference, similar to the Arabidopsis zyp1 mutant and may reflect perturbation of the normal function of the SC. The interpretation of the data is reasonable, but it could also be argued that interference is still promoting evenly spaced HEI10 foci, even though there are considerably more in the hypomorphic mutants. This would be more similar to the crossover distribution in budding yeast. However, the Chi-square analysis in asy3-1aaCa and asy3-2aacc, supports an interpretation of loss of interference.

The introduction can be improved with editing, but the results and discussion is well written.

Other points:

Introduction
Linkage drags should be linkage drag
Lines 46-56 is badly written. This should be three, not one sentence and refer to CO pathways, not machineries. The references should be at the end of each sentence and appears that the references are inadequate for the statements that are written.
This is a strange sentence and should refer to published work on analysis of rec8 mutants in plant meiosis:
The cohesin 61 complexes, encompassing
62 the duplicated sister chromatids, are thought to build the basis of the axis in plants as
63 well as in most, if not all, of other species.

The Armstrong reference should have other authors and it's not even used in the right context as it doesn't analyse an asy1 mutant.
Armstrong SJ (2002) Asy1, a protein required for meiotic chromosome synapsis,
657 localizes to axis-associated chromatin in Arabidopsis and Brassica. J. Cell Sci.,
658 115: 3645-3655
Asy1, a protein required for meiotic chromosome synapsis, localizes to axis-associated chromatin in Arabidopsis and Brassica
Susan J Armstrong 1, Anthony P Caryl, Gareth H Jones, F Christopher H Franklin. 2002 Sep 15;115(Pt 18):3645-55. J Cell Sci
doi: 10.1242/jcs.00048.

Results
Figure 2. The 'blob' signal is most likely heterochromatin comprised of densely packed DNA. It's probably the centromeres/pericentromeres forming the synizetic knot as these signals are separate in Supplemental Figure 4A asy3-2aacc.

Reviewer #2 (Comments for the Author):

This study investigates meiotic recombination in allotetraploid Brassica napus, focusing on the role of the axis protein ASY3. Mutation of all 4 copies of ASY3 result in defective synapsis and reduced crossovers, whereas a single copy can rescue function, although crossover interference appears be defective as events are now randomly distributed.

The authors identify two ASY3 orthologs in the A and C subgenomes. Using CRISPR the authors isolate mutations in each of these genes. Different combination of mutants were isolated, including aacc, aa, aac, but not cc or acc unfortunately.

An ASY3 antibody was used to monitor expression during meiosis, and how this was influenced by the mutations, with reduced protein accumulation detected in some mutant combinations. Further REC8 staining was shown to be normal in aacc mutants, whereas ASY1 was diminished. Using the lower combinations of mutants, fertility and meiotic immunostaining are largely normal, but there is some dosage effects. In contrast the aaCc mutant shows an intermediate level of staining.

Interestingly, in Figure 2, it seems that ASY1 and REC8 are brightly staining the densely DAPI staining chromocentres. This is reminiscent of ASY1 and REC8 ChIP-seq in Arabidopsis, which both showed strong enrichment in the heterochromatin - I wonder if this is related? It also appears that ASY1 and REC8 are still quite strongly present on heterochromatin in the aacc mutant - how do the authors interpret this? Could ASY1 be recruited to heterochromatin independent of ASY3? Although this appears to be much less the case in the aacc images shown in Figure 5 at synapsis?

BnASY3 is shown to interact with BnASY1 using a two hybrid assay, which is expected from studies in Arabidopsis.

The potentially most interesting aspect of the study comes from crossover estimates. First this is performed using chiasma scoring. First comparing aacc with wild type an expected large increase in univalents is observed. However, in aaCc mutants the authors report an increase in ring type bivalents consistent with an increase in crossovers. However, the proportion of ring to rod bivalents is a relatively imprecise method of scoring crossovers and can for example, underestimate closely spaced crossovers.

To provide additional insight the authors stain for HEI10, a protein that localises at Class I crossover sites. As expected, the number of HEI10 foci is reduced in aacc compared to wild type. Consistent with the chiasmata counts, the authors find that in aaCc HEI10 foci are increased. The authors then estimate class II crossovers indirectly by scoring bivalents that lack HEI10 foci, the authors conclude that Class II events are normal in aaCc, and therefore the effects seen in this background are only on Class I. Finally the authors observe clustered HEI10 foci in aacc mutants more than wild type, suggesting a defect in crossover interference.

Would the authors expect the same results as aaCc in an aAcc background? Equally, would they expect the same results in as ASY1 aaCc mutant?

Minor points:

The first three paragraphs of the introduction are inadequately referenced.

Lines 75-79. Its not clear which mutants this statement is referring to - for example cohesin is described in the preceding sentences, but this statement would not apply to a rec8 mutant for example.

Line 241 - remove 'a bit'.

Line 516 - reference citation seems to be after period.

Reviewer #3 (Comments for the Author):

During meiosis, the number and distribution of crossovers (COs) are tightly controlled. Synaptonemal complex is dispensable for CO formation and has a key role in regulating their number and distribution, imposing CO interference and heterochiasmy in Arabidopsis. In this manuscript, the authors reported results of ASY3, the axis protein and its diverse dosage control on meiotic crossover formation in Brassica napus. The authors showed that the absence of all four ASY3 alleles results in defective synapsis and drastic reduction of crossovers, which is largely rescued by the presence of only one functional ASY3 allele. These findings implied that ASY3 dosage has a strong effect on chromosome synapsis, crossover number and distribution.

This is an interesting paper that addresses the role of a conserved chromosome axis protein, ASY3, in meiotic recombination in a polyploid plant (Brassica napus). However, the task itself is not novel. The conclusions are to some extent expected as HEI10 dosage is known to regulate levels of homologous recombination in Arabidopsis (Ziolkowski 2017). It may also be worth mentioning that the number of crossovers formed between homoeologous responds to MSH4 dosage in Brassica napus (Adrián Gonzalo 2019). There are also a number of major and minor issues with the paper that the authors should address.

Major:
1. Line 133-136, the authors suggested that they failed to obtain such mutants of asy3AAcc and asy3Aacc. Can the authors give a deeper reason rather than simply describing the phenomenon? Are there specific sequences of BnaA05.ASY3 and BnaC04. ASY3 respectively? It would be useful to obtain such mutants by designing specific targets of the C sub-genome harbor to obtain asy3AAcc, which can cross with asy3aacc to obtain asy3Aacc . Meanwhile, the transcriptional behavior of BnaA05.ASY3 and BnaC04.ASY3 during male meiosis should be analyzed. Additionally, the progression of male meiosis should be analyzed in asy3AAcc. Only an asy3aaCC -like meiosis observed in asy3AAcc can indicate that BnaA05.ASY3 and BnaC04.ASY3 are both equally functional and able to complement one another.
2. Why was only ASY3 investigated/reported on? What about other synaptonemal complex genes? The authors need to provide some more justification for why only ASY3 was investigated in the introduction, or if other genes were also considered (at least bioinformatically) then this information should be in the results or supplementary information. There must have been some rationale based on functionality etc., unless all the synaptonemal complex genes mentioned were investigated and then this one turned out to be the key candidate.
3. Line 357-360, "...non-HEI10 labeled chiasmata were treated as class II COs." This statement is seemingly inappropriate, because HEI10 is the only protein of catalyzing class I COs. Are there markers indicating Class II COs to measure class II COs more accurately?
4. Figure 7E: the authors performed a distribution analysis for HEI10 focus number per bivalent at diakinesis by cytology suggesting that CO interference is compromised when ASY3 dosage is reduced. Whether the location of the very rare crossovers which was significantly distinct from the Poisson distribution that are formed in the asy3 completely defective allohaploid mutants? It would be better for the CO identification and analysis in the asy3 by generating segregating populations and whole-genome sequencing.

A few minor comments are listed below:
1. Line 166-169, in protein interaction studies one yeast-two hybrid (Y2H) positive result cannot rule out the possibility of false

positives. It is prudent to show at least one more results such as Co-Immunoprecipitation (Co-IP), bimolecular fluorescence complementation (BiFC) or Split-luciferase Complementation Imaging (LCI).
2. Line 172-173 and 311-312, the authors used Arabidopsis asy1-4 and hei10-2 mutants to verify the specificity of the ASY1 and HEI10 antibodies. Based on Figure 6A, HEI10 foci can be observed in the cytoplasm, which makes it doubtful for the specificity of the HEI10 antibody. Can the authors use Brassica napus asy1 and hei10 mutants to verify the specificity of the ASY1 and HEI10 antibodies? Please prove it.
3. Line 232-237, using DAPI-staining to estimate the chromosome juxtaposition is not accurate, maybe you can observe chromosome behaviors with antibodies or probes?
4. Line 294-295, in the asy3-1aaCc and asy3-2aaCc mutants, only ~20 cells were observed to estimate the number of chiasmata, while the increase of the ratio in the ring-type bivalents is susceptible. More cells should be observed to support the point.

Reviewer #1 (Comments for the Author):

Chu et al. have utilised hypomorphic asy3 mutants in Brassica napus to investigate the role of the meiotic chromosome axis on synaptonemal complex formation and recombination. An increase in the number of HEI10 foci that mark class I crossovers is observed in asy3-1aaCa and asy3-2aacc mutants which is rather striking and may provide a useful tool for plant breeders. The phenotype is consistent with a loss/attenuation of crossover interference, similar to the Arabidopsis zyp1 mutant and may reflect perturbation of the normal function of the SC. The interpretation of the data is reasonable, but it could also be argued that interference is still promoting evenly spaced HEI10 foci, even though there are considerably more in the hypomorphic mutants. This would be more similar to the crossover distribution in budding yeast. However, the Chi-square analysis in asy3-1aaCa and asy3-2aacc, supports an interpretation of loss of interference.

The introduction can be improved with editing, but the results and discussion is well written.

We thank this reviewer for his/her time to read and comment on our paper. We also appreciate very much his/her suggestions, which have helped to improve this manuscript. We have now rewritten the introduction. We hope the reviewer and editors would find that the introduction is now improved in writing in the revised manuscript.

Other points:

Introduction
Linkage drags should be linkage drag

Thanks for pointing out this mistake. We corrected this as suggested.

Lines 46-56 is badly written. This should be three, not one sentence and refer to CO pathways, not machineries. The references should be at the end of each sentence and appears that the references are inadequate for the statements that are written.

As suggested, we have rephrased this part into three sentences and added relevant references at the end of each sentence.

This is a strange sentence and should refer to published work on analysis of rec8 mutants in plant meiosis: The cohesin complexes, encompassing

the duplicated sister chromatids, are thought to build the basis of the axis in plants as well as in most, if not all, of other species.

We have rephrased this sentence and referred to the papers on the analysis of rec8 mutants in Arabidopsis, rice, and maize.

The Armstrong reference should have other authors and it's not even used in the right context as it doesn't analyse an asy1 mutant.
658 Armstrong SJ (2002) Asy1, a protein required for meiotic chromosome synapsis, localizes to axis-associated chromatin in Arabidopsis and Brassica. J. Cell Sci., 115: 3645-3655
Asy1, a protein required for meiotic chromosome synapsis, localizes to axis-associated chromatin in Arabidopsis and Brassica
Susan J Armstrong 1, Anthony P Caryl, Gareth H Jones, F Christopher H Franklin. 2002 Sep 15;115(Pt 18):3645-55. J Cell Sci doi: 10.1242/jcs.00048.

We have corrected this and referred to the correct article throughout the whole manuscript.

Results
Figure 2. The 'blob' signal is most likely heterochromatin comprised of densely packed DNA. It's probably the centromeres/pericentromeres forming the synizetic knot as these signals are separate in Supplemental Figure 4A asy3-2aacc.

We thanks this reviewer for pointing this out. We fully agree that these 'blob' signal is very likely from the centromeric and heterocentromeric DNA. In this revised manuscript, we performed the dual immunostaining of ASY1 with CENH3 (a centromere-specific histone) and quantified the signal intensity of ASY1 at the densely DAPI staining heterochromatin in the wildtype and asy3^{aacc} mutants. Based on these results, we concluded that ASY1 shows an enrichment on the chromocenters, which is independent on the presence of ASY3. We described in the revised manuscript as follow:

"Notably, a strong accumulation of ASY1 and REC8 in the wildtype was detected at regions that are more brightly stained with DAPI indicative of the chromocenters comprising of centromeric and pericentromeric DNA (Figure 2), as confirmed by the co-immunostaining of ASY1 with CENH3 (a centromere specific histone protein) (Supplemental figure 5A). This is a reminiscence of the previous studies showing that ASY1 and REC8 exhibit strong enrichments at the heterochromatin in Arabidopsis (Lambing et al, 2020a, 2020b). Unexpectedly, the accumulation of ASY1 at the densely DAPI-stained heterochromatic regions was not visibly affected by the absence of ASY3 (Figure 2, Supplemental figure 5A, red arrowheads). We quantified the signal

intensity of ASY1 from the densely DAPI-stained regions with non-overexposed images at leptotene (see method) and found no difference between the wildtype and asy3^{aacc} mutants, suggesting that ASY1 loading at the chromocenters is independent of ASY3 in Brassica napus (Supplemental figure 5B and C).”

Reviewer #2 (Comments for the Author):

This study investigates meiotic recombination in allotetraploid Brassica napus, focusing on the role of the axis protein ASY3. Mutation of all 4 copies of ASY3 result in defective synapsis and reduced crossovers, whereas a single copy can rescue function, although crossover interference appears be defective as events are now randomly distributed.

The authors identify two ASY3 orthologs in the A and C subgenomes. Using CRISPR the authors isolate mutations in each of these genes. Different combination of mutants were isolated, including aacc, aa, aac, but not cc or acc unfortunately.

An ASY3 antibody was used to monitor expression during meiosis, and how this was influenced by the mutations, with reduced protein accumulation detected in some mutant combinations. Further REC8 staining was shown to be normal in aacc mutants, whereas ASY1 was diminished. Using the lower combinations of mutants, fertility and meiotic immunostaining are largely normal, but there is some dosage effects. In contrast the aaCc mutant shows an intermediate level of staining.

We thank this reviewer for his/her time to read and comment on our paper. We appreciate very much his/her suggestions that have promoted our paper.

Interestingly, in Figure 2, it seems that ASY1 and REC8 are brightly staining the densely DAPI staining chromocentres. This is reminiscent of ASY1 and REC8 ChIP-seq in Arabidopsis, which both showed strong enrichment in the heterochromatin - I wonder if this is related? It also appears that ASY1 and REC8 are still quite strongly present on heterochromatin in the aacc mutant - how do the authors interpret this? Could ASY1 be recruited to heterochromatin independent of ASY3? Although this appears to be much less the case in the aacc images shown in Figure 5 at synapsis?

We thank this reviewer for bringing up this point. As suggested also by the first reviewer (see above), we fully agree with the interpretation that the high accumulation of ASY1 and REC8 at the heterochromatin (centromeres/pericentromeres) in both wildtype and asy3^{aacc} mutants, possibly

suggests that the recruitment of ASY1 to heterochromatin is likely independent of ASY3. To verify this hypothesis, in the revised manuscript we first confirmed that those brightly DAPI staining regions are mainly chromocenters by performing the dual immunostaining of ASY1 with CENH3 (a centromere-specific histone). Moreover, we quantified the signal intensity of ASY1 at the densely DAPI staining heterochromatin in the wildtype and $asy3^{aacc}$ mutants by reacquiring the immunostaining images that are not overexposed at the regions of chromocenters (see method and supplemental figure 5B and C). The quantification shows that the protein level of ASY1 at the chromocenters is not affected by the loss of ASY3.

We described in the revised manuscript as follow:

"Notably, a strong accumulation of ASY1 and REC8 in the wildtype was detected at regions that are more brightly stained with DAPI indicative of the chromocenters comprising of centromeric and pericentromeric DNA (Figure 2), as confirmed by the co-immunostaining of ASY1 with CENH3 (a centromere specific histone protein) (Supplemental figure 5A). This is a reminiscence of the previous studies showing that ASY1 and REC8 exhibit strong enrichments at the heterochromatin in Arabidopsis (Lambing et al, 2020a, 2020b). Unexpectedly, the accumulation of ASY1 at the densely DAPI-stained heterochromatic regions was not visibly affected by the absence of ASY3 (Figure 2, Supplemental figure 5A, red arrowheads). We quantified the signal intensity of ASY1 from the densely DAPI-stained regions with non-overexposed images at leptotene (see method) and found no difference between the wildtype and $asy3^{aacc}$ mutants, suggesting that ASY1 loading at the chromocenters is independent of ASY3 in Brassica napus (Supplemental figure 5B and C)."

BnASY3 is shown to interact with BnASY1 using a two hybrid assay, which is expected from studies in Arabidopsis.

Although this is expected, as suggested by the third reviewer, we further verified this interaction using the split-luciferase complementation assay.

The potentially most interesting aspect of the study comes from crossover estimates. First this is performed using chiasma scoring. First comparing aacc with wild type an expected large increase in univalents is observed. However, in aaCc mutants the authors report an increase in ring type bivalents consistent with an increase in crossovers. However, the proportion of ring to rod bivalents is a relatively imprecise method of scoring crossovers and can for example, underestimate closely spaced crossovers.
To provide additional insight the authors stain for HEI10, a protein that

localises at Class I crossover sites. As expected, the number of HEI10 foci is reduced in aacc compared to wild type. Consistent with the chiasmata counts, the authors find that in aaCc HEI10 foci are increased. The authors then estimate class II crossovers indirectly by scoring bivalents that lack HEI10 foci, the authors conclude that Class II events are normal in aaCc, and therefore the effects seen in this background are only on Class I. Finally the authors observe clustered HEI10 foci in aaCc mutants more than wild type, suggesting a defect in crossover interference.

Would the authors expect the same results as aaCc in an aAcc background?

To address this question, we isolated the mutant alleles of $asy3^{AAcc}$ and $asy3^{Aacc}$ by crossing the $asy3^{aaCc}$ mutants to wildtype. While $asy3^{AAcc}$ mutants showed a wild-type protein level of ASY3 as in $asy3^{ccCC}$, a significant reduction of ASY3 was observed in $asy3^{Aacc}$, as that in $asy3^{aaCc}$. We then analysed the crossover formation by scoring the chiasma and HEI10 foci, which revealed high parallels for $asy3^{AAcc}$ with $asy3^{aaCC}$ and $asy3^{Aacc}$ with $asy3^{aaCc}$. For more details, please see the revised manuscript.

Equally, would they expect the same results in as ASY1 aaCc mutant?

This is a good question. We unfortunately did not study the dosage effects of ASY1 in Brassica napus. Nonetheless, we would not expect the same results for $asy1^{aaCc}$ or $asy1^{Aacc}$ as in $asy3^{aaCc}$ and $asy3^{Aacc}$. We have discussed this point in the discussion session of the manuscript according to a recent study on the dosage effects of ASY1 in tetraploid wheat (Dio et al, 2023). In contrast to the series of asy3 allelic mutants reported in our manuscript, those asy1 mutants in tetraploid wheat, display a linear reduction in chiasmata (COs) concomitantly with the decrease of ASY1 gene dosage (Dio et al, 2023). In those wheat mutants with only one functional allele of ASY1, distal COs prefer to be formed at the expense of proximal and interstitial COs, supporting the conclusion that ASY1 functions to promote CO formation away from the chromosome ends (Dio et al, 2023; Lambing et al, 2020a).

ASY1 has been shown to be a key player for crossover formation. We think that comparing to the direct manipulation of ASY1 dosages, one potential advantage/difference is that the loading of ASY1 to the centromeric and pericentromeric regions would not be affected by the manipulation of ASY3, at least in Brassica napus, which is thought to be important for antagonizing the telomere-led recombination to distribute COs more evenly along the chromosome arms (Lambing et al, 2020). In addition, the ASY3-independent loading of ASY1 on the rest chromosome regions might also play an important role in the elevation of COs in $asy3^{aaCc}$ and $asy3^{Aacc}$, which can be fully

guaranteed in asy3 mutants expressing likely the similar amount of ASY1 proteins as in wildtype, but possibly not in asy1 alleles due to the reduction of total ASY1 proteins.

Minor points:

The first three paragraphs of the introduction are inadequately referenced.

We have provided relevant references in the revised manuscript.

Lines 75-79. Its not clear which mutants this statement is referring to - for example cohesin is described in the preceding sentences, but this statement would not apply to a rec8 mutant for example.

We have rephased this sentence and write now 'Plants lacking the axis components, e.g., ASY1, ASY3, ASY4, or the α-kleisin subunit of cohesin REC8, show univalents in metaphase I meiocytes and/or altered CO distribution, highlighting the importance of chromosome axis for CO patterning (Lambing et al, 2020b; Sanchez-Moran et al, 2007; Ferdous et al, 2012; Daniel et al, 2011; Dio et al, 2023; Boden et al, 2009; Cuacos et al, 2021; Chambon et al, 2018; Pochon et al, 2022; Ross et al, 1997).'

Line 241 - remove 'a bit'.

We have removed it.

Line 516 - reference citation seems to be after period.

We have corrected this.

Reviewer #3 (Comments for the Author):

During meiosis, the number and distribution of crossovers (COs) are tightly controlled. Synaptonemal complex is dispensable for CO formation and has a key role in regulating their number and distribution, imposing CO interference and heterochiasmy in Arabidopsis. In this manuscript, the authors reported results of ASY3, the axis protein and its diverse dosage control on meiotic crossover formation in Brassica napus. The authors showed that the absence of all four ASY3 alleles results in defective synapsis and drastic reduction of crossovers, which is largely rescued by the presence of only one functional ASY3 allele. These findings implied that ASY3 dosage has a strong effect on chromosome

synapsis, crossover number and distribution.

This is an interesting paper that addresses the role of a conserved chromosome axis protein, ASY3, in meiotic recombination in a polyploid plant (Brassica napus). However, the task itself is not novel. The conclusions are to some extent expected as HEI10 dosage is known to regulate levels of homologous recombination in Arabidopsis (Ziolkowski 2017). It may also be worth mentioning that the number of crossovers formed between homoeologous responds to MSH4 dosage in Brassica napus (Adrián Gonzalo 2019). There are also a number of major and minor issues with the paper that the authors should address.

We thank this reviewer for his/her time to review and comment on our paper. We also would like to thank this reviewer for his/her great suggestions that we think have helped to improve the manuscript.

Major:

1. Line 133-136, the authors suggested that they failed to obtain such mutants of asy3AAcc and asy3Aacc. Can the authors give a deeper reason rather than simply describing the phenomenon?

We agree with the reviewer that it would be a great comparison to characterize the crossover formation in such combinations of asy3^{AAcc} and asy3^{Aacc}. The reason that we initially did not obtain these two types of mutants is because that from 30 T0 transformants we obtained, the two alleles of ASY3 from A sub-genome harbor either no mutation or homozygous mutation. Therefore, we selected two independent mutant lines of asy3aaCc from the T0 plants to propagate, and the mutants of asy3^{aacc} and asy3^{aaCC} were identified from the progenies of asy3^{aaCc} mutants.

We understand that the other combinations of asy3^{AAcc} and asy3^{Aacc} can be obtained by crossing the *asy3aaCc to the wildtype, which we had immediately initiated the crossing when we realized that reducing ASY3 dosage has a potential to increase the crossovers in polyploids. However, this takes quite some time (3-4 generations to isolate the asy3^{AAcc} and asy3^{Aacc} mutants and obtain enough materials for proper characterization). Therefore, we were unable to include the characterization of asy3^{AAcc} and asy3^{Aacc} mutants in our previous manuscript.*

We have now included the characterization of asy3^{AAcc} and asy3^{Aacc} mutants, which shows a large parallel with that in asy3^{aaCC} and asy3^{aaCc} mutants, respectively. These new results reveal the functional redundancy of ASY3 from the A and C sub-genomes, and substantiate the finding that ASY3 modulates the formation of class I crossovers in a dosage-dependent manner. For more

details, please see the description in the revised manuscript.

Are there specific sequences of BnaA05.ASY3 and BnaC04. ASY3 respectively? It would be useful to obtain such mutants by designing specific targets of the C sub-genome harbor to obtain asy3AAcc, which can cross with asy3aacc to obtain asy3Aacc.

As mentioned above, we have identified the asy3^{AAcc} and asy3^{Aacc} mutants by crossing the asy3aaCc to the wildtype.

Meanwhile, the transcriptional behavior of BnaA05.ASY3 and BnaC04.ASY3 during male meiosis should be analyzed.

As suggested, we performed quantitative PCR to analyze the transcription level of BnaA05.ASY3 and BnaC04.ASY3 in anthers at the meiotic stage, and found that BnaC04.ASY3 shows a higher expression than BnaA05.ASY3, which is consistent with the transcriptome data from the Brassica napus muti-omics information resource (BnIR, https://yanglab.hzau.edu.cn/BnIR) (Liu et al, 2021) (see Supplemental figure 1B and C).

Liu, D. *et al.* BnTIR: an online transcriptome platform for exploring RNA-seq libraries for oil crop Brassica napus. *Plant Biotechnol. J.* **19**, 1895–1897 (2021).

Additionally, the progression of male meiosis should be analyzed in asy3AAcc. Only an asy3aaCC -like meiosis observed in asy3AAcc can indicate that BnaA05.ASY3 and BnaC04.ASY3 are both equally functional and able to complement one another.

Yes, we have analyzed the male meiosis in asy3^{AAcc}, which largely resemble that in asy3^{aaCC}, indicating the equal function of BnaA05.ASY3 and BnaC04.ASY3. For more details, please see the description in the revised manuscript.

2. Why was only ASY3 investigated/reported on? What about other synaptonemal complex genes? The authors need to provide some more justification for why only ASY3 was investigated in the introduction, or if other genes were also considered (at least bioinformatically) then this information should be in the results or supplementary information. There must have been some rationale based on functionality etc., unless all the synaptonemal complex genes mentioned were investigated and then this one turned out to be the key candidate.

We in general are interested in the function and regulation of the chromosome axis for patterning recombination.

Based on previous studies, the mutation of the axis component REC8 in Brassica napus would likely lead to severe defects in DSB repair, which is unlikely a good target for the manipulation of crossovers in crops.

ASY1 has been shown to be a key player for crossover formation, whereas ASY3 functions largely through recruiting ASY1 to the axis (Ferdous et al, 2012; Cuacos et al, 2021; Lambing et al, 2020; Pochon et al, 2022). Consistently, the bivalent formation is more severely compromised in asy1 mutants compared to that in asy3 (Ferdous et al, 2012).

Therefore, ASY1 seems to have a more direct effect on recombination than ASY3. This is also consistent with a recent study in tetraploid wheat showing that the chiasma number shows a linear reduction concomitantly with the decrease of ASY1 gene dosage (Dio et al, 2023). The mutation of asy3 has likely no impact on the expression/abundance of ASY1 proteins except for affecting its chromosomal recruitments, which provides a different situation for ASY1 localization compared to the global decrease of ASY1 dosage by mutating ASY1 alleles. Therefore, we designed this research to investigate the dosage effects of ASY3 on crossover frequency.

We have now discussed the rationales in the introduction and discussion parts.

Ferdous, M. *et al.* Inter-Homolog Crossing-Over and Synapsis in Arabidopsis Meiosis Are Dependent on the Chromosome Axis Protein AtASY3. *PLOS Genetics* **8**, e1002507 (2012).

Cuacos, M. *et al.* Meiotic chromosome axis remodelling is critical for meiotic recombination in Brassica rapa (2021). *J Exp Bot:* erab035

Lambing C, Kuo PC, Tock AJ, Topp SD & Henderson IR (2020a) ASY1 acts as a dosage-dependent antagonist of telomere-led recombination and mediates crossover interference in Arabidopsis. *Proc National Acad Sci* 117: 13647–13658

Pochon G, Henry IM, Yang C, Lory N, Fernández-Jiménez N, Böwer F, Hu B, Carstens L, Tsai HT, Pradillo M, et al (2022) The Arabidopsis Hop1 homolog ASY1 mediates cross-over assurance and interference. *PNAS Nexus* 2: pgac302

Dio CD, Serra H, Sourdille P & Higgins JD (2023) ASYNAPSIS 1 ensures crossover fidelity in polyploid wheat by promoting homologous recombination and suppressing non-homologous recombination. *Front Plant Sci* 14: 1188347

3. Line 357-360, "...non-HEI10 labeled chiasmata were treated as class II COs." This statement is seemingly inappropriate, because HEI10 is the only protein of catalyzing class I COs. Are there markers indicating Class II COs to measure class II COs more accurately?

We agree with this reviewer that this method is not perfect and may underestimate the amount of class II COs. Unfortunately, there is no cytological marker to indicate class II COs so far.

By keeping this reviewer's comment in our heart, we now have expressed a warning that this represents an estimation of the number of class II COs and that this number could be underestimated due to the incapacity to recognize the closely localized COs.

Considering that this estimation is likely not against by the first and second reviewers, we hope this reviewer and editor would agree with us that our approach is similar to the accepted way of the estimation of chiasma number, which is not perfect yet still meaningful. Thus, we would like to propose to keep these data unless this reviewer and editor strongly against it.

4. Figure 7E: the authors performed a distribution analysis for HEI10 focus number per bivalent at diakinesis by cytology suggesting that CO interference is compromised when ASY3 dosage is reduced. Whether the location of the very rare crossovers which was significantly distinct from the Poisson distribution that are formed in the asy3 completely defective allohaploid mutants?

In asy3^{aacc} mutants, the formation of HEI10 focus and bivalent at diakinesis are strongly compromised, and more than one HEI10 focus was never observed on each bivalent. In this case, we analyzed the distribution of chiasmata per cell for asy3^{aacc}. We found that while the numerical distribution of chiasmata differs significantly from a predicted Poisson distribution in the wildtype, a near random distribution was observed in asy3^{aacc}.

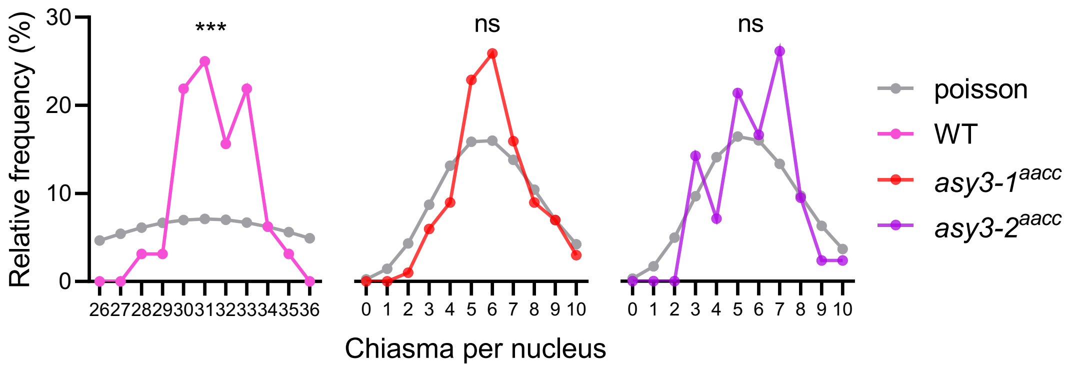

It would be better for the CO identification and analysis in the asy3 by generating segregating populations and whole-genome sequencing.

We fully agree with this reviewer that a genome-wide CO analysis by whole-genome sequencing would offer a higher resolution for CO distribution, providing a great complement and further confirmation to our current cytological analyses, as what has been done in Arabidopsis meiotic mutants. We have kept this reviewer's comment in our heart and tried to achieve such an analysis.

To perform the genome-wide CO analysis, the series of asy3 mutants in a second genetically distinct accession of Brassica napus (e.g., a semi-winter or winter-type) need to be created by CRISPR/Cas9 and then cross to the mutants generated in the accession of Westar (a spring-type), followed by a backcrossing to get the populations for sequencing.

During the progression of this project and this revision, we have tried three times to create the series of asy3 mutants in another genetic background for such a genome-wide CO analysis, which was unfortunately failed due to the failure of the transformation at the step of callus induction. We also did not notice other work reporting a genome-wide CO analysis in the mutant background of Brassica napus.

Unlike the efficient floral dipping method in Arabidopsis, the genetic transformation of Brassica napus using the hypocotyl as the explants is still difficult and has a strong dependency on their genetic backgrounds, e.g., the spring-type Westar is transformable in our hands, but not any other common winter-type accessions (e.g., Darmor-bzh).

The cytological approaches used in this study has been accepted in many literatures for counting the CO number and evaluating their distribution and interference, though not as quantitative as the whole-genome sequencing. We hope this reviewer and the editor would kindly agree that our cytological data, i.e., the elevated number of HEI10 foci at both late pachytene and diakinesis, the shorter distance of inter-HEI10 foci on each bivalent, and a Poisson-like random distribution of HEI10 foci, sufficiently support our main finding that ASY3 modulates the crossover rate and interference in a dosage-dependent manner.

A few minor comments are listed below:
1. Line 166-169, in protein interaction studies one yeast-two hybrid (Y2H) positive result cannot rule out the possibility of false positives. It is prudent to show at least one more results such as Co-Immunoprecipitation (Co-IP), bimolecular fluorescence complementation (BiFC) or Split-luciferase Complementation Imaging (LCI).

As suggested, we have now performed the split-luciferase complementation

assay as a second method to test for protein-protein interactions and can show that ASY1 binds to the N-terminal closure motif of ASY3 in Brassica napus corroborating our Y2H results.

2. Line 172-173 and 311-312, the authors used Arabidopsis asy1-4 and hei10-2 mutants to verify the specificity of the ASY1 and HEI10 antibodies. Based on Figure 6A, HEI10 foci can be observed in the cytoplasm, which makes it doubtful for the specificity of the HEI10 antibody. Can the authors use Brassica napus asy1 and hei10 mutants to verify the specificity of the ASY1 and HEI10 antibodies? Please prove it.

We agree with this reviewer that it would be ideal to test the specificity of antibodies in relevant mutants of Brassica napus. Unfortunately, due to the nature of polyploidy and lacking large-scale mutant collection and identification, the meiosis research in Brassica napus is relatively lagging compared to the diploid model species, e.g., Arabidopsis and rice, and the Brassica napus asy1 and hei10 mutants have not been identified so far. That is the reason that we used the Arabidopsis mutants.

Regarding the point that some signal/foci can be observed in the cytoplasm in the HEI10 immunostaining, while we cannot fully exclude the possibility that some signal might represent the HEI10 proteins present in the nucleoplasm or cytoplasm, we think these few signals localizing outside of chromosomes, reflect more likely the nature of the immunostaining experiments where the presence of background/unspecific signals cannot be fully avoided. In our analyses, the HEI10 foci localizing only on chromosomes were counted. Considering that with our antibodies, both ASY1 and HEI10 in Brassica napus follow a similar localization dynamics as what has been reported in Arabidopsis and other plants, e.g, ASY1 shows specific removal at the synaptic regions, and HEI10 forms numerous foci at early prophase I and progressively decreases and co-localizes with the position of chiasma at diakinesis, and that we did not observe the presence of ASY1 and HEI10 in somatic nuclei of Brassica napus, we hope this reviewer and the editor would agree that the ASY1 and HEI10 antibodies used in this research are valid for this study.

3. Line 232-237, using DAPI-staining to estimate the chromosome juxtaposition is not accurate, maybe you can observe chromosome behaviors with antibodies or probes?

As suggested, we have further re-evaluated the chromosome juxtaposition using the co-immunostaining of ZYP1 and REC8 as shown in Figure 5 (see the session 4 of the results) and at the same time, *we expressed a warning by writing that "Noting that the ratio of cells with properly/fully co-aligned chromosomes could be overestimated due to the low resolution of the DAPI-*

stained chromosomes."

4. Line 294-295, in the asy3-1aaCc and asy3-2aaCc mutants, only ~20 cells were observed to estimate the number of chiasmata, while the increase of the ratio in the ring-type bivalents is susceptible. More cells should be observed to support the point.

As suggested, we have performed new chromosome spread experiment and quantified more cells (51 cells for asy3-1^{aaCc} cells, 44 cells for asy3-2^{aaCc}, 53 cells for asy3-1^{Aacc}, and 40 cells in asy3-2^{Aacc}), and the new result is consistent with our previous analysis, supporting the increase of the ratio of ring-type bivalents in asy3^{aaCc} and asy3^{Aacc} mutants*.*

1st Revision - Editorial Decision09-Jun-2024

Dear Dr. Yang:

We have received reviews of your manuscript entitled "ASY3 has dosage-dependent diverse effects on meiotic crossover formation." On the basis of the advice received, the board of reviewing editors would like to accept your manuscript for publication in The Plant Cell. This acceptance is contingent on revision based on the comments of our reviewers. In particular, please consider the following::

Reviewers 1 and 2 were satisfied by your revisions. Reviewer 3 still has some concerns. We realize that it might take too long to address the request of genetic mapping with markers in the mutant background and while it would be nice to have, we will not require that. However, if you could address the other points, that would further improve the manuscript.

Please highlight all changes and include a detailed annotation of changes of the text, with line numbers, and noting your responses to the comments.

To submit your revised manuscript, click:

Link Not Available

If you have any questions about the revision submission procedures, please contact the Editorial Office Staff (tpc-submissions@aspb.org). If you cannot return the revised manuscript within 30 days, please let us know. Otherwise, we will assume that you have elected not to revise the manuscript and withdraw it.

Thank you very much for the privilege of reviewing this work. I look forward to receiving the next version.

On behalf of the editorial board,

Reviewing Editor, Board of Reviewing Editors
James Birchler, Senior Editor
Blake Meyers, Editor-in-Chief

The Plant Cell

Reviewer #1 (Comments for the Author):

The authors have responded excellently to the reviewers comments and the manuscript has been clearly improved.

Reviewer #2 (Comments for the Author):

The reviewers have responded to my concerns adequately.

Reviewer #3 (Comments for the Author):

Remarks to the Author:

I do appreciate the authors attention to revising the manuscript and I believe the revisions have made it better.

In my first review, my main comment concerned the CO identification and distribution in the asy3. I proposed that generating segregating populations and performing a genome-wide CO analysis by whole genome sequencing could help to understand CO assurance and interference modulated by ASY3. In their response, the authors gave a few reasons that convinced me that a genome-wide CO analysis was not necessary for the mutant background of Brassica napus. Particularly, the genetic transformation of Brassica napus has a strong dependency on their genetic backgrounds. I understand that it can be challenging as an asy3 mutant needs to be created in other common winter-type background and difficult to do due to time constraints. However, measuring recombination frequency and distribution by genetic mapping using molecular marker analysis has been widely used not only performed in Arabidopsis, but also in barley (Miriam Schreiber 2022) and wheat which is tetraploid and hexaploidy (Stuart D. Desjardins 2022). I still think this is required to support the conclusion about the influence on CO interference and number with different ASY3 dosage in asy3 mutants. I also believe that the recombination landscape would provide us a much deeper understanding of crossover control of ASY3. Anyway, backcrossing the mutants into the other

backgrounding may be an effective way to generate segregating populations.

Besides, the authors make the point that chromosome synapsis is sensitive to ASY3 dosage. In Fig 5, a complete SC assembly was never observed in asy3aaCc and asy3Aacc, while the asy3aaCC and asy3AAcc showed complete ZYP1 assembly. However, it is not descriptive enough for the localization of ZYP1 and REC8 in the asy3aacc. It is also well known that ASY3 is an ortholog of Arabidopsis ASY3, rice PAIR3 and maize DSY2, which all localize on chromosome axes during meiosis (Wang 2011; Ferdous 2012; Ding Hua Lee 2015). Whether the central element protein ZYP1 is located between two ASY3-labeled Les? It wound be necessary to perform an immunolocalization of ASY3 together with other SC proteins, including ASY1 and ZYP1 using super-resolution SIM on wild-type and asy3 nuclei, which enabled the visualization of the SC structure (Wang 2009). It will help readers to understand better for the point that continuous tripartite structure of the SC is critical for proper HEI10 diffusion and CO interference.

Also generally, it has been suggested in many species that DSB formation is indispensable for meiotic recombination (Barzel and Kupiec 2008). It was previously reported that the loss of ASY3 interrupts DSB formation in Arabidopsis. There is also a similar decline in DSBs at in the dsy2 suggesting that DSY2 has a similar influence on DSB formation in maize. However, this is not discussed in depth in this manuscript. I also suggest authors conducting the TUNEL assays or examining the distribution of RAD51 in the asy3 mutants. This might explain the marked increase of the class I COs in asy3aaCc and asy3Aacc.

In summary, the authors reported an interesting finding that the dosage-dependent distinct effects of ASY3 on crossover formation. However, unfortunately, the underlying molecular mechanisms remain unclear, and no reasonable explanations were provided to explain this phenomenon. To make this data reliable for reporting in a scientific journal, at least not just cytology experiments were performed which should be combined with NGS data.

Minor comment:
1. Line 291 and 301, "Figure 5A" should be "Figure 5"

Please also note the following:

-The Plant Cell now requires authors to complete and submit an author revisions checklist upon submission of a revised manuscript. The aim of the checklist is to aid authors in preparing a high-quality manuscript, facilitate the review and assessment of revised manuscripts, and help to ensure that journal standards are maintained across the board. If your manuscript is accepted, the completed checklist will be published as supplemental material attached to the article online. Please download a copy of the checklist (pdf fillable form) at this link, for submission with your revised manuscript: https://tpc.msubmit.net/html/Author_Revisions_Checklist.pdf.

-Supplemental materials should be restricted to large datasets and tables, presentation of replicates, and validation of reagents, methods, or genotypes. Any data that are used to support the major claims must be in the main manuscript. Supplemental figure legends must indicate what figure in the main manuscript is supported by the supplemental data presented. Please justify how each of the supplemental figures meet the criteria.

-Sampling methods and the nature of "biological replicates" should be described precisely (i.e. different plants, parts of plants, pooled tissue, independent pools of tissue, sampled at different times, etc.), along with a clear description of and rationale for any statistical analyses conducted. The reader should know exactly what was sampled; what forms the basis of the calculation of any means and statistical parameters reported. This is also necessary to ensure that proper statistical analysis was conducted.

Reviewer #1 (Comments for the Author):

The authors have responded excellently to the reviewers comments and the manuscript has been clearly improved.

We thank again this reviewer for his/her time to read and comment on our revised manuscript. We appreciate very much his/her inputs that have promoted our paper.

Reviewer #2 (Comments for the Author):

The reviewers have responded to my concerns adequately.

We thank again this reviewer for his/her time to read and comment on our revised manuscript. We appreciate very much his/her inputs that have promoted our paper.

Reviewer #3 (Comments for the Author):

Remarks to the Author:

I do appreciate the authors attention to revising the manuscript and I believe the revisions have made it better.

We thank again this reviewer for his/her time to read and comment on our revised manuscript. We appreciate very much his/her constructive suggestions that have promoted our paper.

In my first review, my main comment concerned the CO identification and distribution in the asy3. I proposed that generating segregating populations and performing a genome-wide CO analysis by whole genome sequencing could help to understand CO assurance and interference modulated by ASY3. In their response, the authors gave a few reasons that convinced me that a genome-wide CO analysis was not necessary for the mutant background of Brassica napus. Particularly, the genetic transformation of Brassica napus has a strong dependency on their genetic backgrounds. I understand that it can be challenging as an asy3 mutant needs to be created in other common winter-type background and difficult to do due to time constraints. However, measuring recombination frequency and distribution by genetic mapping using molecular marker analysis has been widely used not only performed in Arabidopsis, but also in barley (Miriam Schreiber 2022) and wheat which is tetraploid and hexaploidy (Stuart D. Desjardins 2022). I still

think this is required to support the conclusion about the influence on CO interference and number with different ASY3 dosage in asy3 mutants. I also believe that the recombination landscape would provide us a much deeper understanding of crossover control of ASY3. Anyway, backcrossing the mutants into the other backgrounding may be an effective way to generate segregating populations.

We appreciate very much his/her understanding for the difficulties of generating the other series of asy3 mutants in a second genetic background. We thank this reviewer for suggesting the molecular marker-based approach to measure the CO frequency and distribution. However, while this method is very effective in the wildtype, it becomes a bit tricky in the mutant background since this method could only analyze the COs from the heterozygous chromosome regions of BC1F2 mutant plants using their BC1F3 progenies. Although we fully agree that it would be still nice to have this piece of data, we hope this review could kindly agree that backcrossing to the other genetic background to obtain the BC1F3 plants is very time consuming and thus somehow out of the timeframe of this research. We also appreciate very much the kind understanding from the editors.

Besides, the authors make the point that chromosome synapsis is sensitive to ASY3 dosage. In Fig 5, a complete SC assembly was never observed in asy3aaCc and asy3Aacc, while the asy3aaCC and asy3AAcc showed complete ZYP1 assembly. However, it is not descriptive enough for the localization of ZYP1 and REC8 in the asy3aacc. It is also well known that ASY3 is an ortholog of Arabidopsis ASY3, rice PAIR3 and maize DSY2, which all localize on chromosome axes during meiosis (Wang 2011; Ferdous 2012; Ding Hua Lee 2015). Whether the central element protein ZYP1 is located between two ASY3-labeled Les? It wound be necessary to perform an immunolocalization of ASY3 together with other SC proteins, including ASY1 and ZYP1 using super-resolution SIM on wild-type and asy3 nuclei, which enabled the visualization of the SC structure (Wang 2009). It will help readers to understand better for the point that continuous tripartite structure of the SC is critical for proper HEI10 diffusion and CO interference.

As suggested, we acquired the super-resolution images for the co-immunostaining of ZYP1 with REC8/ASY3 using the STED microscope. Although the fact that our ZYP1 antibody recognizes only its C-terminal region makes it challenging to separate the axis with ZYP1 signals, the STED images show clearly that the central filament protein ZYP1 is located between the REC8/ASY3-labeled axes (see Supplemental figure 9).

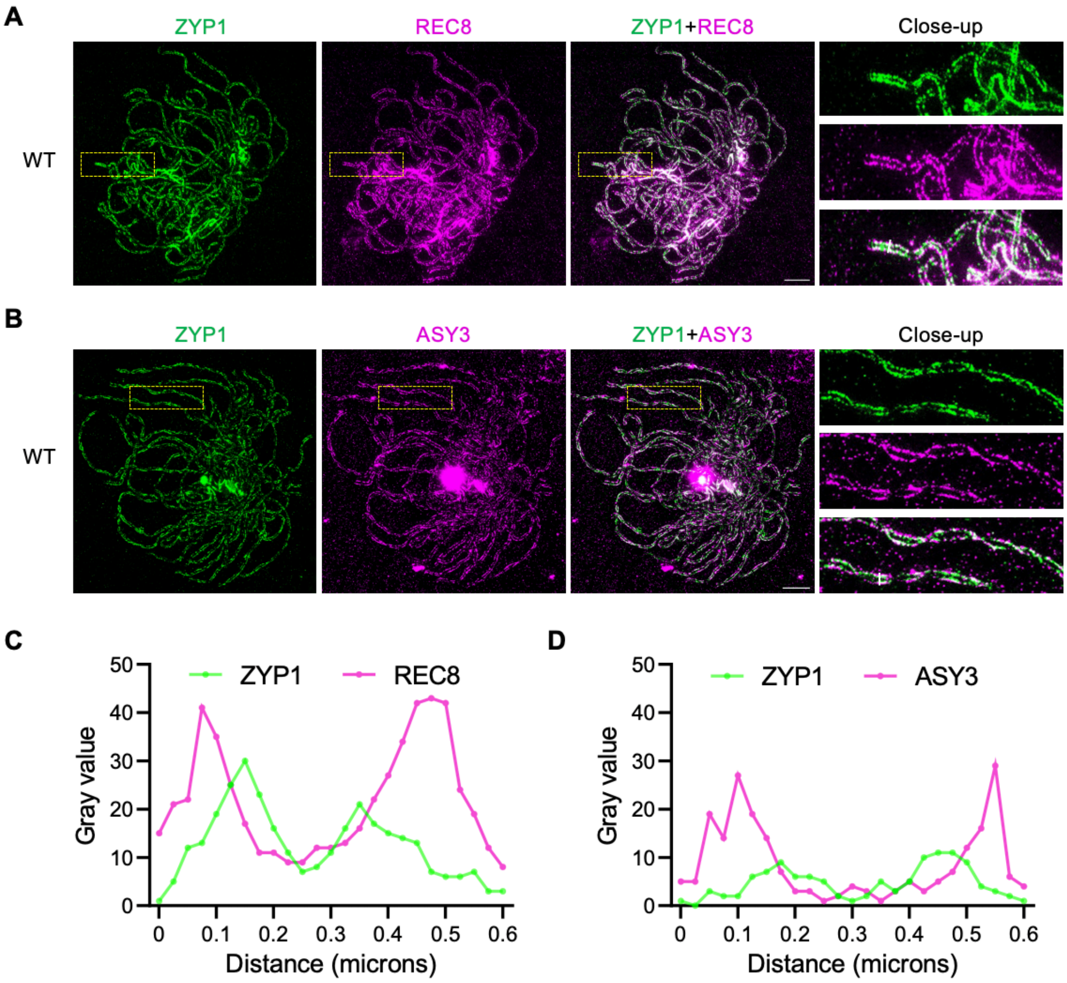

Also generally, it has been suggested in many species that DSB formation is indispensable for meiotic recombination (Barzel and Kupiec 2008). It was previously reported that the loss of ASY3 interrupts DSB formation in Arabidopsis. There is also a similar decline in DSBs at in the dsy2 suggesting that DSY2 has a similar influence on DSB formation in maize. However, this is not discussed in depth in this manuscript. I also suggest authors conducting the TUNEL assays or examining the distribution of RAD51 in the asy3 mutants. This might explain the marked increase of the class I COs in asy3aaCc and asy3Aacc.

As suggested, we analyzed the DSB formation in the series of asy3 mutants of Brassica napus by examining the localization of DMC1 that relies strictly on the DSBs (See Supplemental figure 12). We found a similar decrease of DSBs in the absence of asy3^{aacc} (~ 23% decrease) as that in Arabidopsis asy3 mutants, indicating the importance of ASY3 for the proper formation of DSBs. Unexpectedly, we found that the DSB formation in asy3^{aaCC}, asy3^{AAcc}, asy3^{aaCc}, and asy3^{Aacc} is similar to that in the wildtype, implying that DSB formation is not

very sensitive to the dosage decrease of ASY3. For more details, please see the relevant result part in the manuscript.

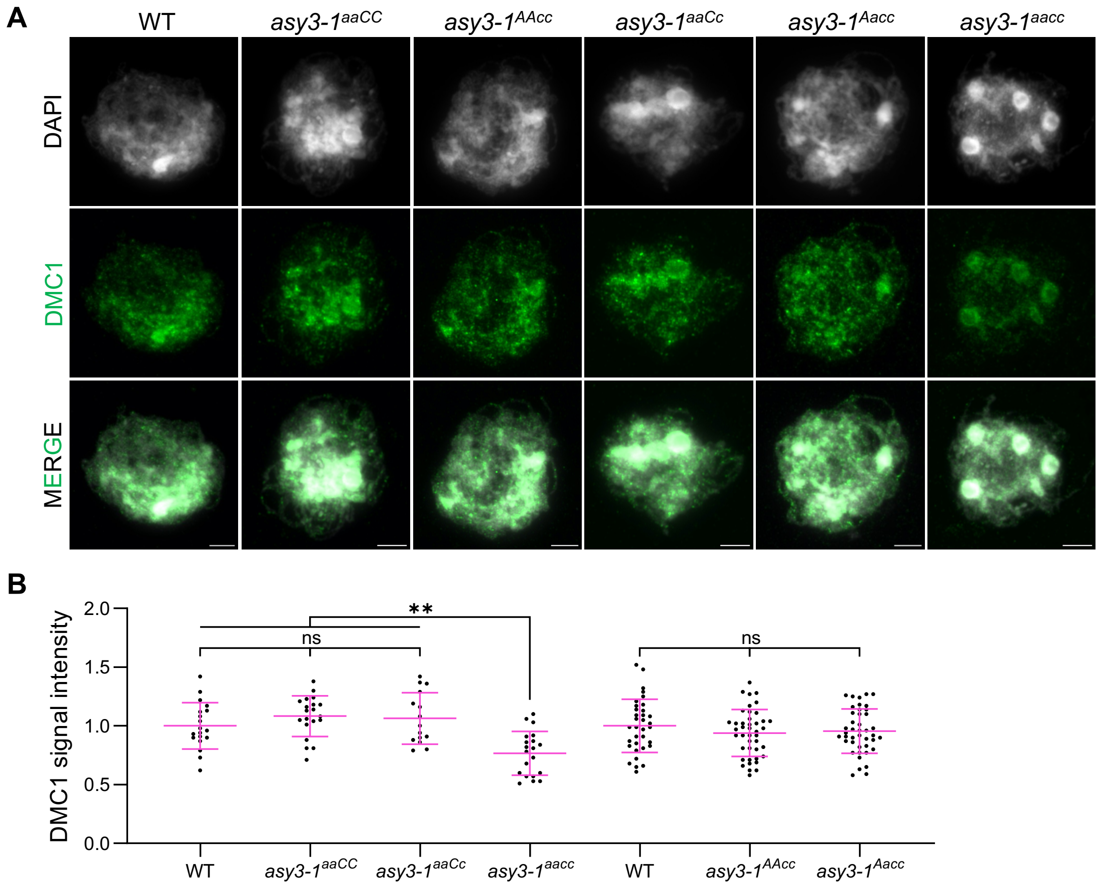

In summary, the authors reported an interesting finding that the dosage-dependent distinct effects of ASY3 on crossover formation. However, unfortunately, the underlying molecular mechanisms remain unclear, and no reasonable explanations were provided to explain this phenomenon. To make this data reliable for reporting in a scientific journal, at least not just cytology experiments were performed which should be combined with NGS data.

See response above.

Minor comment:
1. Line 291 and 301, "Figure 5A" should be "Figure 5"

we corrected this as suggested.

Dear Dr. Chao Yang:

We are pleased to inform you that your paper entitled "ASY3 has dosage-dependent diverse effects on meiotic crossover formation" has been accepted for publication in The Plant Cell, pending a final editorial review by a science editor. At this stage, your manuscript will be evaluated by a science editor with respect to its presentation of scientific content, compliance with journal policies, and presentation for a broad readership. The Plant Cell has contracted with Plant Editors (planteditors.com) to provide this service to our authors, and you will soon receive additional information on this process.

Please note that each author needs to link their ORCID identifier to their account in the system before your manuscript can be published. If any authors do not have an ORCID linked to their account, they will receive a message with a link to complete this task. Please ensure that ALL of your coauthors have completed this task as soon as possible.

ASPB offers an OPEN option that allows authors to have their online articles available for free to all users immediately upon publication. For more information about the ASPB OPEN option, refer to the Final Submission Checklist Form.

The Plant Cell and The Arabidopsis Information Resource (TAIR) are collaborating to collect functional annotation data about Arabidopsis genes from authors. This includes information about the gene's molecular function (e.g., kinase activity, ATP synthetase activity), the biological process/es it is involved in (e.g., endosperm development, threonine biosynthesis), its subcellular location (e.g., nucleus, ER), anatomical or developmental expression pattern (e.g., leaf, ovule, flower stage 10, seedling stage), or its partner in a protein-protein interaction (e.g., AT1G01010 interacts with AT1G01020).

If your paper contains results falling into one or more of these categories for Arabidopsis genes, we request that you now submit these data for inclusion in TAIR by filling in the form provided at the following URL: https://www.arabidopsis.org/doc/submit/functional_annotation/123. If you need further clarification on what types of data can be submitted please contact curator@arabidopsis.org.

Finally, we encourage your submission of artwork for the journal cover. Monthly issues will have an online cover image and selected covers will be used for posters, other promotional items, and "wallpaper" for mobile devices. For more information, refer to Cover Submission in the Instructions for Authors https://tpc.msubmit.net/cgi-bin/main.plex?form_type=display_auth_instructions.

We look forward to seeing your paper published.

On behalf of the editorial board,

Reviewing Editor, Board of Reviewing Editors
James Birchler, Senior Editor
Blake Meyers, Editor-in-Chief

The Plant Cell

=====
IMPORTANT REMINDER: PEER REVIEW REPORTS
=====

If you opted to publish a peer review report along with your article during the original submission process, it will be prepared by the editorial staff and publicly posted with your manuscript, inside the zip file that contains any other supplemental material. As a reminder, the peer review report is a public record of all comments from editors and reviewers, as well as your prior responses, as you received them in the decision letters for each draft of your manuscript. If you agreed to publish this report and have changed your mind, or are not sure if you selected this option, please contact the editorial office as soon as possible before signing the license agreement from our publisher.

----FOR ASPB OFFICE USE ONLY (DO NOT EDIT)----
MSID: 38389
Corresponding Author: Dr. Yang
